# Supplementary material for: Polymer cyclization for the emergence of hierarchical nanostructures
Source: Nat Commun. 2021 Jun 25;12:3959. doi: 10.1038/s41467-021-24222-5 (PMC8233313; doi:10.1038/s41467-021-24222-5)
Supplement: Supplementary file 1 — Supporting information. [file 41467_2021_24222_MOESM1_ESM.pdf]

Supplementary Information

Polymer cyclization for the emergence of hierarchical nanostructures

Chaojian Chen, Manjesh Kumar Singh, Katrin Wunderlich, Sean Harvey, Colette Whitfield, Zhixuan Zhou, Manfred Wagner, Katharina Landfester, Ingo Lieberwirth, George Fytas, Kurt Kremer, Debashish Mukherji, David Y. W. Ng,* and Tanja Weil*

Correspondence to: david.ng@mpip-mainz.mpg.de (D.Y.W.N.);
weil@mpip-mainz.mpg.de (T.W.)

Table of Contents

Supplementary Methods **3**
1. Materials **3**
2. Synthesis and folding of linear PHEMA **3**
2.1 Synthesis of *l*-PHEMA$_n$-*Br* via atom transfer radical polymerization 3
2.2 Synthesis of *l*-PHEMA$_n$-N_3 via azidation of *l*-PHEMA$_n$-*Br* 3
2.3 Synthesis of *f*-PHEMA$_n$ by folding *l*-PHEMA$_n$-N_3 via click chemistry 4
3. Self-assembly of linear and folded PHEMA **4**
3.1 Self-assembly of linear and folded PHEMA 4
3.2 Nile Red loading and the determination of critical aggregation concentration (CAC) 4
4. Synthesis of amphiphilic block copolymer PS-b-PAA **5**
4.1 Synthesis of PS$_x$-*Br* via atom transfer radical polymerization 5
4.2 Synthesis of block copolymer PS$_x$-*b*-P*t*BA$_y$ via ATRP 5
4.3 Synthesis of PS$_x$-*b*-PAA$_y$ by hydrolysis of *tert*-butyl ester groups of P*t*BA block 5
5. Synthesis of linear brush polymers with amphiphilic block side chains **6**
5.1 Synthesis of linear macroinitiator *l*-P(HEMA-*Br*)$_{22}$ 6
5.2 Synthesis of linear brush polymer *l*-P(HEMA-*g*-PS$_x$-*Br*)$_{22}$ 6
5.3 Synthesis of linear brush polymer *l*-P(HEMA-*g*-PS$_x$-*b*-P*t*BA$_y$)$_{22}$ 7
5.4 Synthesis of amphiphilic linear brush polymer *l*-P(HEMA-*g*-PS$_x$-*b*-PAA$_y$)$_{22}$ 7
6. Synthesis of cyclic brush polymers with amphiphilic block side chains **8**
6.1 Synthesis of cyclic macroinitiator *f*-P(HEMA-*Br*)$_{22}$ 8
6.2 Synthesis of cyclic brush polymers *f*-P(HEMA-*g*-PS$_x$-*Br*)$_{22}$ 8
6.3 Synthesis of cyclic brush polymers *f*-P(HEMA-*g*-PS$_x$-*b*-P*t*BA$_y$)$_{22}$ 9
6.4 Synthesis of amphiphilic cyclic brush polymers *f*-P(HEMA-*g*-PS$_x$-*b*-PAA$_y$)$_{22}$ 9
7. Self-assembly of block copolymers, linear and cyclic brush polymers **9**
8. Characterization **10**
8.1 Nuclear magnetic resonance (NMR) spectroscopy 10
8.2 Fourier-transform infrared (FTIR) spectroscopy 11
8.3 Gel permeation chromatography (GPC) 11
8.4 Dynamic light scattering (DLS) 11
8.5 Transmission electron microscopy (TEM) 11
8.6 Atomic force microscopy (AFM) 12
9. All-atom molecular dynamics simulations **12**
10. Generic molecular dynamics simulations **12**
10.1 Molecular dynamics simulations for the self-assembly of folded PHEMA 13
10.2 Molecular dynamics simulations for the self-assembly of cyclic brush polymers 13
Supplementary Figures **14**
Supplementary Tables **79**
Supplementary References **82**

Supplementary Methods

1. Materials

Propargyl 2-bromoisobutyrate (PBIB, >97%), ethyl α-bromoisobutyrate (EBIB, 98%), α-bromoisobutyryl bromide (98%), copper(I) chloride (CuCl, 99.995%), copper(I) bromide (CuBr, 99.999%), *N*,*N*,*N′*,*N″*,*N″*-pentamethyldiethylenetriamine (PMDETA, 99%), 2,2'-bipyridyl (bpy, ≥99%), methyl ethyl ketone (MEK, 99.5%) and Nile Red (technical grade) were purchased from Sigma-Aldrich and used without further treatment. Sodium azide (NaN_3, 99%) was purchased from Applichem and used as received. Trifluoroacetic acid (TFA, ≥99.9%) was obtained from Carl Roth. 2-Hydroxyethyl methacrylate (HEMA, Sigma-Aldrich, 97%, ≤250 ppm monomethyl ether hydroquinone), styrene (99%, Sigma-Aldrich), and *tert*-butyl acrylate (*t*BA, 98%, Sigma-Aldrich) were passed through basic Al_2O_3 column to remove inhibitors before use. All other solvents were obtained from commercial suppliers and used as received.

2. Synthesis and folding of linear PHEMA

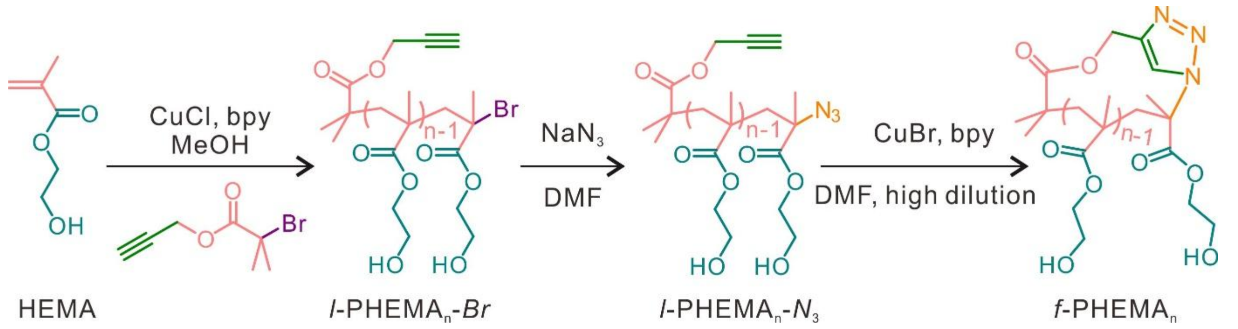

2.1 Synthesis of *l*-PHEMA$_n$-*Br* via atom transfer radical polymerization

In a typical procedure for the synthesis of *l*-PHEMA$_{22}$-*Br*, PBIB (310 µL, 2 mmol), HEMA (6.06 mL, 50 mmol) and bpy (781 mg, 5 mmol) were dissolved in 6 mL methanol in a 15 mL Schlenk flask under argon flow. Followed the mixture was degassed through three freeze-pump-thaw cycles and CuCl (238 mg, 2.4 mmol) was added to start the polymerization. After stirring at 24 °C for 5 h, the reaction mixture was quenched by exposing the reaction solution to air, followed by dilution with methanol. The solution was stirred until the color turned to blue. Then the Cu(II) catalyst was removed by passing through a silica column. The solution was concentrated and precipitated into diethyl ether. The procedure of dissolution with methanol and precipitation in diethyl ether was repeated twice. The product was obtained as a white solid after drying under high vacuum.

2.2 Synthesis of *l*-PHEMA$_n$-*N$_3$* via azidation of *l*-PHEMA$_n$-*Br*

In a typical procedure for the synthesis of *l*-PHEMA$_{22}$-*N$_3$*, sodium azide (2.44 g, 37.5 mmol) was added to a round-bottom flask containing *l*-PHEMA$_{22}$-*Br* (2.4 g, 0.79 mmol) dissolved in mix solvent of DMF/H_2O (60 mL, 4:1 v/v). The reaction mixture was stirred at 50 °C for two days

and then cooled down to room temperature. After removing most of the solvents at reduced pressure, the remaining portion was diluted with ethanol. The solid salt was removed by centrifugation. The solution was loaded into a dialysis membrane (MWCO ~ 1000 Da) and extensively dialyzed against deionized water and methanol for two days. The product was obtained as a white solid after precipitation in diethyl ether and drying under high vacuum.

2.3 Synthesis of *f*-PHEMA$_n$ by folding *l*-PHEMA$_n$-*N*$_3$ via click chemistry

Folded PHEMA (*f*-PHEMA$_n$) with a cyclic secondary structure was synthesized by folding linear polymer *l*-PHEMA$_n$-*N*$_3$ via the azide-alkyne Huisgen cycloaddition under high dilution conditions. In a typical procedure for the folding of *l*-PHEMA$_{22}$-*N*$_3$, DMF (1 L) was added to a 2.5 L round bottomed flask and then degassed by two freeze-pump-thaw cycles. CuBr (0.86 g, 6 mmol) and bpy (1.87 g, 12 mmol) were added to the frozen DMF. The flask was resealed, evacuated, and refilled with argon. A separate flask containing 0.5 g of *l*-PHEMA$_{22}$-*N*$_3$ dissolved in 10 mL of DMF was degassed by three freeze-pump-thaw cycles. This solution was then added to the catalyst reaction solution at 120 °C via a syringe pump at a rate of 0.16 mL h^{-1}. Once the polymer was finished adding to the catalyst solution, the reaction was allowed to proceed at 120 °C for additional 24 h before cooling to room temperature. Most solvent of the reaction solution was removed under reduced pressure and the remained portion was diluted with methanol and stirred until the color changed to blue. The Cu(II) catalyst was removed by passing through a silica column. The solution was concentrated and precipitated into diethyl ether. The product was finally obtained after drying under high vacuum.

3. Self-assembly of linear and folded PHEMA

3.1 Self-assembly of linear and folded PHEMA

A dialysis method was used to prepare assemblies of folded polymers. In a typical process, 21 mg of folded PHEMA was dissolved in 7 mL of methanol at room temperature. The solution was vigorously stirred and 7 mL of deionized water was added with a speed of 0.2 mL min^{-1}. After stirring for another 2 h, the solution was loaded into a dialysis membrane (MWCO ~ 1000 Da) and dialyzed against deionized water for three days to completely remove the solvent methanol. The final concentration of polymers was tuned to 1 mg mL^{-1} by adding water. For the self-assembly of linear polymers, the same procedure was employed and precipitation was observed.

3.2 Nile Red loading and the determination of critical aggregation concentration (CAC)

The CAC of micelles was determined using Nile Red as a fluorescence probe. The concentration of polymers was varied from 1 mg mL^{-1} to 1 × 10^{-4} mg mL^{-1}. Nile Red solution in acetone (15 μL, 0.02 mg mL^{-1}) was then added to 1 mL of each polymer solution. After sonication for 30 min, acetone was evaporated and the final concentration of Nile Red in each sample was therefore fixed at 3 × 10^{-4} mg mL^{-1}. Fluorescence spectra were recorded using a TECAN system (Spark 20M) at room temperature. The excitation wavelength was set as 550 nm and the emission

wavelength was monitored from 590 nm to 720 nm. Excitation and emission bandwidths were both maintained at 20 nm and the emission wavelength step size was 2 nm.

4. Synthesis of amphiphilic block copolymer PS-b-PAA

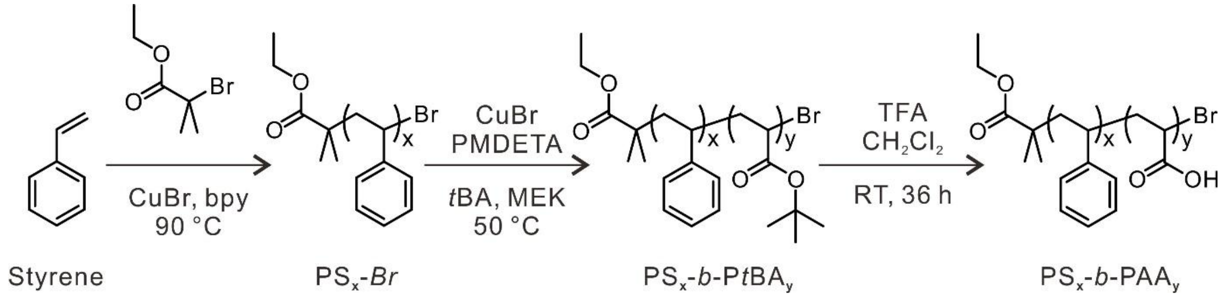

4.1 Synthesis of PS_x-*Br* via atom transfer radical polymerization

In order to synthesize PS_{40}-*Br*, EBIB (29.4 µL, 0.2 mmol), styrene (9.17 mL, 80 mmol), and bpy (62.4 mg, 0.4 mmol) were added into a 50 mL Schlenk flask and the mixture was degassed through three freeze-pump-thaw cycles. CuBr (28.6 mg, 0.2 mmol) was then added under argon flow and the polymerization was proceeded at 90 °C. After reaction for 3 h, the polymerization was stopped after putting the flask into liquid nitrogen. THF was added and the reaction mixture was then passed through a short column filled with neutral Al_2O_3 to remove the copper catalyst. The crude product was precipitated in cold methanol. The procedure of dissolution with THF and precipitation in methanol was repeated twice and the product was obtained after drying under high vacuum.

4.2 Synthesis of block copolymer PS_x-*b*-P*t*BA_y via ATRP

In a typical procedure for the synthesis of block polymer PS_{40}-*b*-P*t*BA_{12}, PS_{40}-*Br* (172 mg, 0.04 mmol initiation sites), *t*BA (2.34 mL, 16 mmol), and PMDETA (36 µL, 0.16 mmol) were dissolved in 2.34 mL MEK in a Schlenk flask and the mixture was degassed through three freeze-pump-thaw cycles. CuBr (11.5 mg, 0.08 mmol) was then added under argon flow and the polymerization was proceeded at 50 °C. After reaction for 20 min, the polymerization was stopped by putting the flask into liquid nitrogen. Acetone was added and the reaction mixture was then passed through a short column filled with neutral Al_2O_3 to remove the copper catalyst. The crude product was precipitated in methanol/water (v/v=1/1) and used for the next hydrolysis step.

4.3 Synthesis of PS_x-*b*-PAA_y by hydrolysis of *tert*-butyl ester groups of P*t*BA block

In a typical step for the synthesis of PS_{40}-*b*-PAA_{12}, 40 mg of PS_{40}-*b*-P*t*BA_{12} was dissolved in 6 mL CH_2Cl_2 followed by adding 3 mL TFA. The solution was stirred at room temperature for 36 h. After removing the solvent by evaporation, the product was dissolved in minimum amount of DMF and dialyzed against acetone and DI water (MWCO ~ 1000 Da). The final product was obtained after freeze drying.

5. Synthesis of linear brush polymers with amphiphilic block side chains

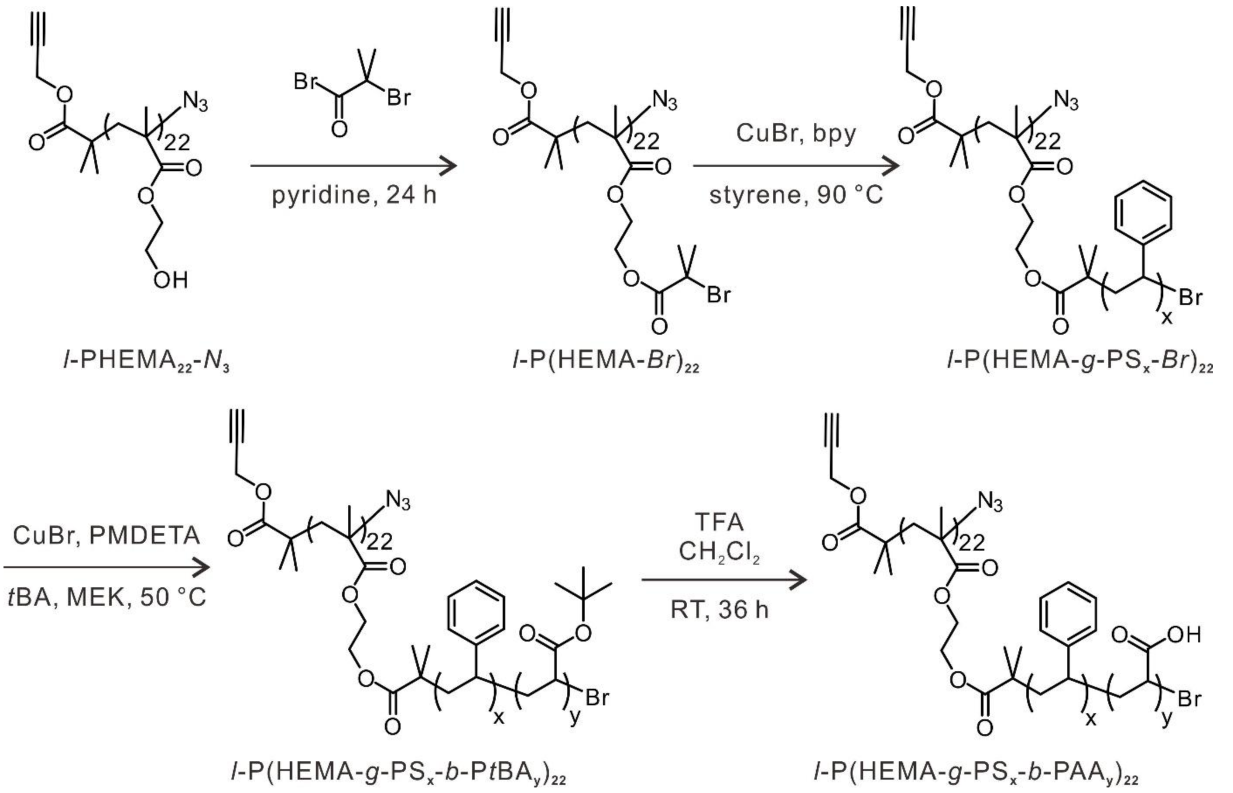

5.1 Synthesis of linear macroinitiator *l*-P(HEMA-*Br*)$_{22}$

Linear macroinitiator *l*-P(HEMA-*Br*)$_{22}$ was synthesized by esterification of *l*-PHEMA$_{22}$-N_3 with α-bromoisobutyryl bromide. Briefly, *l*-PHEMA$_{22}$-N_3 (400 mg) was dissolved in 10 mL anhydrous pyridine in a 25 mL flask. After cooling to 0 °C in an ice-water bath, α-bromoisobutyryl bromide (2 mL, 15.4 mmol) was added dropwise during 1 h. The solution was further stirred at 0 °C for 1 h, and then at room temperature for 24 h. The reaction mixture was precipitated in 100 mL cold water and then separated by centrifugation. The collected product was dissolved in a small amount of THF and reprecipitated in water. After repeating the process for two cycles, the product was obtained after drying under high vacuum.

5.2 Synthesis of linear brush polymer *l*-P(HEMA-*g*-PS$_x$-*Br*)$_{22}$

In order to synthesize linear brush polymer *l*-P(HEMA-*g*-PS$_{40}$-*Br*)$_{22}$, *l*-P(HEMA-*Br*)$_{22}$ (20 mg, 0.072 mmol initiation sites), styrene (5 mL, 43.6 mmol), and bpy (22.5 mg, 0.144 mmol) were added into a 15 mL Schlenk flask and the mixture was degassed through three freeze-pump-thaw cycles. CuBr (10.3 mg, 0.072 mmol) was then added under argon flow and the polymerization was proceeded at 90 °C. After reaction for a determined time, the polymerization was stopped after putting the flask into liquid nitrogen. THF was added and the reaction mixture was then passed through a short column filled with neutral Al_2O_3 to remove the copper catalyst. The crude product was precipitated in cold methanol. The procedure of dissolution with THF and

precipitation in methanol was repeated twice and the product was obtained after drying under high vacuum.

5.3 Synthesis of linear brush polymer *l*-P(HEMA-*g*-PS_x-*b*-P*t*BA_y)$_{22}$

In a typical procedure for the synthesis of linear brush polymer *l*-P(HEMA-*g*-PS_{40}-*b*-P*t*BA_7)$_{22}$, *l*-P(HEMA-*g*-PS_{40}-*Br*)$_{22}$ (150 mg), *t*BA (1.97 mL, 13.5 mmol), and PMDETA (31 µL, 0.135 mmol) were dissolved in 1.97 mL MEK in a Schlenk flask and the mixture was degassed through three freeze-pump-thaw cycles. CuBr (10 mg, 0.067 mmol) was then added under argon flow and the polymerization was proceeded at 50 °C. After reaction for 30 min, the polymerization was stopped by putting the flask into liquid nitrogen. Acetone was added and the reaction mixture was then passed through a short column filled with neutral Al_2O_3 to remove the copper catalyst. The crude product was precipitated in methanol/water (v/v=1/1) and used for the next hydrolysis step.

5.4 Synthesis of amphiphilic linear brush polymer *l*-P(HEMA-*g*-PS_x-*b*-PAA_y)$_{22}$

In a typical step for the synthesis of *l*-P(HEMA-*g*-PS_{40}-*b*-P*t*BA_7)$_{22}$, 60 mg of *l*-P(HEMA-*g*-PS_{40}-*b*-P*t*BA_7)$_{22}$ was dissolved in 8 mL CH_2Cl_2 followed by adding 4 mL TFA. The solution was stirred at room temperature for 40 h. After removing the solvent by evaporation, the product was dissolved in minimum amount of DMF and dialyzed against acetone and DI water. The final product was obtained after freeze drying.

6. Synthesis of cyclic brush polymers with amphiphilic block side chains

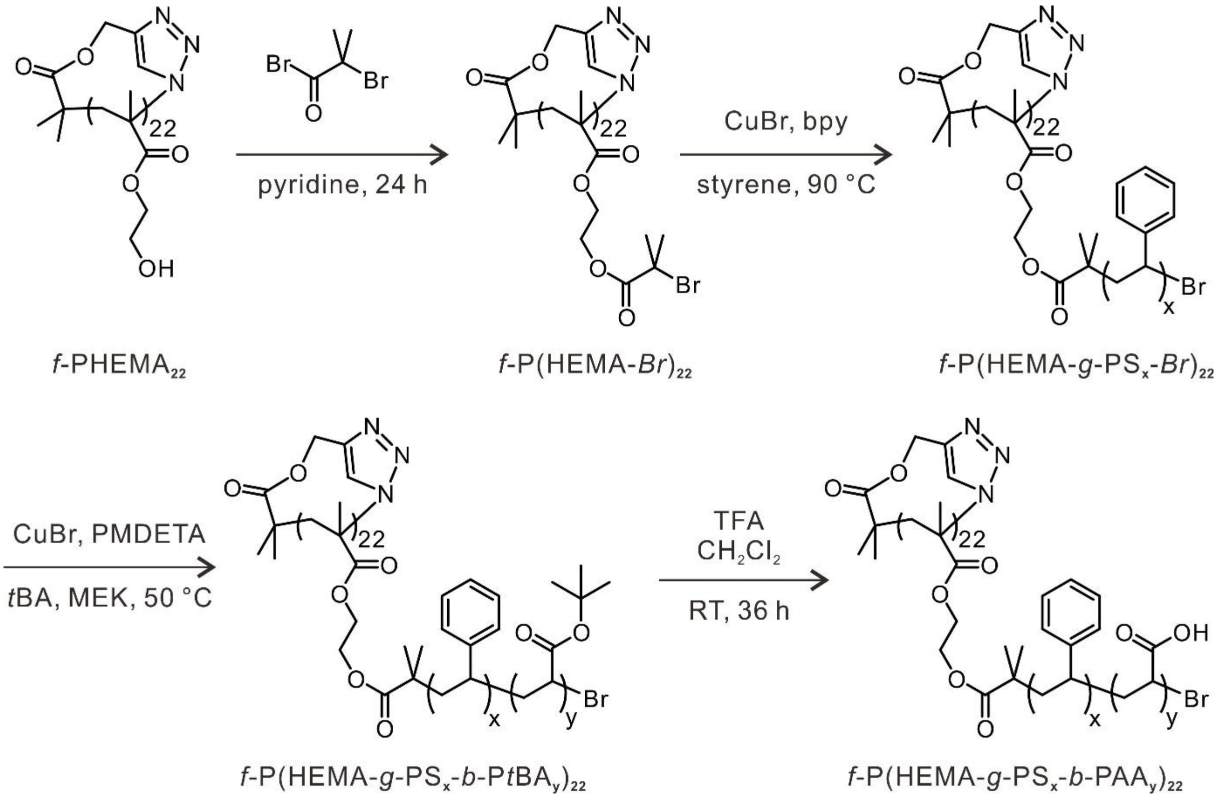

6.1 Synthesis of cyclic macroinitiator *f*-P(HEMA-*Br*)$_{22}$

Cyclic macroinitiator *f*-P(HEMA-*Br*)$_{22}$ was synthesized by esterification of *f*-PHEMA$_{22}$ with α-bromoisobutyryl bromide according to the literature[1]. Briefly, *f*-PHEMA$_{22}$ (80 mg, ~ 0.6 mmol −OH) was dissolved in 3 mL anhydrous pyridine in a 10 mL flask. After cooling to 0 °C in an ice-water bath, α-bromoisobutyryl bromide (0.5 mL, 3.85 mmol) was added dropwise during 20 min. The solution was further stirred at 0 °C for 1 h and then at room temperature for 24 h. The reaction mixture was precipitated in 50 mL cold water and separated by centrifugation. The collected product was dissolved in a small amount of THF and reprecipitated in water. After repeating the process for two cycles, the product was obtained after drying under high vacuum.

6.2 Synthesis of cyclic brush polymers *f*-P(HEMA-*g*-PS$_x$-*Br*)$_{22}$

In a typical procedure for the synthesis of cyclic brush polymer *f*-P(HEMA-*g*-PS$_{40}$-*Br*)$_{22}$, *f*-P(HEMA-*Br*)$_{22}$ (20 mg, 0.07 mmol initiation sites), styrene (5 mL, 43.6 mmol), and bpy (22.5 mg, 0.144 mmol) were added into a 15 mL Schlenk flask and the mixture was degassed through three freeze-pump-thaw cycles. CuBr (10.3 mg, 0.072 mmol) was then added under argon flow and the polymerization was proceeded at 90 °C. After reaction for a determined time, the polymerization was stopped after putting the flask into liquid nitrogen. THF was added and the reaction mixture was then passed through a short column filled with neutral Al_2O_3 to remove the copper catalyst. The crude product was precipitated in cold methanol. The procedure of

dissolution with THF and precipitation in methanol was repeated twice and the product was obtained after drying under high vacuum.

6.3 Synthesis of cyclic brush polymers *f*-P(HEMA-*g*-PS_x-*b*-P*t*BA_y)$_{22}$

In a typical procedure for the synthesis of cyclic brush polymer *f*-P(HEMA-*g*-PS_{40}-*b*-P*t*BA_y)$_{22}$, *f*-P(HEMA-*g*-PS_{40}-*Br*)$_{22}$ (100 mg), *t*BA (1.17 mL, 8 mmol), and PMDETA (14.3 µL, 0.064 mmol) were dissolved in 1.17 mL MEK in a Schlenk flask and the mixture was degassed through three freeze-pump-thaw cycles. CuBr (2.3 mg, 0.016 mmol) was then added under argon flow and the polymerization was proceeded at 50 °C. After reaction for 30 min, the polymerization was stopped by putting the flask into liquid nitrogen. Acetone was added and the reaction mixture was then passed through a short column filled with neutral Al_2O_3 to remove the copper catalyst. The crude product was precipitated in methanol/water (v/v=1/1) and used for the next hydrolysis step.

6.4 Synthesis of amphiphilic cyclic brush polymers *f*-P(HEMA-*g*-PS_x-*b*-PAA_y)$_{22}$

In a typical step, 60 mg of *f*-P(HEMA-*g*-PS_{40}-*b*-P*t*BA_y)$_{22}$ was dissolved in 8 mL CH_2Cl_2 followed by adding 4 mL TFA. The solution was stirred at room temperature for 36 h. After removing the solvent by evaporation, the product was dissolved in minimum amount of DMF and dialyzed against acetone and DI water. The final product was obtained after freeze drying.

7. Self-assembly of block copolymers, linear and cyclic brush polymers

A dialysis method was used to prepare assemblies of block copolymers, as well as linear and cyclic brush polymers with block side chains. For the preparation of self-assembly solutions at 0.4 mg mL^{-1}, 8 mg of polymer was dissolved in 5 mL of DMF at room temperature. The solution was vigorously stirred and 5 mL of deionized water was then added with a speed of 0.2 mL min^{-1}. After stirring for another 2 h, the solution was loaded to into a dialysis membrane (MWCO ~ 1000 Da for block copolymers, MWCO ~ 3500 Da for linear and cyclic brush polymers) and dialyzed against deionized water for three days to completely remove the solvent DMF. The final concentration of polymers was tuned to 0.4 mg mL^{-1} by adding water.

For the preparation of self-assembly solutions at 0.1 mg mL^{-1}, 4 mg of polymer was dissolved in 10 mL of DMF at room temperature. The solution was vigorously stirred and 10 mL of deionized water was then added with a speed of 0.2 mL min^{-1}. After stirring for another 2 h, the solution was loaded to into a dialysis membrane (MWCO ~ 1000 Da for block copolymers, MWCO ~ 3500 Da for linear and cyclic brush polymers) and dialyzed against deionized water for three days to completely remove the solvent DMF. The final concentration of polymers was tuned to 0.1 mg mL^{-1} by adding water.

8. Characterization

8.1 Nuclear magnetic resonance (NMR) spectroscopy

Nuclear magnetic resonance (NMR) spectra were measured with a 5 mm triple resonance TXI $^{1}H/^{13}C/^{15}N$ probe equipped with a z-gradient on a 300 MHz, 700 MHz, or 850 MHz Bruker AVANCE III system.

For a proton spectrum, 128 transients were used with a 9.5 µs long 90° pulse and a 17600 Hz spectral width together with a recycling delay of 5 s. The used carbon experiment was a J-modulated spin-echo for ^{13}C-nuclei coupled to ^{1}H to determine number of attached protons (definition up (positive): C and CH_2, down (negative): CH and CH_3) with decoupling during acquisition.

The temperature was regulated at 298.3 K and calibrated with a standard ^{1}H methanol NMR sample using the Topspin 3.1 software (Bruker). The control of the temperature was realized with a VTU (variable temperature unit) and an accuracy of +/− 0.1K.

The structure prove was realized by 2D $^{1}H,^{1}H$ correlation spectroscopy (COSY) and 2D $^{1}H,^{1}H$ nuclear Overhauser effect spectroscopy (NOESY) methods. The spectroscopic widths of the homonuclear 2D-COSY and 2D-NOESY experiments were typically 14500 Hz in both dimension (f1 and f2) and the relaxation delay 2 s. The chosen mixing time for the NOESY experiment was 300 ms.

All the 2D $^{1}H,^{13}C$-HSQC-edited (heteronuclear single quantum correlations via double inept transfer and phase sensitive using Echo/Antiecho-TPPI gradient selection with decoupling during acquisition, red cross peaks are CH or CH_3 and black cross peaks are CH_2) experiments run with 2048 points in f2 (8500 Hz) and 512 points in f1 (42700 Hz) dimension. Before Fourier transformation, the data were zero filled to 1024 points in f1 and multiplied by a window function (q-sine bell or sine bell) in both dimensions.

Diffusion ordered NMR spectroscopy (DOSY NMR) experiments were performed with a gradient strength of 5,350 [G/mm] on a Bruker Avance-III 850 NMR Spectrometer. The gradient strength of probes was calibrated by analysis of a sample of $^{2}H_2O/^{1}H_2O$ at a defined temperature and compared with the theoretical diffusion coefficient of $^{2}H_2O/^{1}H_2O$ (values taken from Bruker diffusion manual) at 298.3 K.

In this work, the diffusion time (d20) was optimized for the TXI probe to 60ms while the gradient pulse length was kept at 1.8 ms. The optimization was realized by comparing the remaining intensity of the signals at 2% and 95% gradient strength. The intensity loss of the echo was in the range of 90 %.

The diffusion measurements were done with a 2D DOSY sequence[2] by incrementing in 32 linear steps from 2% to 100% with the TXI and the QXI probe. The 2D NMR sequences for measuring diffusion coefficient used echoes for convection compensation and longitudinal eddy current delays to store the magnetization in the z-axis, and only be dependent on T_1-relaxation. The calculation of the diffusion value was automatically calculated with the mono exponential function[3]:

$$\ln\left(\frac{I(G)}{I(0)}\right) =- \gamma^2\delta^2G^2\left(\Delta - \frac{\delta}{3}\right)D,$$

where I(G) and I(0) are the intensities of the signals with and without gradient, γ the gyromagnetic ratio of the nucleus (^{1}H in this measurements), G is the gradient strength, δ the duration of the pulse field gradient (PFG), D the diffusion value in m^2/s and Δ the "diffusion time" between the beginning of the two gradient pulses. The relaxation delay between the scans was 3 s.

The 2D sequence for diffusion measurement used double stimulated echo with three spoil gradients for convection compensation and with an eddy current delay of 5 ms for reduction[4] (acronym Bruker pulse program: dstebpgp3s).

8.2 Fourier-transform infrared (FTIR) spectroscopy

Fourier-transform infrared (FTIR) spectroscopy spectra were recorded on a Bruker TENSOR II spectrometer or a Bruker Vertex 70 spectrometer at room temperature in the range of 4000 to 400 cm^{-1}. For the former, the samples were measured directly. For the latter instrument, the solid samples were thoroughly mixed with KBr and pressed into a flaky form. The spectra were collected over 32 scans with a spectral resolution of 4 cm^{-1}.

8.3 Gel permeation chromatography (GPC)

GPC was used to determine the molecular weight and molecular weight distribution. The temperature was set at 60 °C. DMF was used as eluent and the flow rate was set as 1 mL min^{-1}. Poly(methyl methacrylate) (PMMA) or polystyrene (PS) standards were used for calibration and refractive index (RI) detector was used.

8.4 Dynamic light scattering (DLS)

Folded PHEMA homopolymers were diluted to a concentration of 1 mg/mL in Milli-Q water and filtered through a 450 nm filter (Millipore HA). DLS was performed on an ALV5000 setup using a coherent solid state cw laser at λ = 633 nm with a power of 194 mW. The intensity autocorrelation function $G(q,t)$ was recorded at different scattering wave vectors, $q = \frac{4\pi n}{\lambda}\sin\frac{\theta}{2}$ with n being the solvent refractive index was varied by changing the scattering angle θ between 15° ~ 150°, for water at 293 K, n = 1.333. The desired relaxation function, $C(q,t)=[G(q,t)-1]^{1/2}$, was analyzed by an inverse Laplace transformation (CONTIN algorithm).

8.5 Transmission electron microscopy (TEM)

TEM samples were prepared by adding 4 μL of the polymer self-assembly solution onto a carbon-coated copper grid. After drying in air for 10 min, the remained solution was removed by a filter paper. If staining is required, 10 μL of 2% uranyl acetate solution was first dropped on a parafilm to form a droplet. The sample was then stained for 45 seconds by inverting the TEM grid (let the sample side down) on the droplet. After removing the staining solution with a filter

paper, the sample was washed with Milli-Q water for three times (shaken in water for 6 seconds for each time). After drying in air, the measurement was conducted on a JEOL JEM-1400 TEM operating at an accelerating voltage of 120 kV.
For cryo-TEM examination the samples were vitrified using a Vitrobot Mark V (Thermo Fisher) plunging device. 5 μl of the sample dispersion was applied to a Quantifoil or a holey carbon coated TEM grid that has been glow discharged shortly before. After removing excess sample solution with a filter paper the grid is immediately plunged into liquid ethane. For the subsequent examination the specimens were transferred to a TEM (Thermo Fisher Krios G4) and examined at cryogenic temperature. Images were acquired using a 4k Ceta Camera.

8.6 Atomic force microscopy (AFM)

Imaging was performed with a Bruker Dimension FastScan Bio AFM equipped with the ScanAsyst mode. The sample solution was deposited onto freshly cleaved mica surface, and left for 5 min at room temperature. The sample was scanned with the scan rates between 1 and 3 Hz. Several AFM images were acquired at different areas of the mica surface to ensure the reproducibility of the results. All images were analyzed by using the Gwyddion 2.48 software.

9. All-atom molecular dynamics simulations

To investigate the structural stability of an isolated cyclic molecule in pure water, we have performed all-atom molecular dynamics simulation of a *f*-$PHEMA_{15}$ (Fig. 1e). For this purpose, we have the same structure as in the experiment, without the linker used in the synthesis (Supplementary Fig. 24).

All-atom simulations are performed using the GROMACS molecular dynamics package[5] in an isobaric ensemble (NPT), where N is the number of particles, P is the isotropic pressure, and T is the temperature. T = 300 K is set using a velocity rescaling thermostat with a coupling constant of 0.1 ps[6]. Pressure is kept at 1 bar using the Parrinello-Rahman barostat with a coupling constant of 2 ps[7]. Electrostatics are treated with the particle mesh ewald (PME) method[8]. The interaction cutoff for the non-bonded interactions is chosen as 1.0 nm and the equations of motions are integrated using the leap-frog integrator with a time step of δt = 1 fs. The simulation is performed for 20 ns.

For the simulation of a PHEMA ring with n = 15, the united atom GROMOS force field[9] is used. Water is described using the SPC/E model[10]. For this simulation, we have used one molecule solvated in 7000 water molecules.

10. Generic molecular dynamics simulations

Generic simulations are performed using the bead spring polymer model for both systems presented in the main text[11]. In this model, individual bonded monomers interact with each other via a combination of 6−12 Lennard-Jones potential with a cutoff distance $2^{1/6}\sigma$ and a finitely

extensible nonlinear elastic (FENE) potential. The results are presented in the unit of LJ energy ε, LJ distance σ and mass *m* of the individual monomers.

The nonbonded interactions between the both hydrophobic and hydrophilic residues are also modelled using the LJ potential. The simulation is performed under the canonical ensemble with a time step of $\Delta t = 0.01\tau$ for 10^8 MD time steps. The equations of motion are integrated using the velocity Verlet algorithm. The system is thermalized via a Langevin thermostat with a damping constant $\gamma = 1\tau^{-1}$ and $T = 1\varepsilon/k_B$, where k_B is the Boltzmann constant. The generic simulations are performed by using the LAMMPS molecular dynamics packages[12].

10.1 Molecular dynamics simulations for the self-assembly of folded PHEMA

For the simulation of folded PHEMA, we have used a model molecule that consists of a hydrophobic backbone and one hydrophilic molecule attached to each backbone monomer. A representative structure is shown in Fig. 1a and Supplementary Fig. 24. The hydrophobic monomers interact with an attractive 6-12 LJ potential with a cut-off of 2.5σ, while the hydrophilic monomers are modelled using a repulsive 6-12 LJ potential with a cut-off of $2^{1/6}\sigma$. The size of all monomers is taken as 1σ and the interaction energy are taken as 1ε. All other cross-monomer interactions are chosen to be repulsive 6-12 LJ potential with a cut-off of $2^{1/6}\sigma$. For these simulations, we have chosen three different backbone lengths, namely n = 9 and 15. Here, the concentration of n = 9 system is taken as $\rho = 0.0002\sigma^{-3}$, with c is calculated as the number of molecules per unit volume. The concentrations are adjusted such that the total mass density (or monomer number density). To keep the backbone structure flat, we have employed an additional dihedral interaction. Note that in this generic model one monomer is not equivalent of one monomer of the all atom or the experimental system.

10.2 Molecular dynamics simulations for the self-assembly of cyclic brush polymers

The system of cyclic brush polymers is similar to the above PHEMA system with a short amphiphilic block copolymer attached to each hydrophilic side monomer (Fig. 3a). An amphiphilic side chain consists of a 3 hydrophobic and 2 hydrophilic monomers. Like the section 2.1, the hydrophilic and the hydrophobic interactions are again modelled using a repulsive and an attractive LJ interaction, respectively. The simulations are performed for a system consists with n = 9 and for the five different concentrations, $\rho = 0.0002\sigma^{-3}$, $0.00035\sigma^{-3}$, $0.0005\sigma^{-3}$, $0.0009\sigma^{-3}$, and $0.0017\sigma^{-3}$. For these simulations we have used 125 molecules in a cubic box of different box dimensions. Each amphiphilic side chain consists of a flexible generic chain[11].

Supplementary Figures

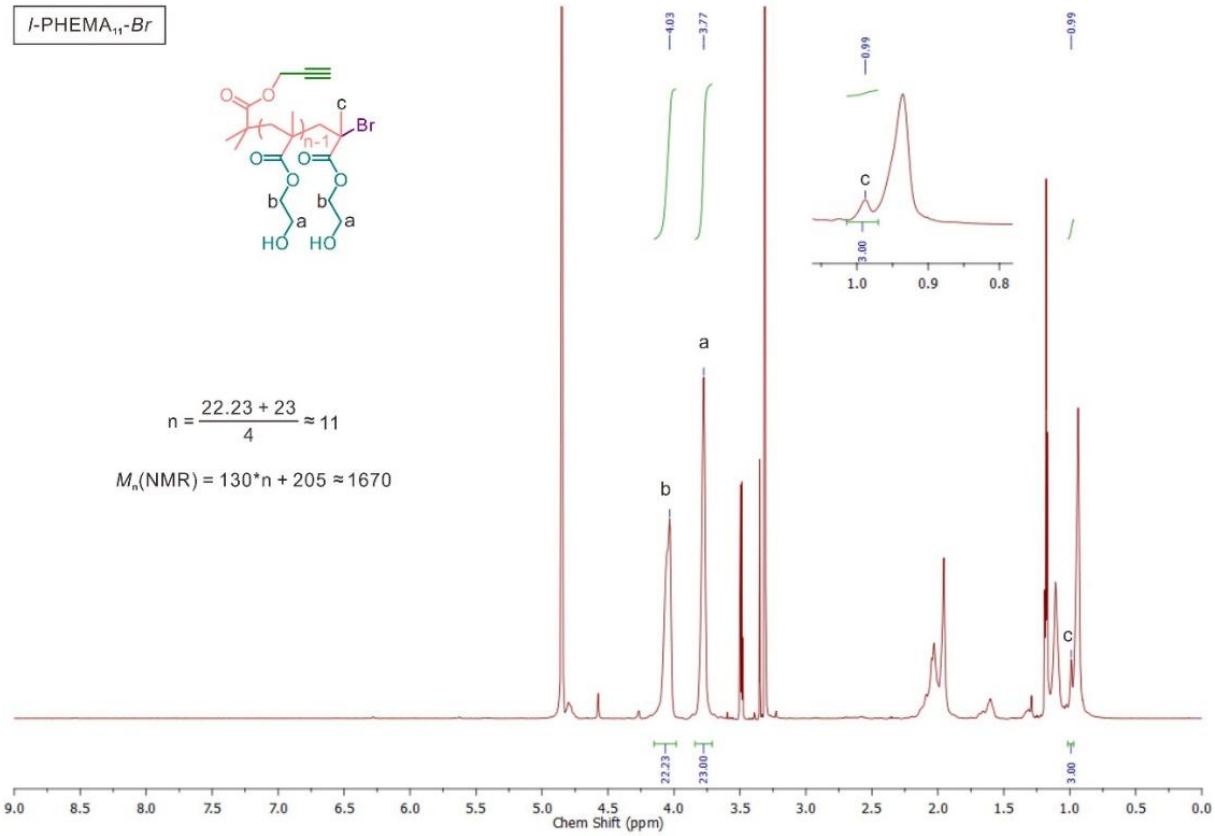

Supplementary Fig. 1. ^{1}H NMR spectrum (850 MHz, 298.3 K) of *l*-PHEMA$_{11}$-*Br* in methanol-d$_4$ (MeOD). By comparing integrals of the methyl group near the bromine end (0.99 ppm) to methylene groups (3.77 ppm and 4.03 ppm) in the side chains, the average molecular weight was calculated as 1670 g mol^{-1} (n = 11).

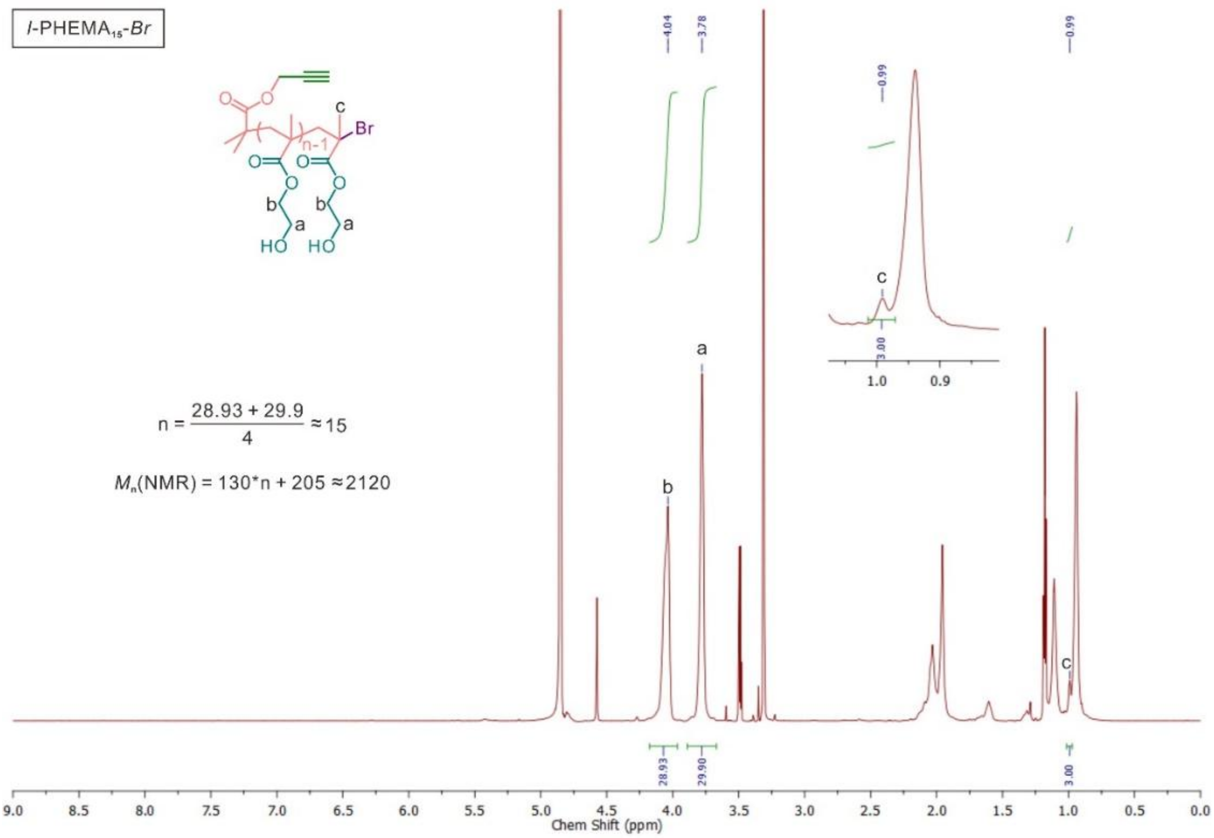

Supplementary Fig. 2. ^{1}H NMR spectrum (850 MHz, 298.3 K) of *l*-PHEMA$_{15}$-*Br* in MeOD. By comparing integrals of the methyl group near the bromine end (0.99 ppm) to methylene groups (3.78 ppm and 4.03 ppm) in the side chains, the average molecular weight was calculated as 2120 g mol^{-1} (n = 15).

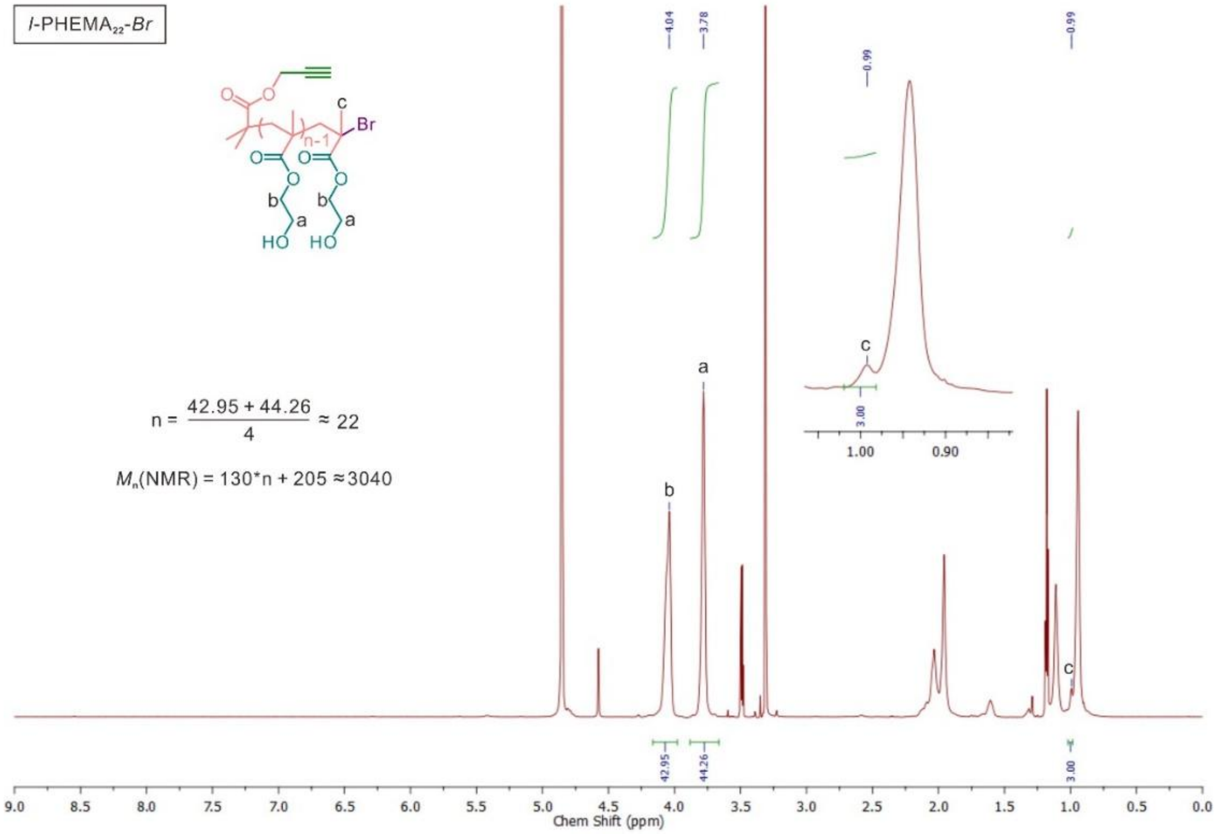

Supplementary Fig. 3. ^{1}H NMR spectrum (850 MHz, 298.3 K) of *l*-PHEMA$_{22}$-*Br* in MeOD. By comparing integrals of the methyl group near the bromine end (0.99 ppm) to methylene groups (3.78 ppm and 4.03 ppm) in the side chains, the average molecular weight was calculated as 3040 g mol^{-1} (n = 22).

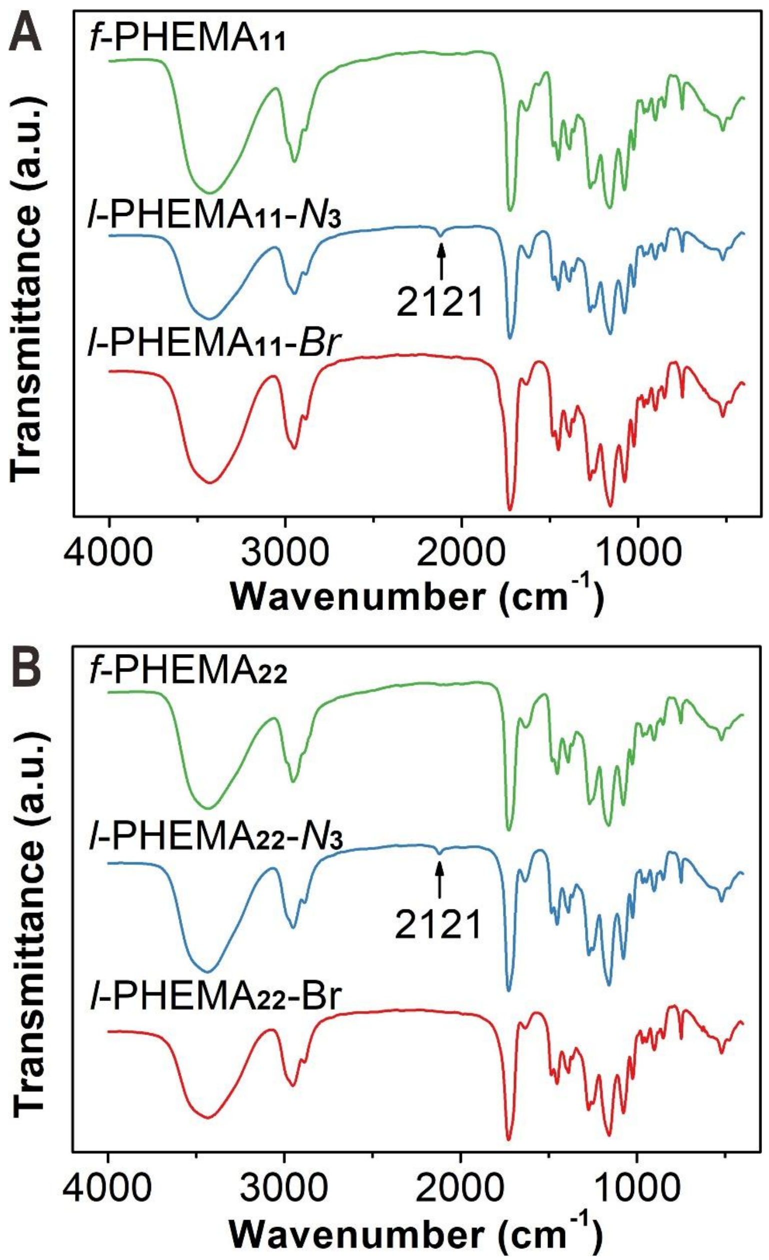

Supplementary Fig. 4. (A) FTIR spectra of *l*-$PHEMA_{11}$-*Br*, *l*-$PHEMA_{11}$-N_3, and *f*-$PHEMA_{11}$. (B) FTIR spectra of *l*-$PHEMA_{22}$-*Br*, *l*-$PHEMA_{22}$-N_3, and *f*-$PHEMA_{22}$.

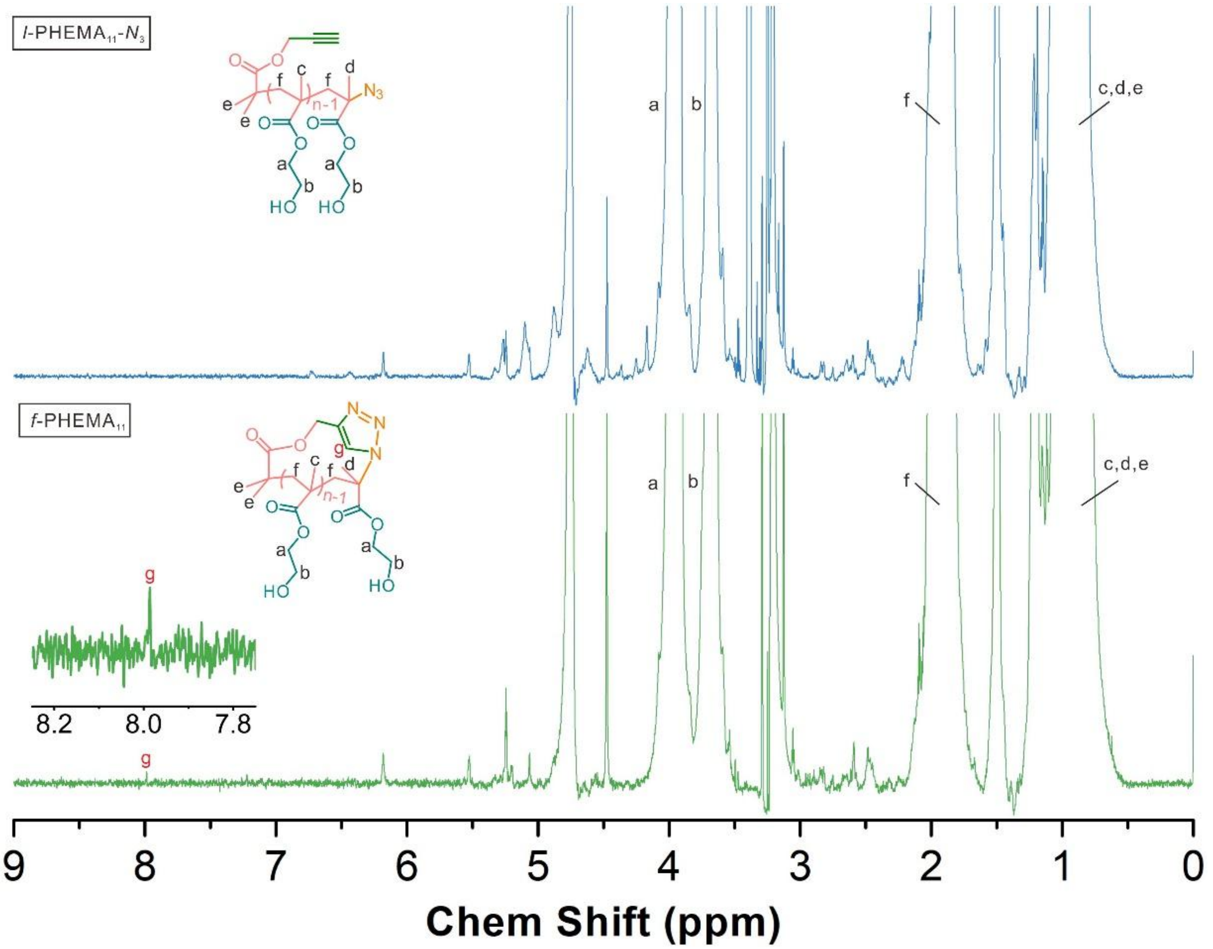

Supplementary Fig. 5. Comparison of ^{1}H NMR spectra (850 MHz, MeOD, 298.3 K) of *l*-PHEMA$_{11}$-*N*$_{3}$ and *f*-PHEMA$_{11}$.

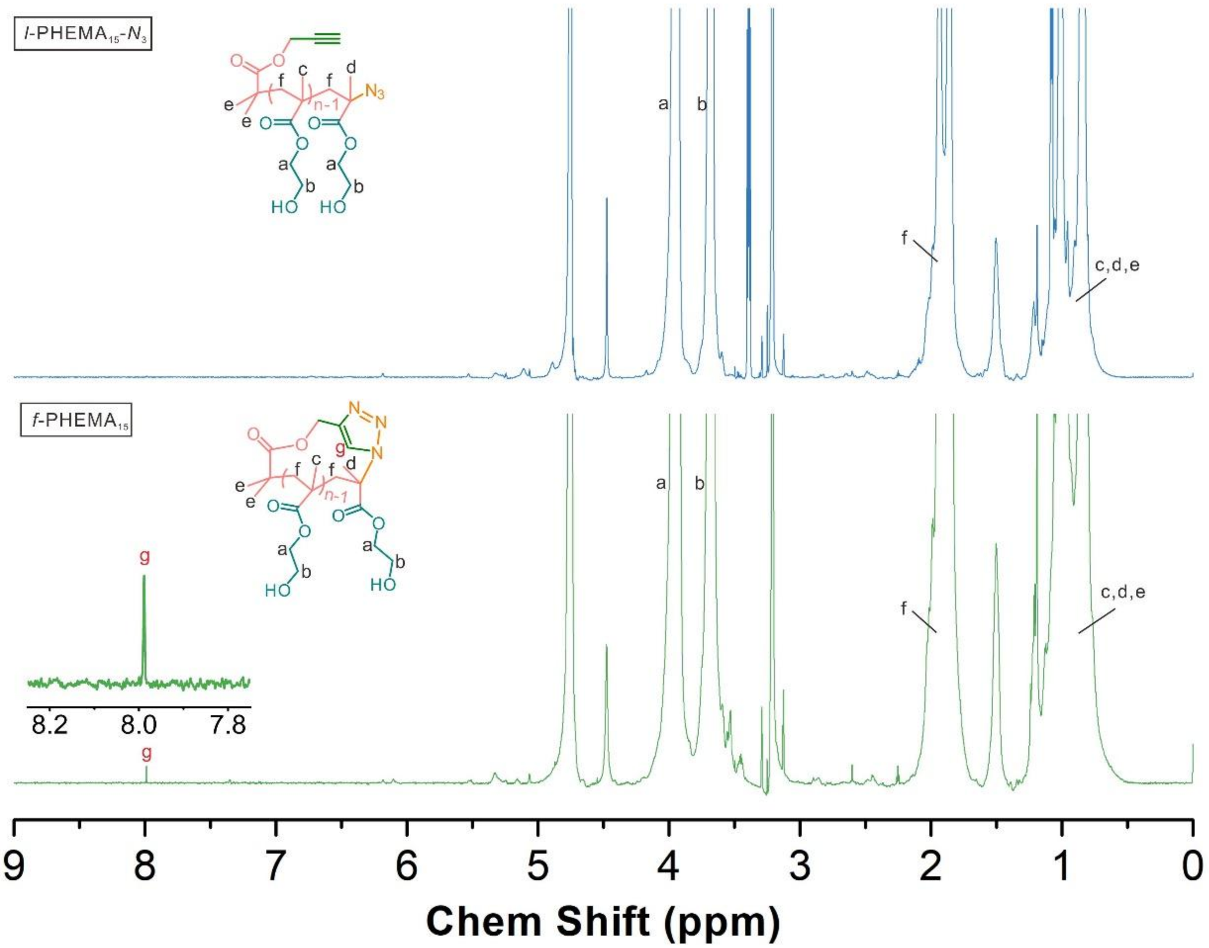

Supplementary Fig. 6. Comparison of ^{1}H NMR spectra (850 MHz, MeOD, 298.3 K) of *l*-PHEMA$_{15}$-*N*$_3$ and *f*-PHEMA$_{15}$.

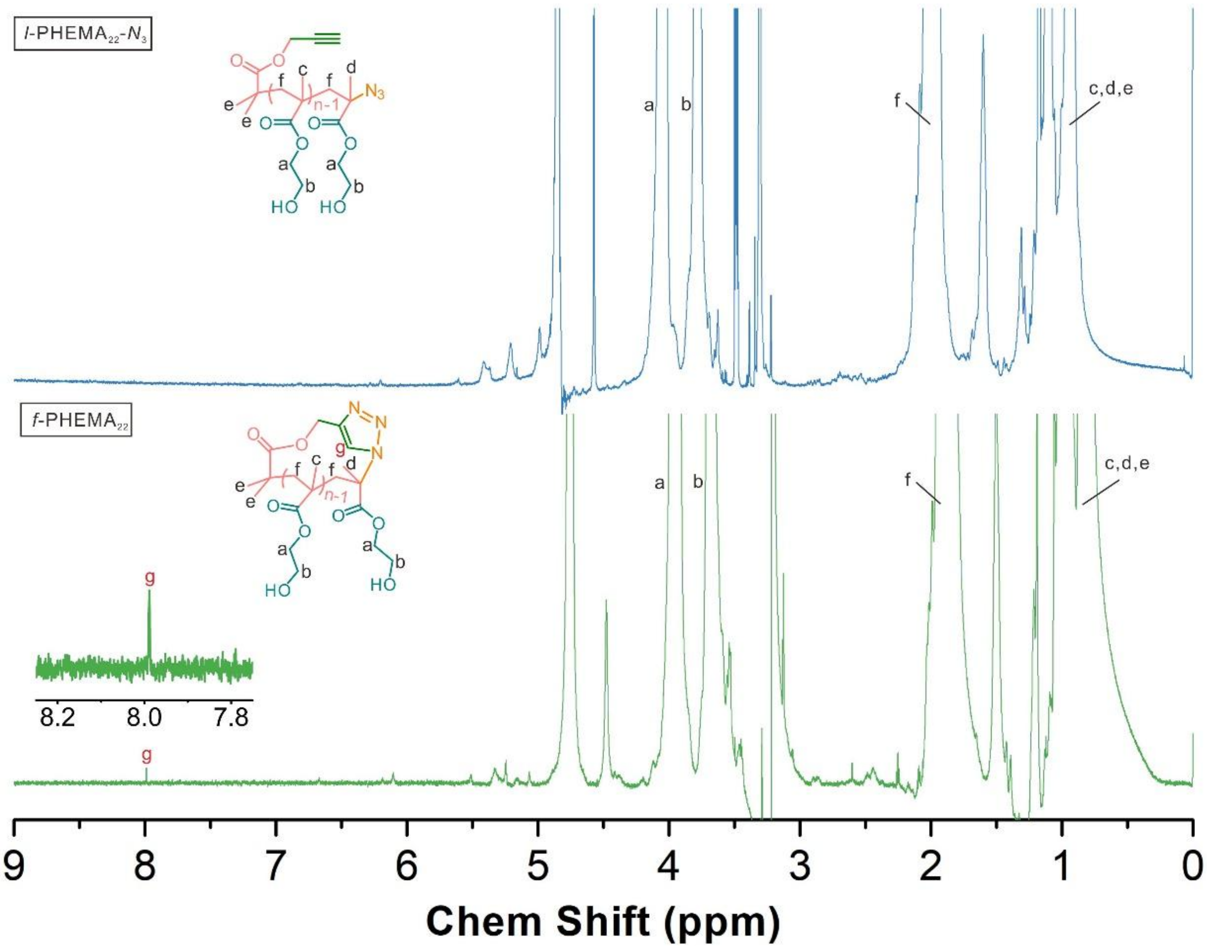

Supplementary Fig. 7. Comparison of ^{1}H NMR spectra (850 MHz, MeOD, 298.3 K) of *l*-PHEMA$_{22}$-*N*$_{3}$ and *f*-PHEMA$_{22}$.

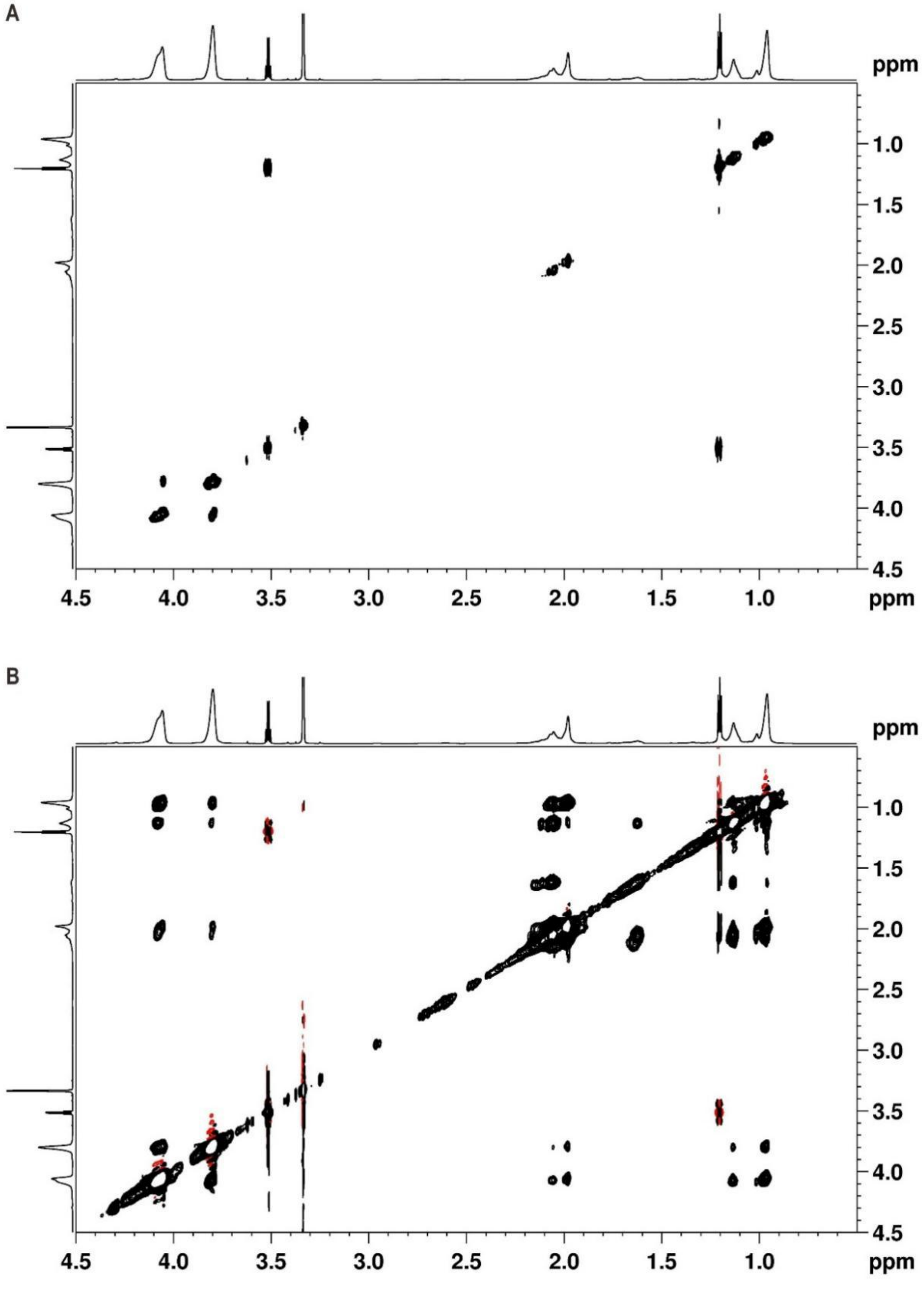

Supplementary Fig. 8. 2D $^{1}H,^{1}H$ COSY and 2D $^{1}H,^{1}H$ NOESY NMR spectra (850 MHz, 298.3 K) of *l*-$PHEMA_{11}$-*Br* in MeOD.

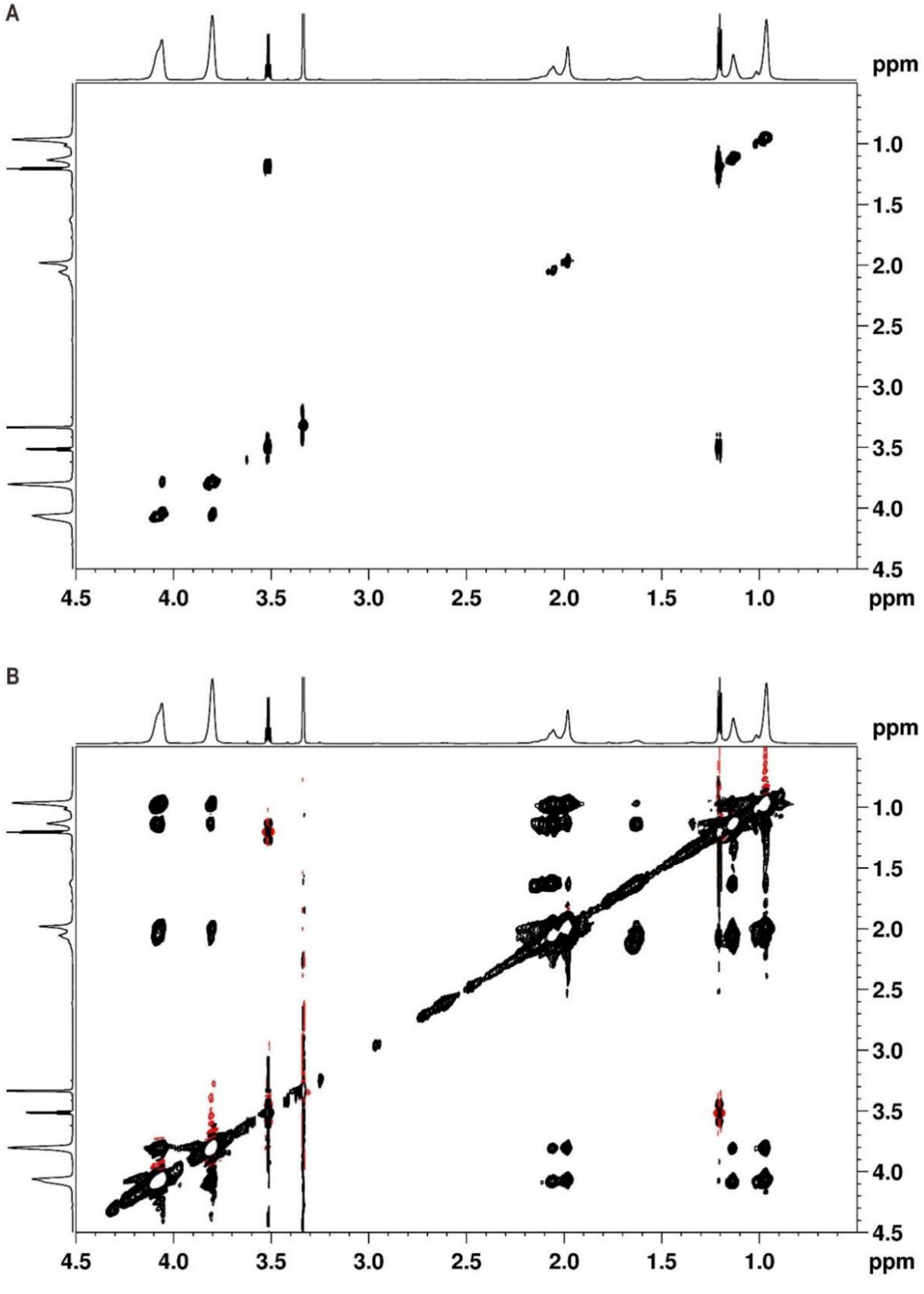

Supplementary Fig. 9. 2D $^{1}H,^{1}H$ COSY and 2D $^{1}H,^{1}H$ NOESY NMR spectra (850 MHz, 298.3 K) of *l*-PHEMA$_{15}$-*Br* in MeOD.

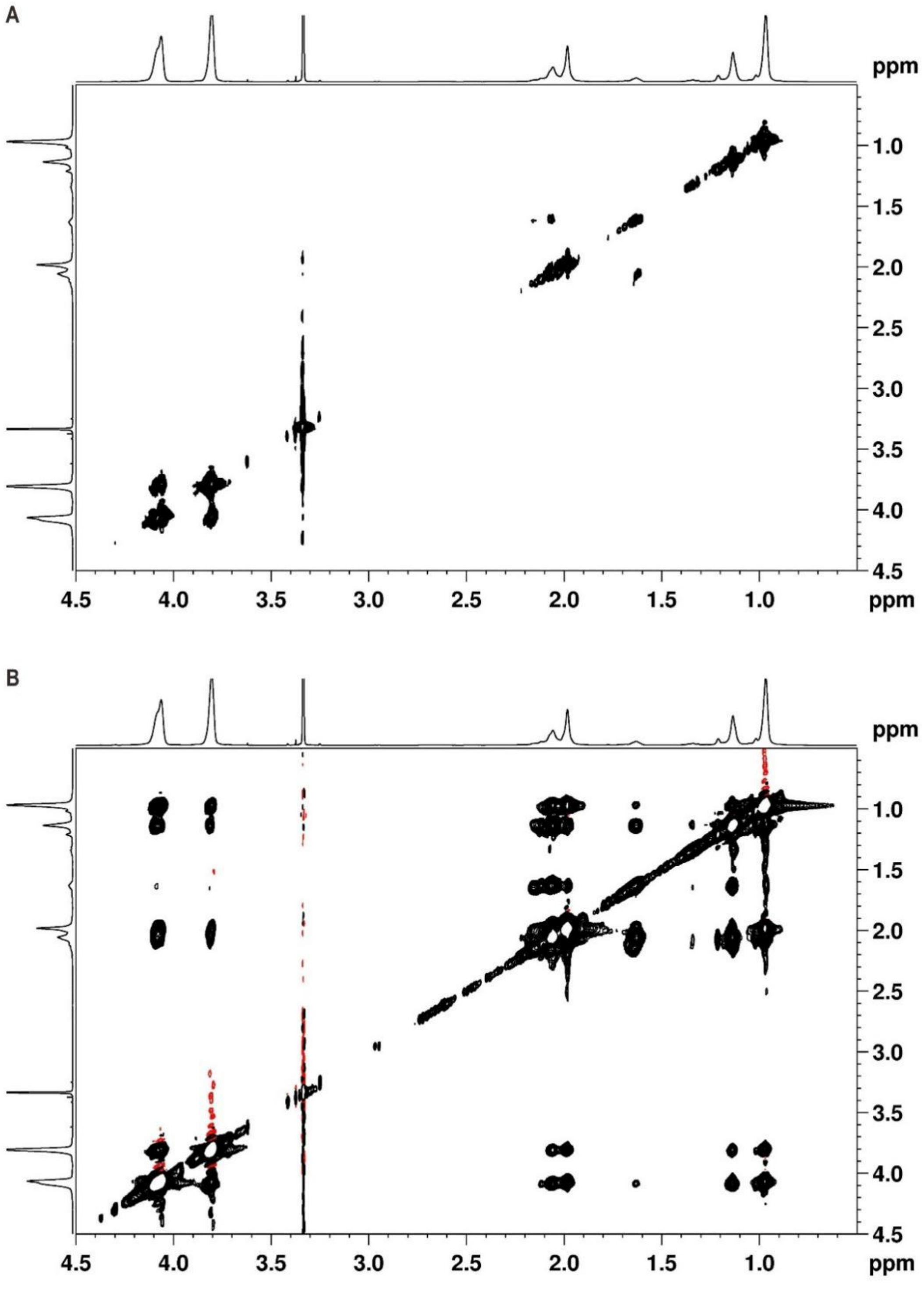

Supplementary Fig. 10. 2D ^{1}H,^{1}H COSY and 2D ^{1}H,^{1}H NOESY NMR spectra (850 MHz, 298.3 K) of *l*-PHEMA$_{22}$-*Br* in MeOD.

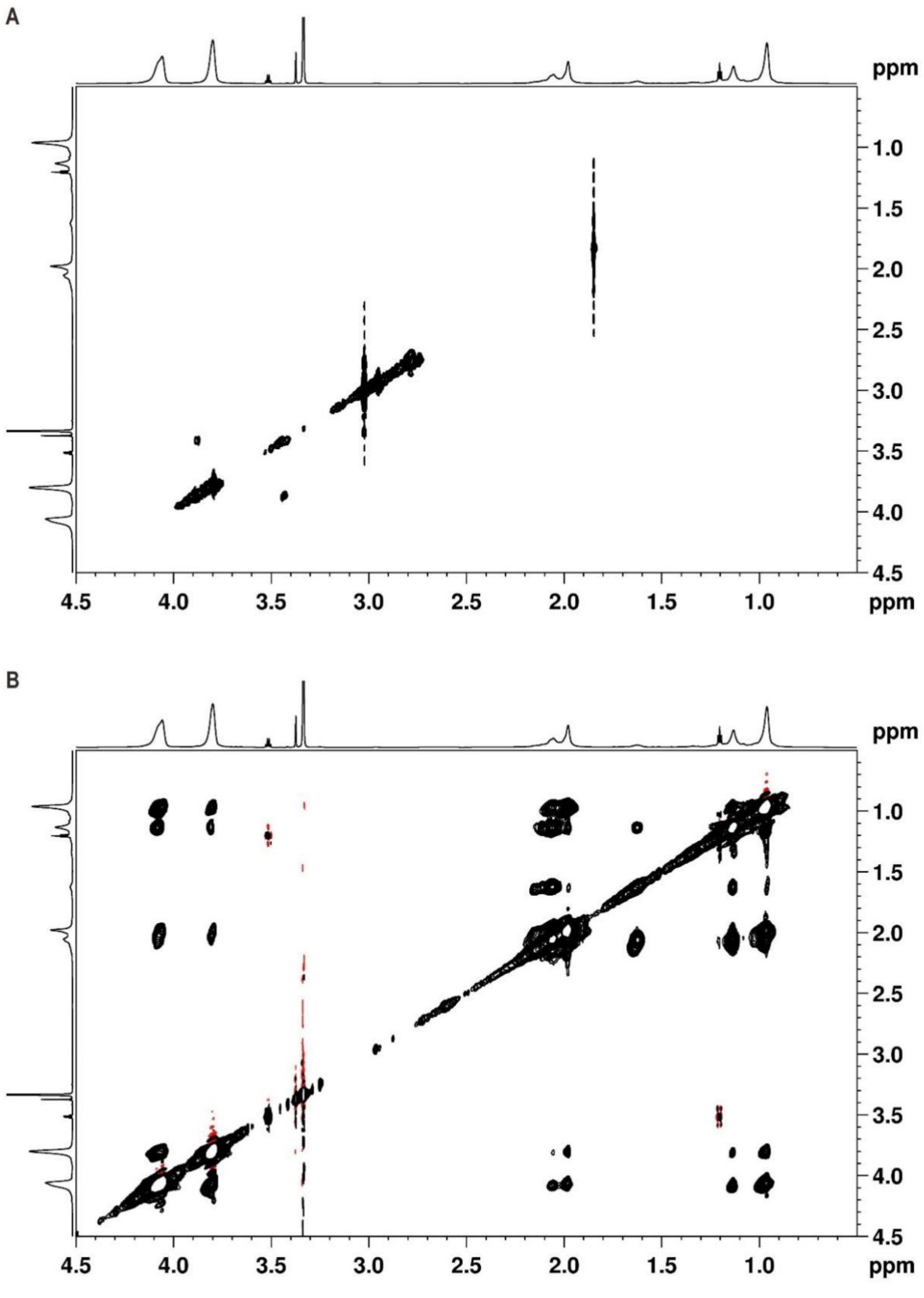

Supplementary Fig. 11. 2D $^1H,^1H$ COSY and 2D $^1H,^1H$ NOESY NMR spectra (850 MHz, 298.3 K) of *l*-PHEMA$_{11}$-N_3 in MeOD.

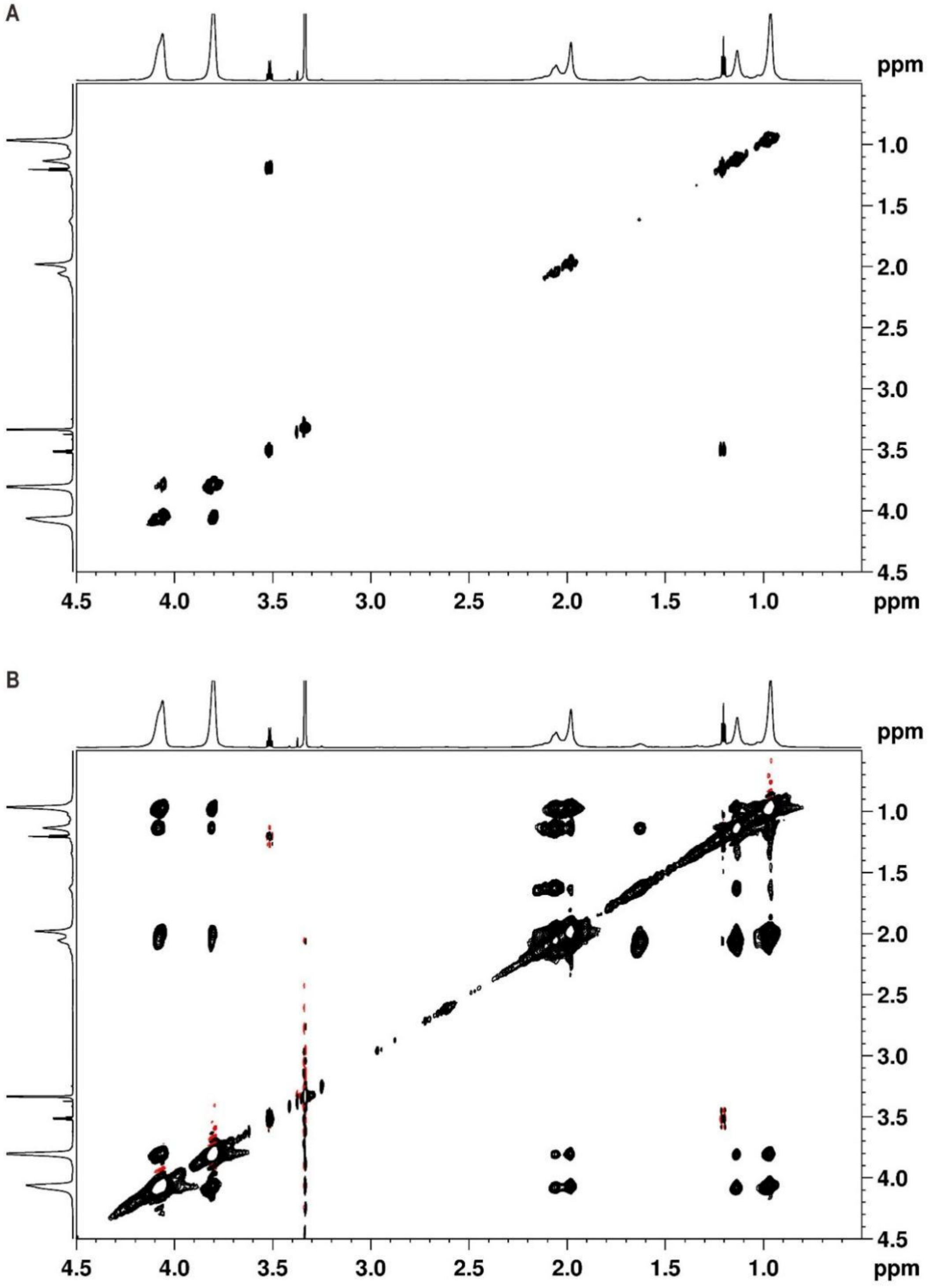

Supplementary Fig. 12. 2D ^{1}H,^{1}H COSY and 2D ^{1}H,^{1}H NOESY NMR spectra (850 MHz, 298.3 K) of *l*-PHEMA$_{15}$-*N*$_3$ in MeOD.

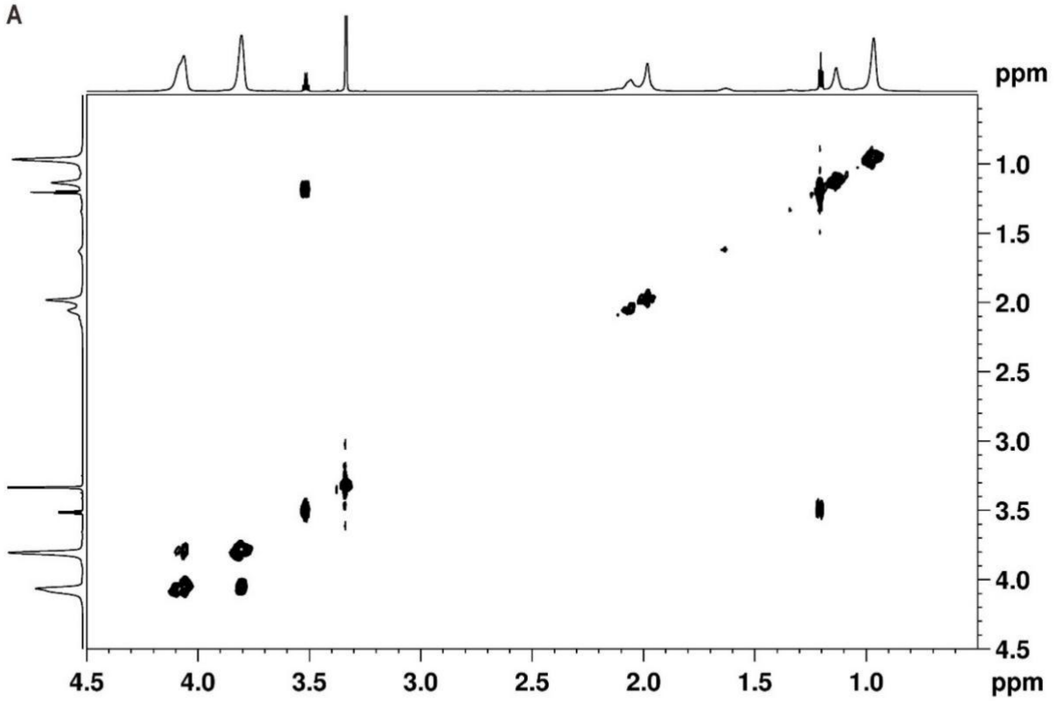

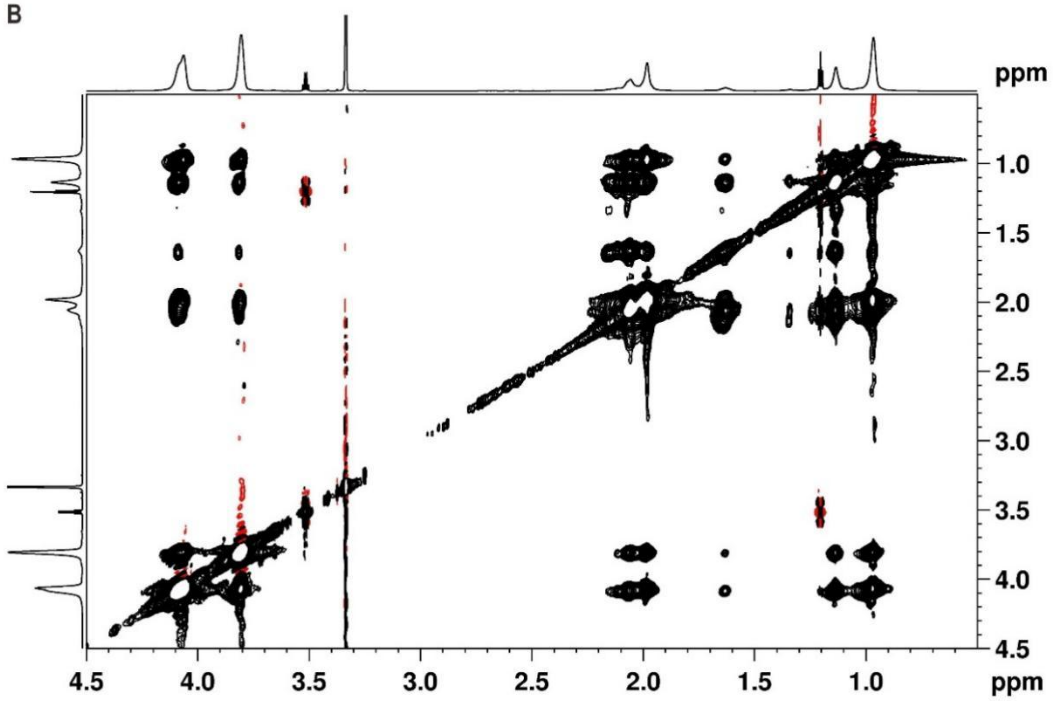

Supplementary Fig. 13. 2D ^{1}H,^{1}H COSY and 2D ^{1}H,^{1}H NOESY NMR spectra (850 MHz, 298.3 K) of *l*-PHEMA$_{22}$-N_3 in MeOD.

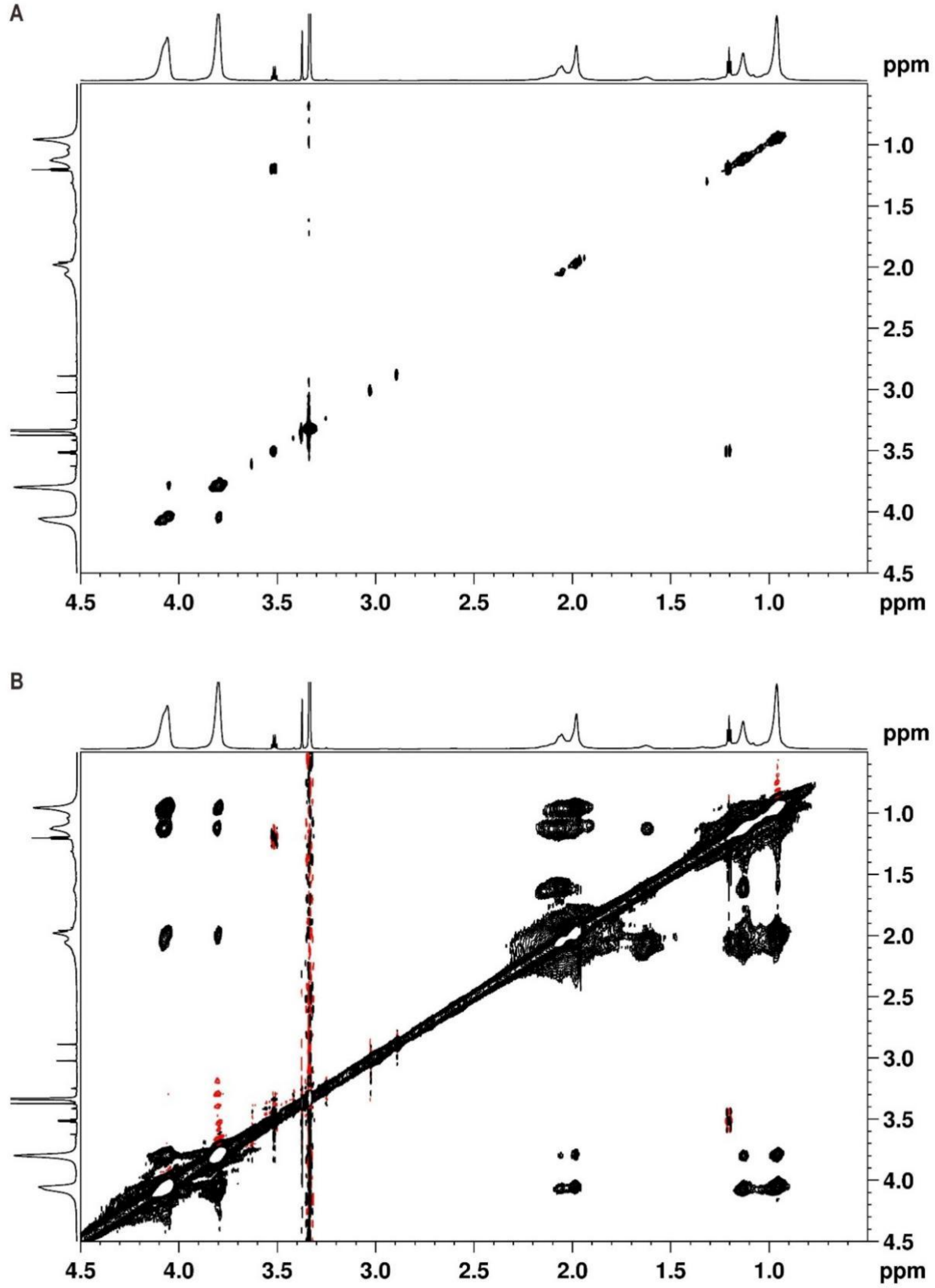

Supplementary Fig. 14. 2D ^{1}H,^{1}H COSY and 2D ^{1}H,^{1}H NOESY NMR spectra (850 MHz, 298.3 K) of *f*-PHEMA$_{11}$ in MeOD.

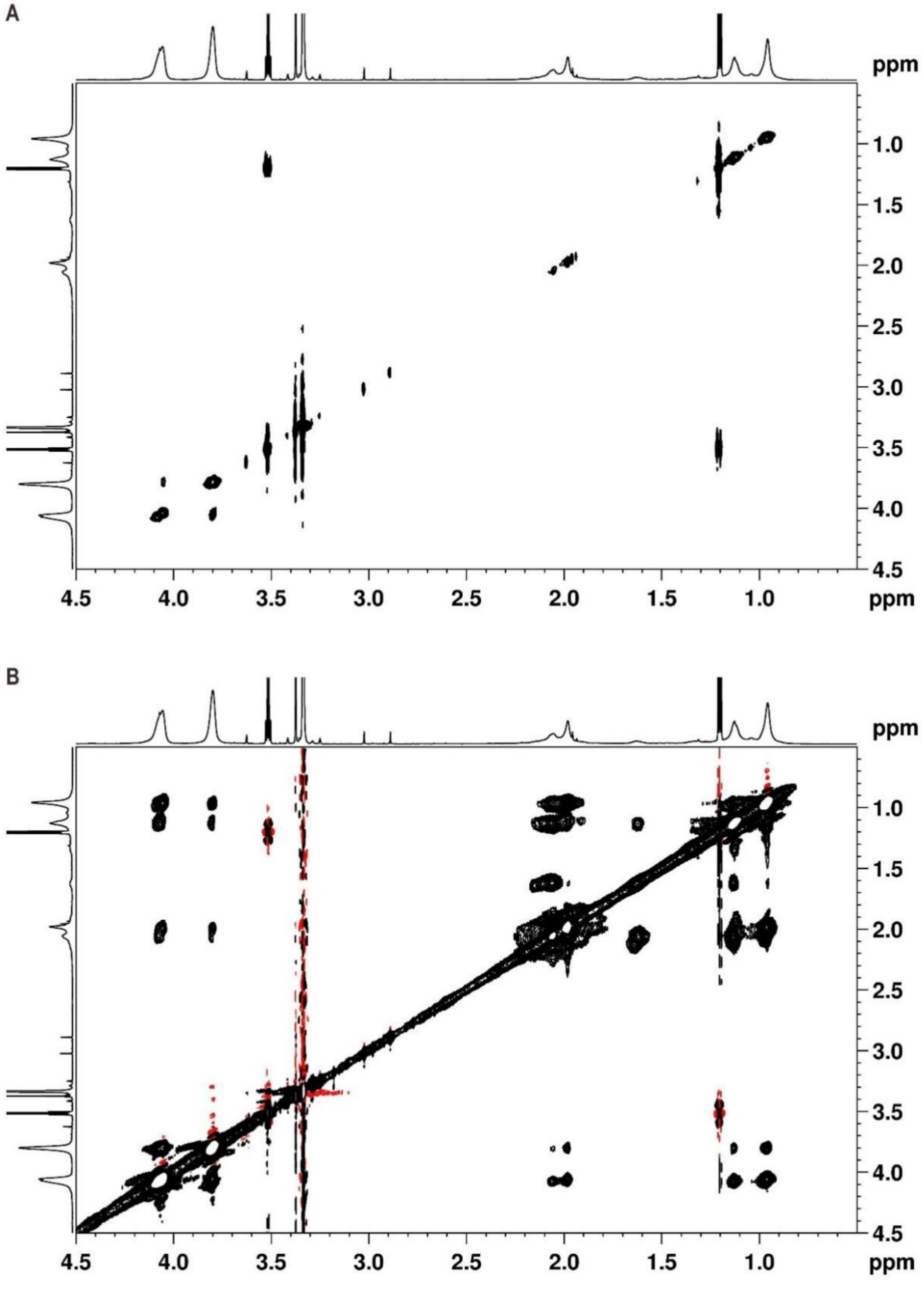

Supplementary Fig. 15. 2D ^{1}H,^{1}H COSY and 2D ^{1}H,^{1}H NOESY NMR spectra (850 MHz, 298.3 K) of *f*-PHEMA$_{15}$ in MeOD.

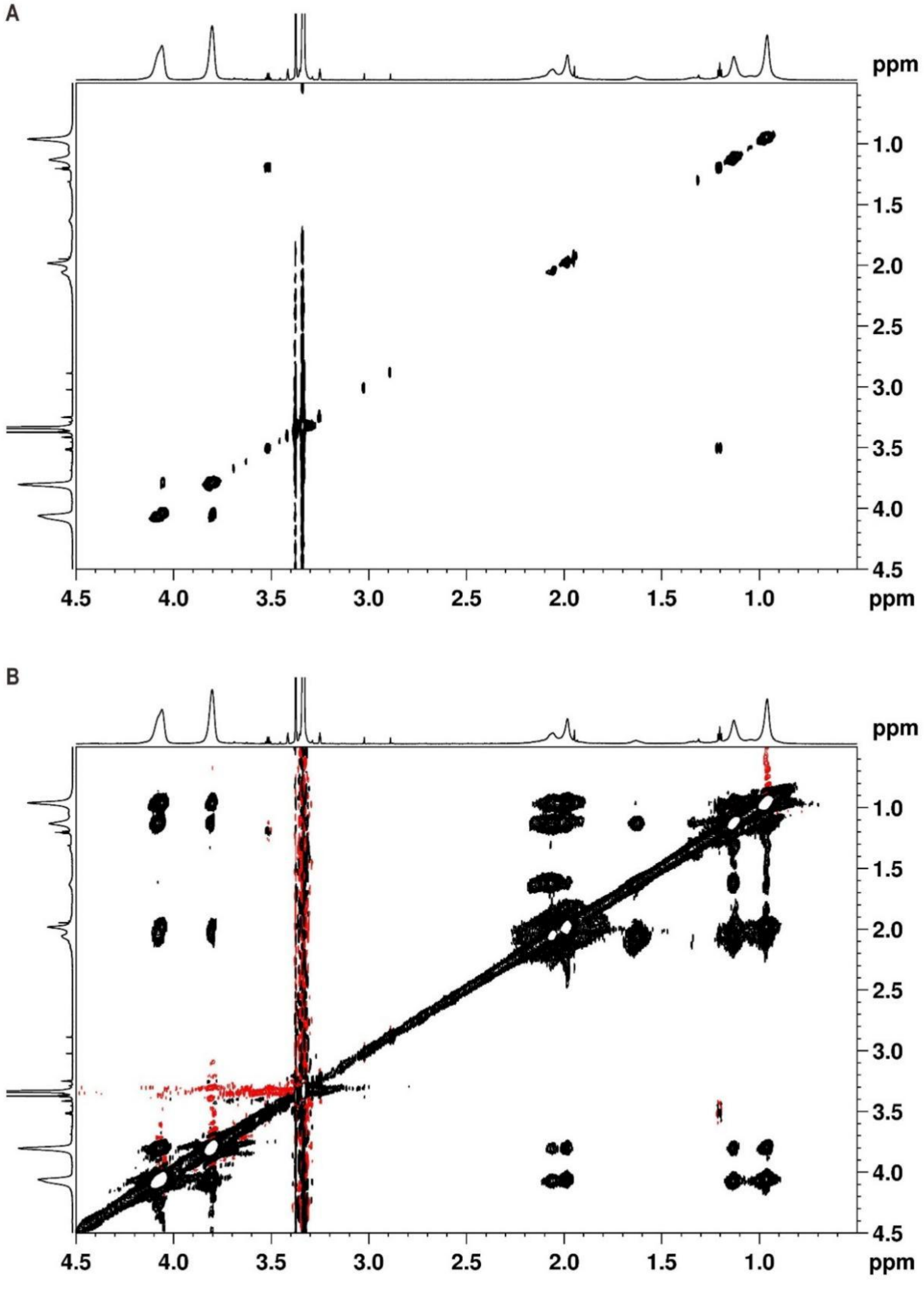

Supplementary Fig. 16. 2D ^{1}H,^{1}H COSY and 2D ^{1}H,^{1}H NOESY NMR spectra (850 MHz, 298.3 K) of *f*-$PHEMA_{22}$ in MeOD.

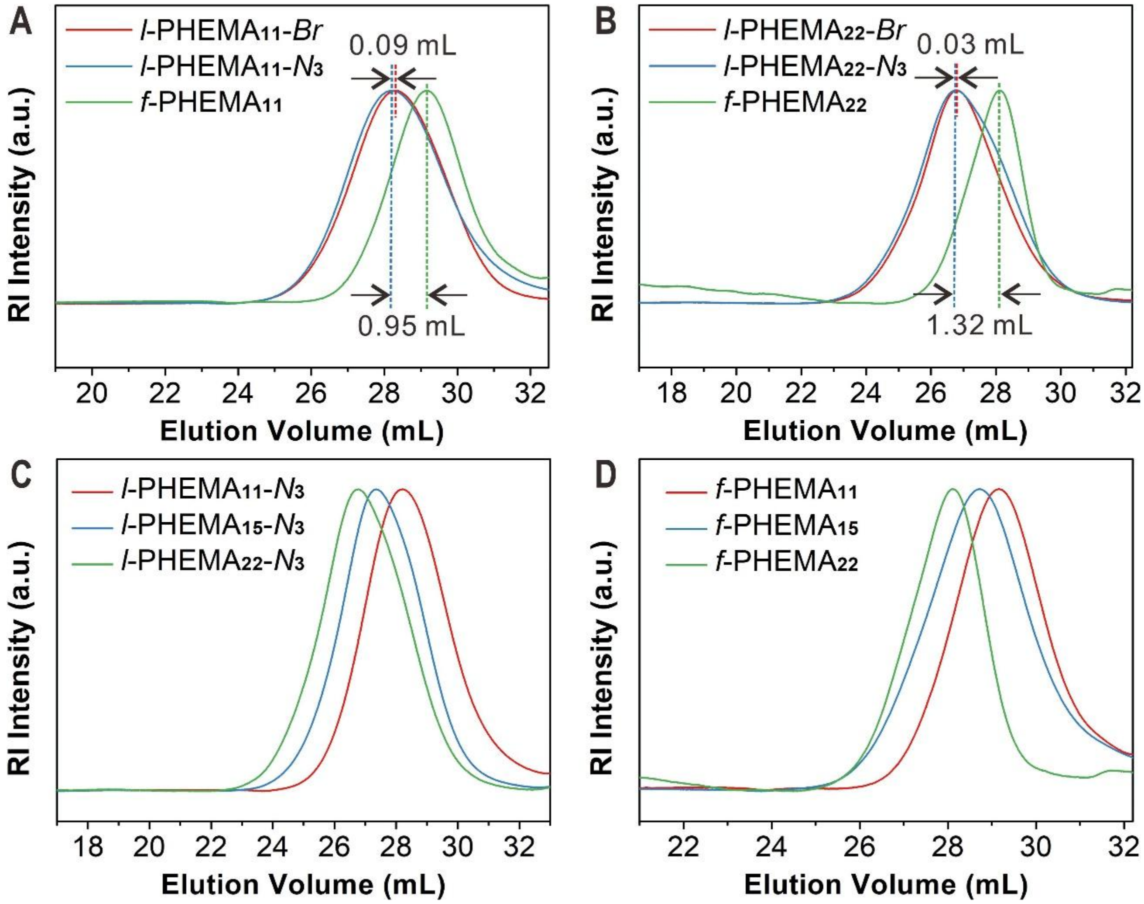

Supplementary Fig. 17. (A) GPC elution curves of *l*-PHEMA$_{11}$-*Br*, *l*-PHEMA$_{11}$-*N*$_3$, and *f*-PHEMA$_{11}$. (B) GPC curves of *l*-PHEMA$_{22}$-*Br*, *l*-PHEMA$_{22}$-*N*$_3$, and *f*-PHEMA$_{22}$. (C) Comparison of the GPC elution curves of *l*-PHEMA$_n$-*N*$_3$ with different repeating units. (D) Comparison of the GPC elution curves of folded polymers *f*-PHEMA$_n$ with different ring sizes. DMF was used as the eluent and PMMA standards were employed for calibration.

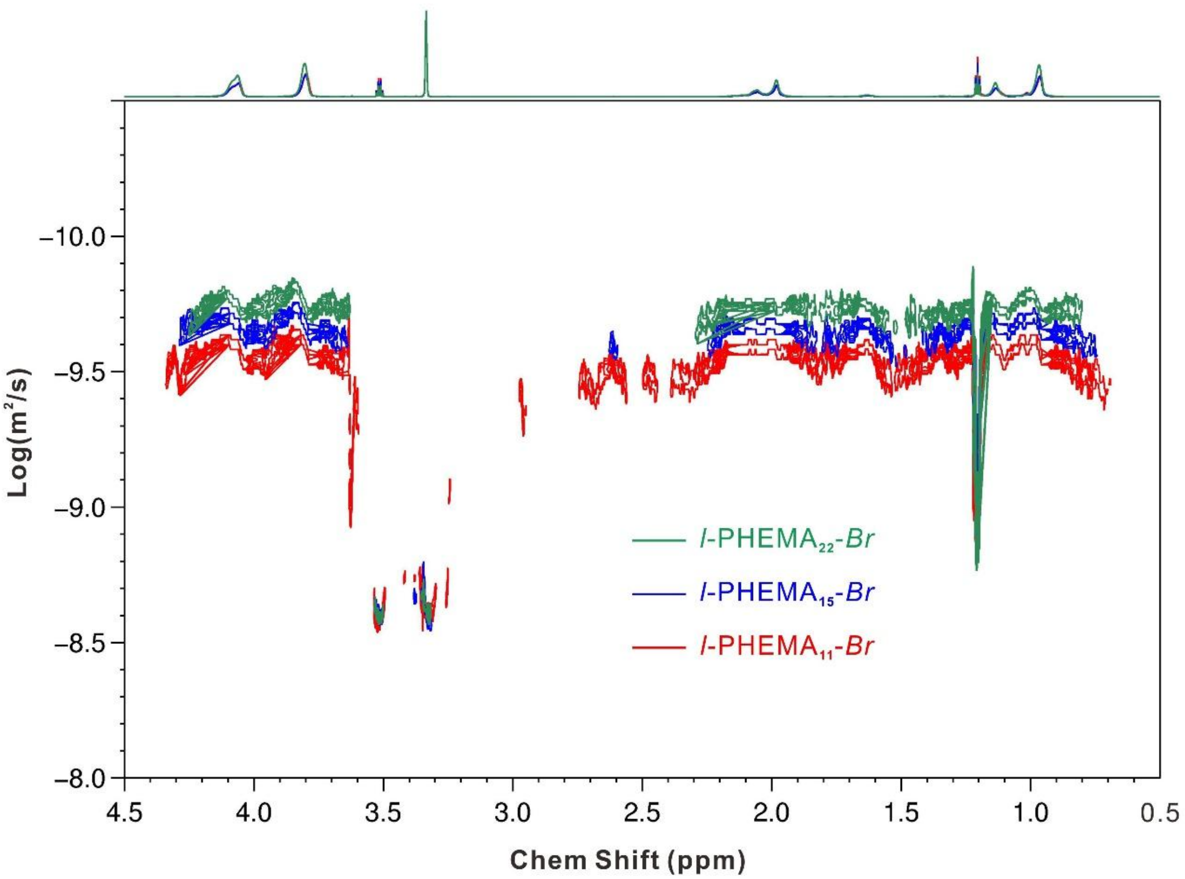

Supplementary Fig. 18. ^{1}H DOSY NMR spectra (850 MHz, MeOD, 298.3 K) of *l*-PHEMA$_{11}$-*Br*, *l*-PHEMA$_{15}$-*Br*, and *l*-PHEMA$_{22}$-*Br* showing their gradually increased hydrodynamic size.

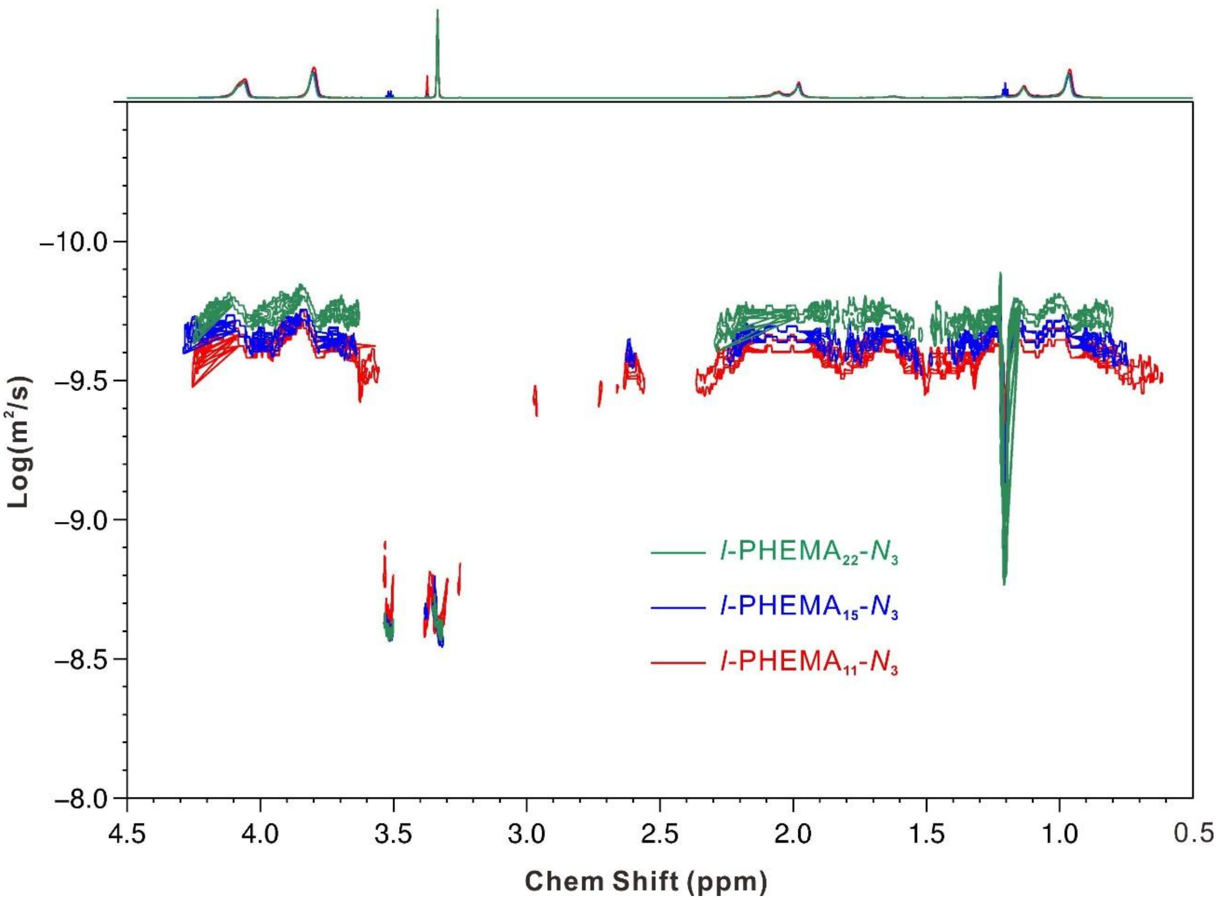

Supplementary Fig. 19. ^{1}H DOSY NMR spectra (850 MHz, MeOD, 298.3 K) of *l*-PHEMA$_{11}$-*N*$_3$, *l*-PHEMA$_{15}$-*N*$_3$, and *l*-PHEMA$_{22}$-*N*$_3$ showing their gradually increased hydrodynamic size.

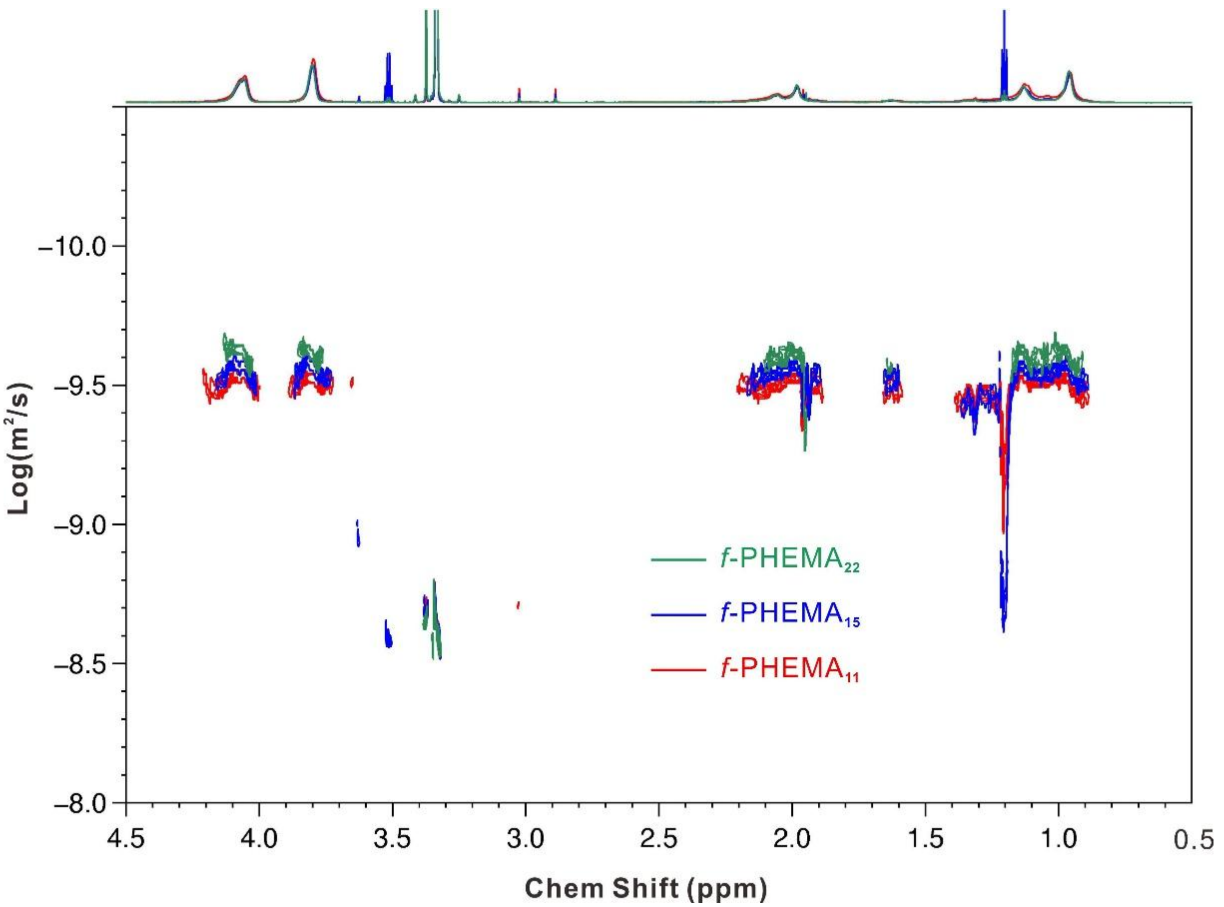

Supplementary Fig. 20. ^{1}H DOSY NMR spectra (850 MHz, MeOD, 298.3 K) of *f*-PHEMA11, *f*-PHEMA15, and *f*-PHEMA22 showing their gradually increased hydrodynamic size.

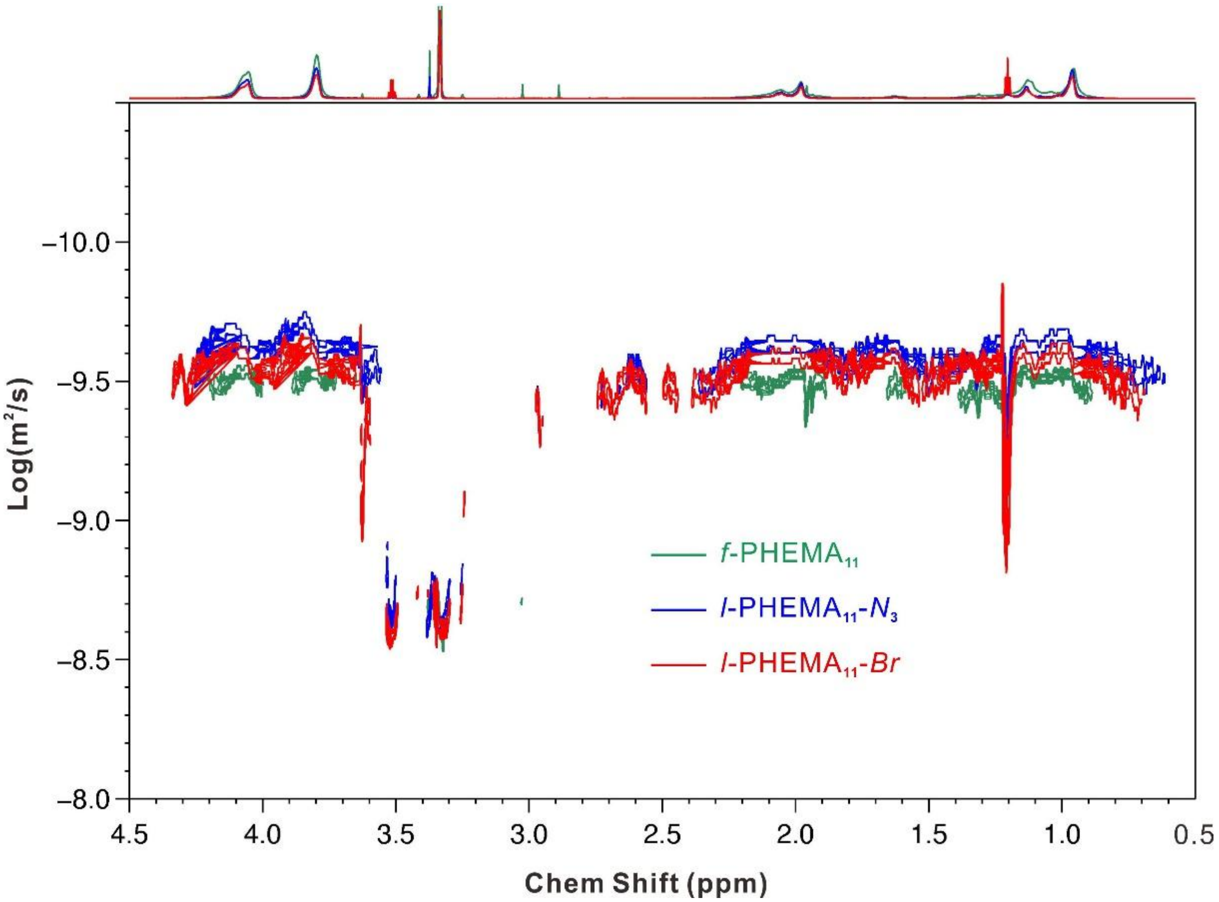

Supplementary Fig. 21. ^{1}H DOSY NMR spectra (850 MHz, MeOD, 298.3 K) of *l*-PHEMA$_{11}$-*Br*, *l*-PHEMA$_{11}$-*N*$_3$, and *f*-PHEMA$_{11}$. The folded polymer *f*-PHEMA$_{11}$ diffuses faster than its linear counterparts, indicating a decreased hydrodynamic size after molecular folding.

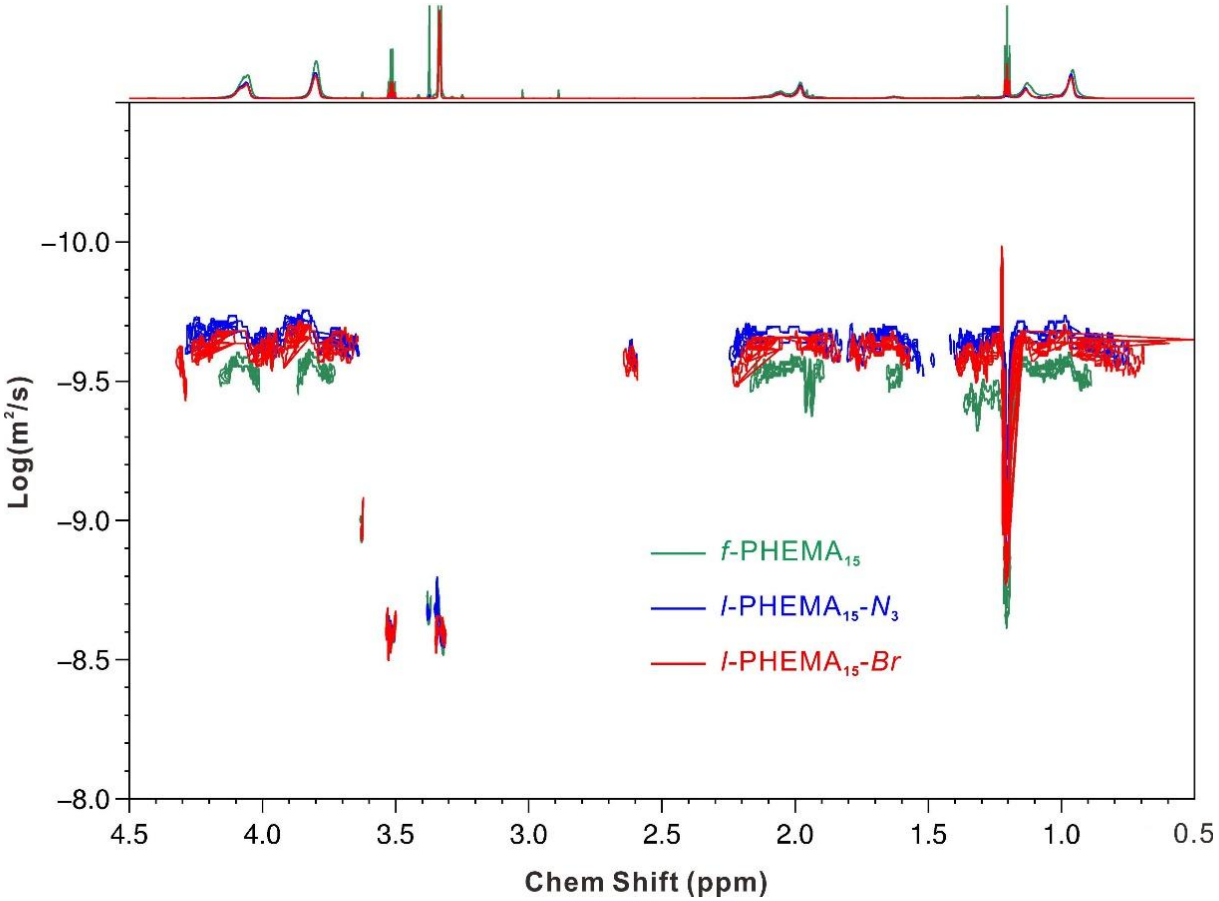

Supplementary Fig. 22. ^{1}H DOSY NMR spectra (850 MHz, MeOD, 298.3 K) of *l*-PHEMA$_{15}$-*Br*, *l*-PHEMA$_{15}$-*N*$_3$, and *f*-PHEMA$_{15}$. The folded polymer *f*-PHEMA$_{15}$ diffuses faster than its linear counterparts, indicating a decreased hydrodynamic size after molecular folding.

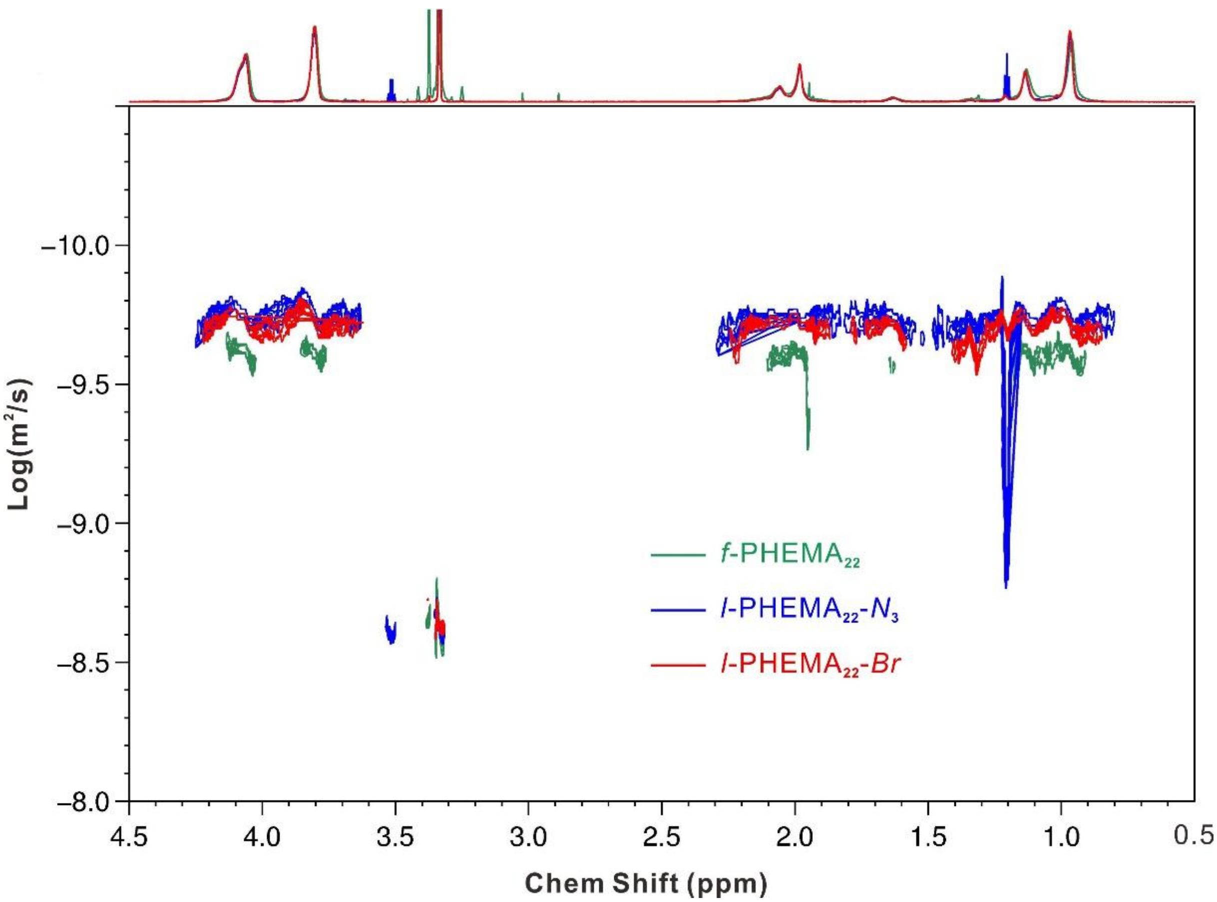

Supplementary Fig. 23. ^{1}H DOSY NMR spectra (850 MHz, MeOD, 298.3 K) of *l*-PHEMA$_{22}$-*Br*, *l*-PHEMA$_{22}$-*N*$_3$, and *f*-PHEMA$_{22}$. The folded polymer *f*-PHEMA$_{22}$ diffuses faster than its linear counterparts, indicating a decreased hydrodynamic size after molecular folding.

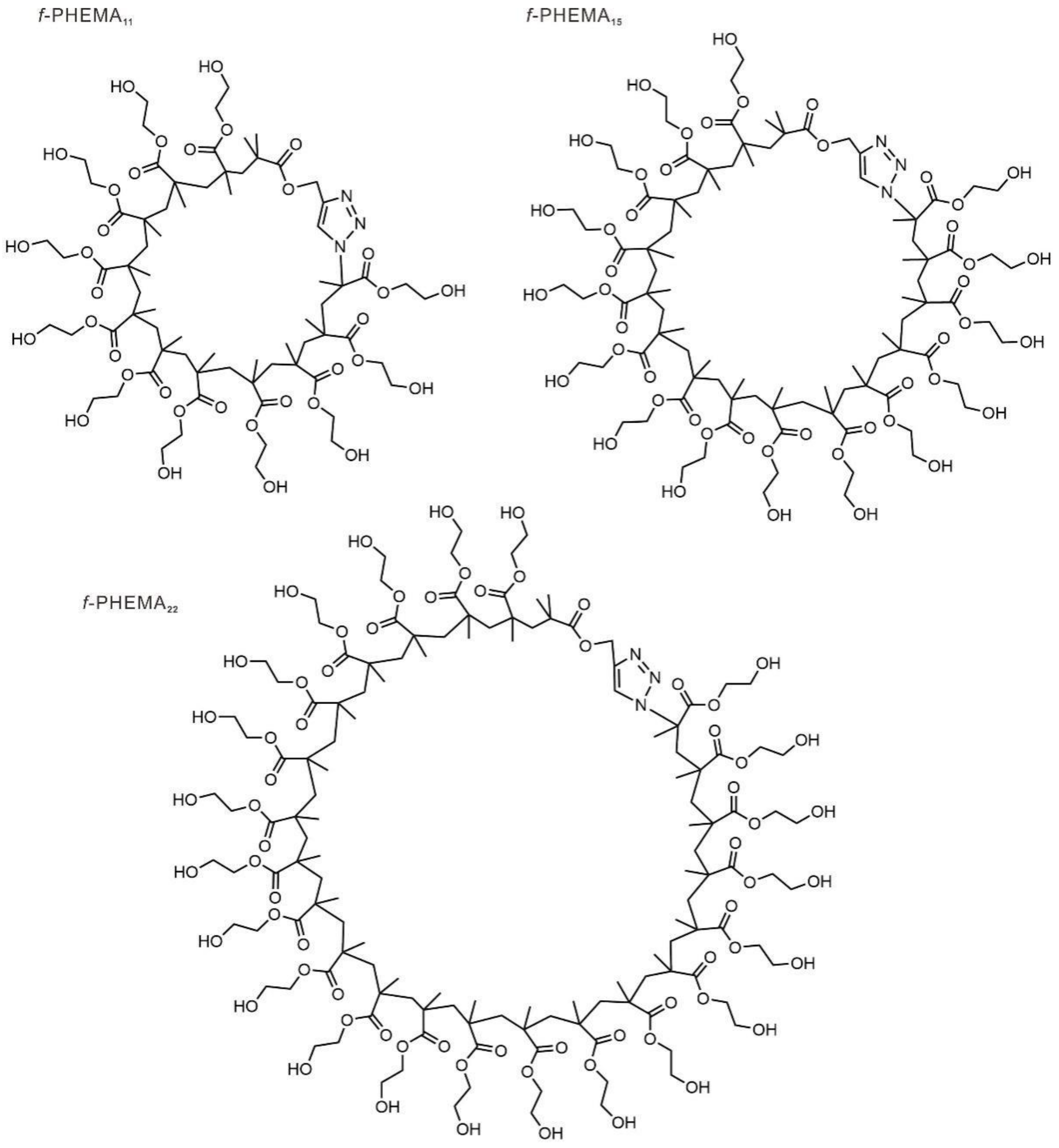

Supplementary Fig. 24. Molecular structures of *f*-PHEMA$_{11}$, *f*-PHEMA$_{15}$, and *f*-PHEMA$_{22}$.

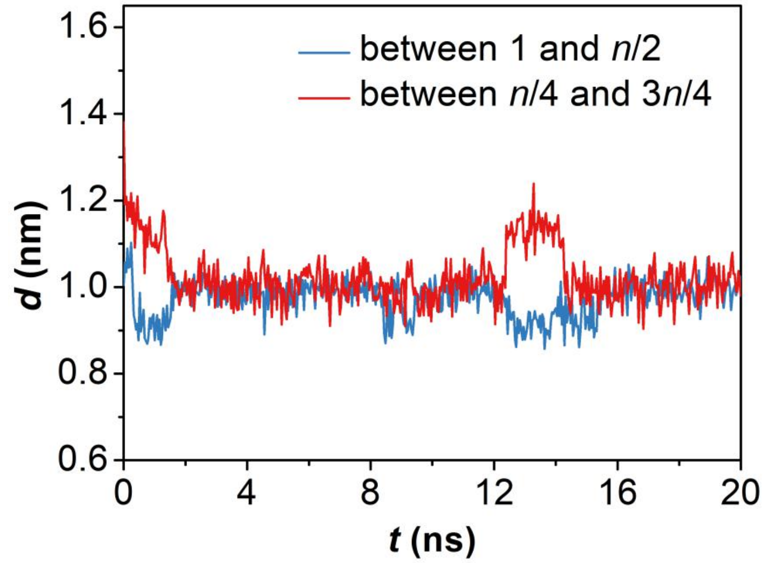

Supplementary Fig. 25. Calculation of the lengths for the two major axes of a single *f*-PHEMA$_{15}$ structure in Fig. 1e of the main text. The distances are calculated between 1 and n/2 monomers for one axis and between n/4 and 3n/4 monomers for the second axis. These numbers are very similar over 20 ns, except for some fluctuations within the time window of 12 ~14 ns. It is another evidence that the structure indeed remains rather cyclic.

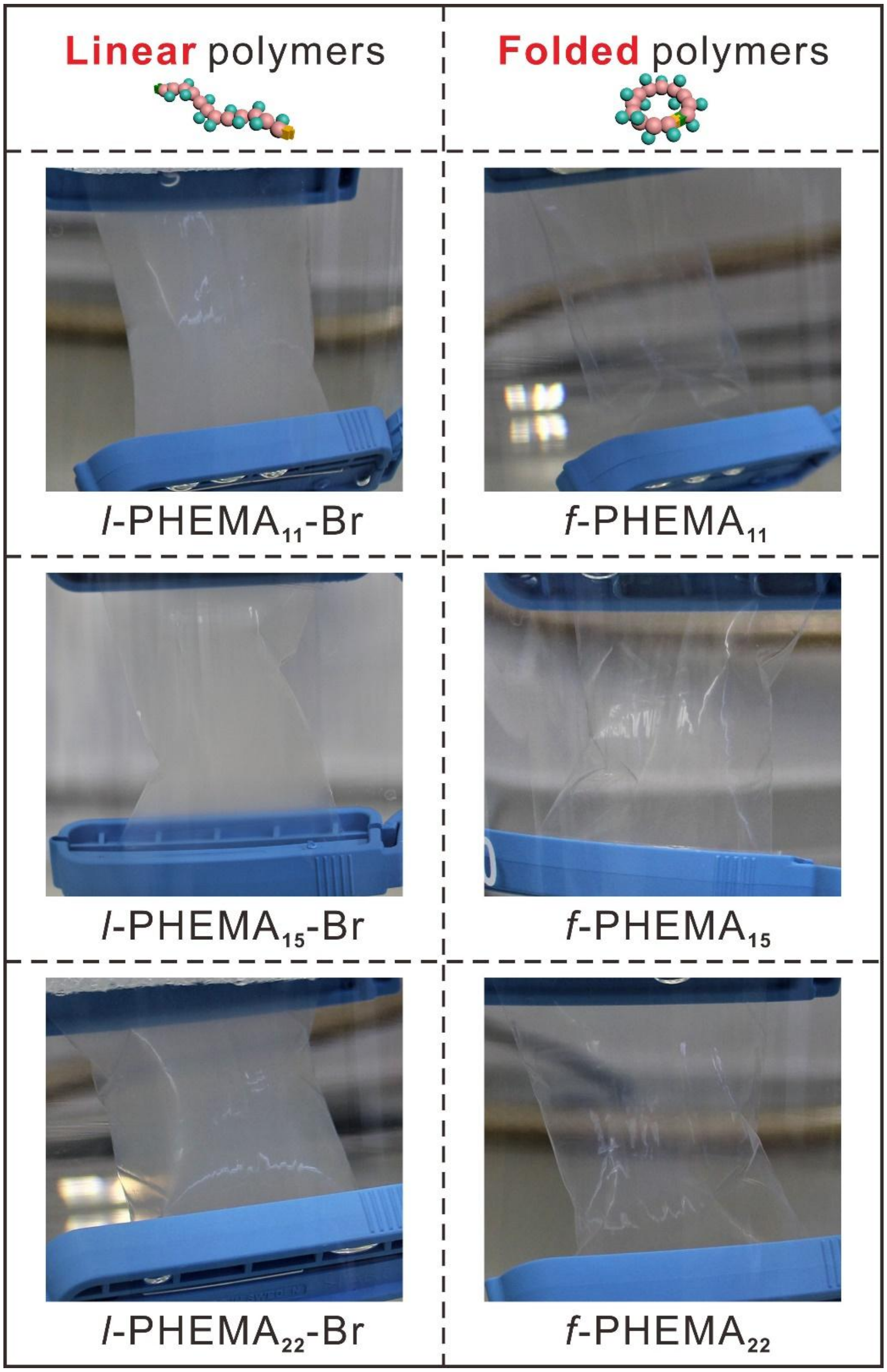

Supplementary Fig. 26. Self-assembly solutions of linear polymers l-PHEMA$_n$-*Br* and folded polymers f-PHEMA$_n$ after dialysis against deionized water for 1 h.

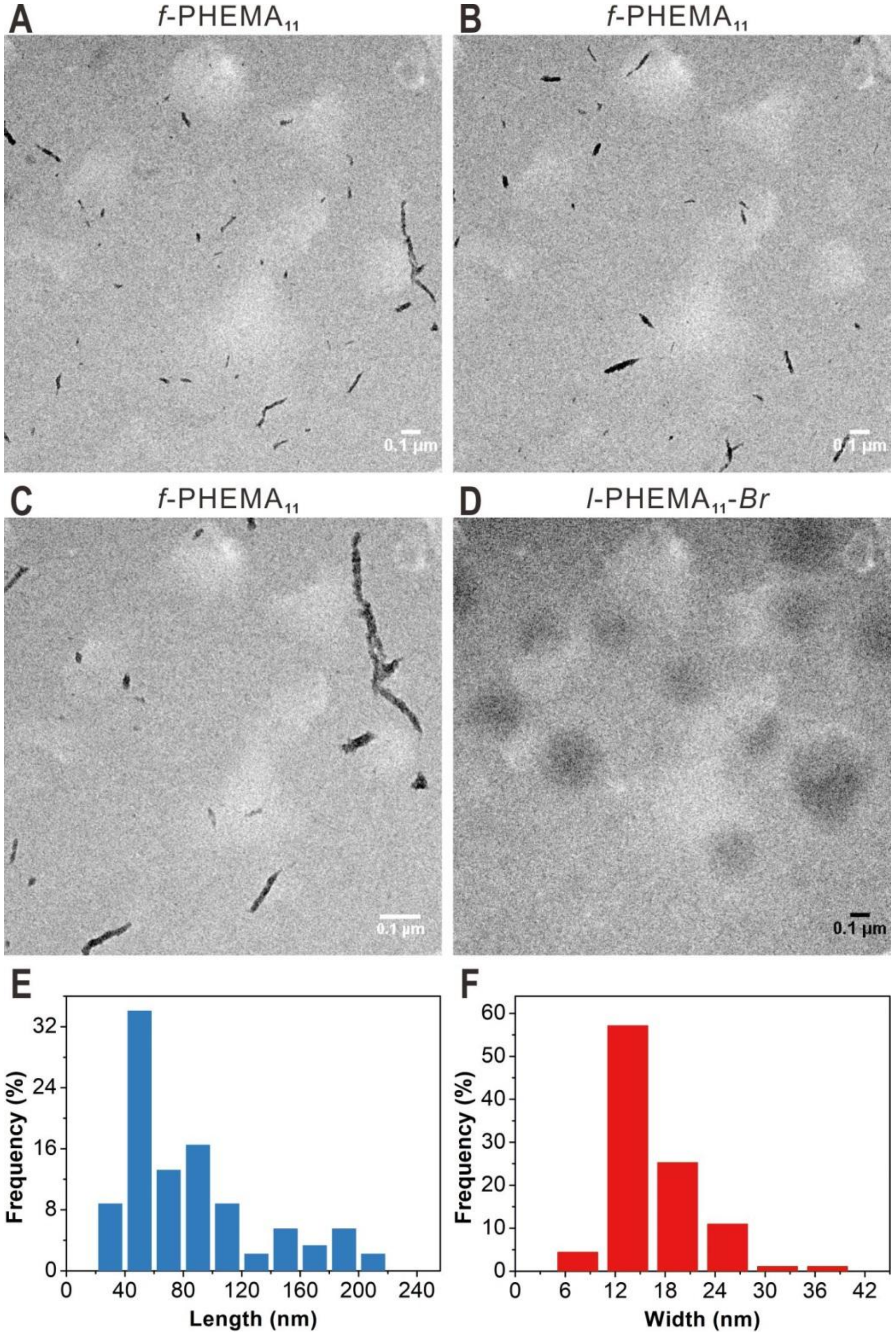

Supplementary Fig. 27. TEM images for comparing the self-assembly morphologies of (A-C) folded polymer *f*-PHEMA$_{11}$ and (D) linear polymer *l*-PHEMA$_{11}$-*Br*. Note that the solution of *l*-PHEMA$_{11}$-*Br* turned turbid after dialysis for one hour (Supplementary Fig. 26) and some gel-like precipitation was observed three days later. Therefore, the TEM sample of *l*-PHEMA$_{11}$-*Br* was prepared by dropping the upper solution on TEM grids. (E and F) Statistical analysis for the length (E) and width (F) distributions of the self-assembled nanostructures from *f*-PHEMA$_{11}$ (more than 50 nanoobjects in TEM images were measured and analyzed).

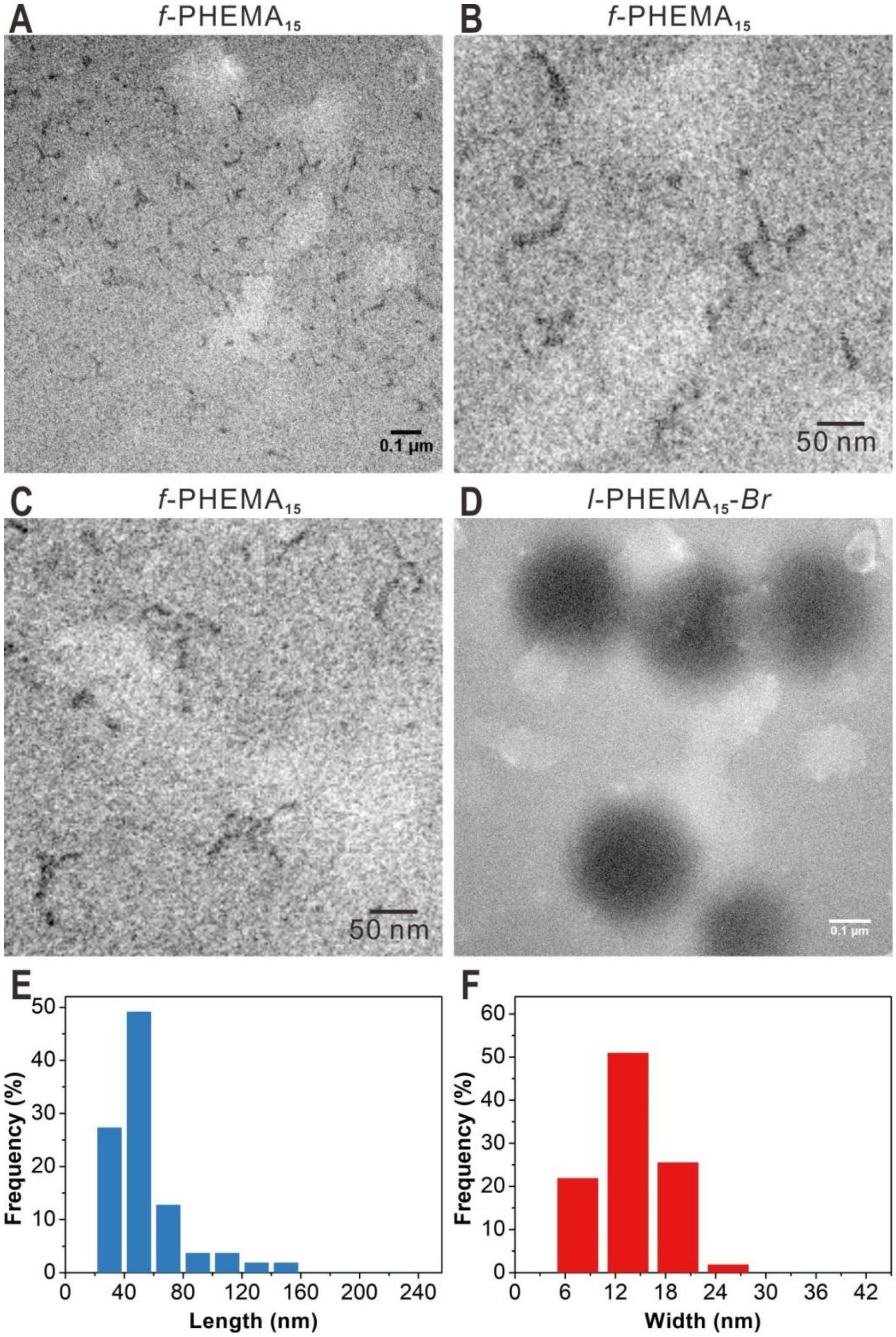

Supplementary Fig. 28. TEM images for comparing the self-assembly morphologies of (A-C) folded polymer *f*-PHEMA$_{15}$ and (D) linear polymer *l*-PHEMA$_{15}$-*Br*. Note that the solution of *l*-PHEMA$_{15}$-*Br* turned turbid after dialysis for one hour (Supplementary Fig. 26) and some gel-like precipitation was observed three days later. Therefore, the TEM sample of *l*-PHEMA$_{15}$-*Br* was prepared by dropping the upper solution on TEM grids. (E and F) Statistical analysis for the length (E) and width (F) distributions of the self-assembled nanostructures from *f*-PHEMA$_{15}$ (more than 50 nanoobjects in TEM images were measured and analyzed).

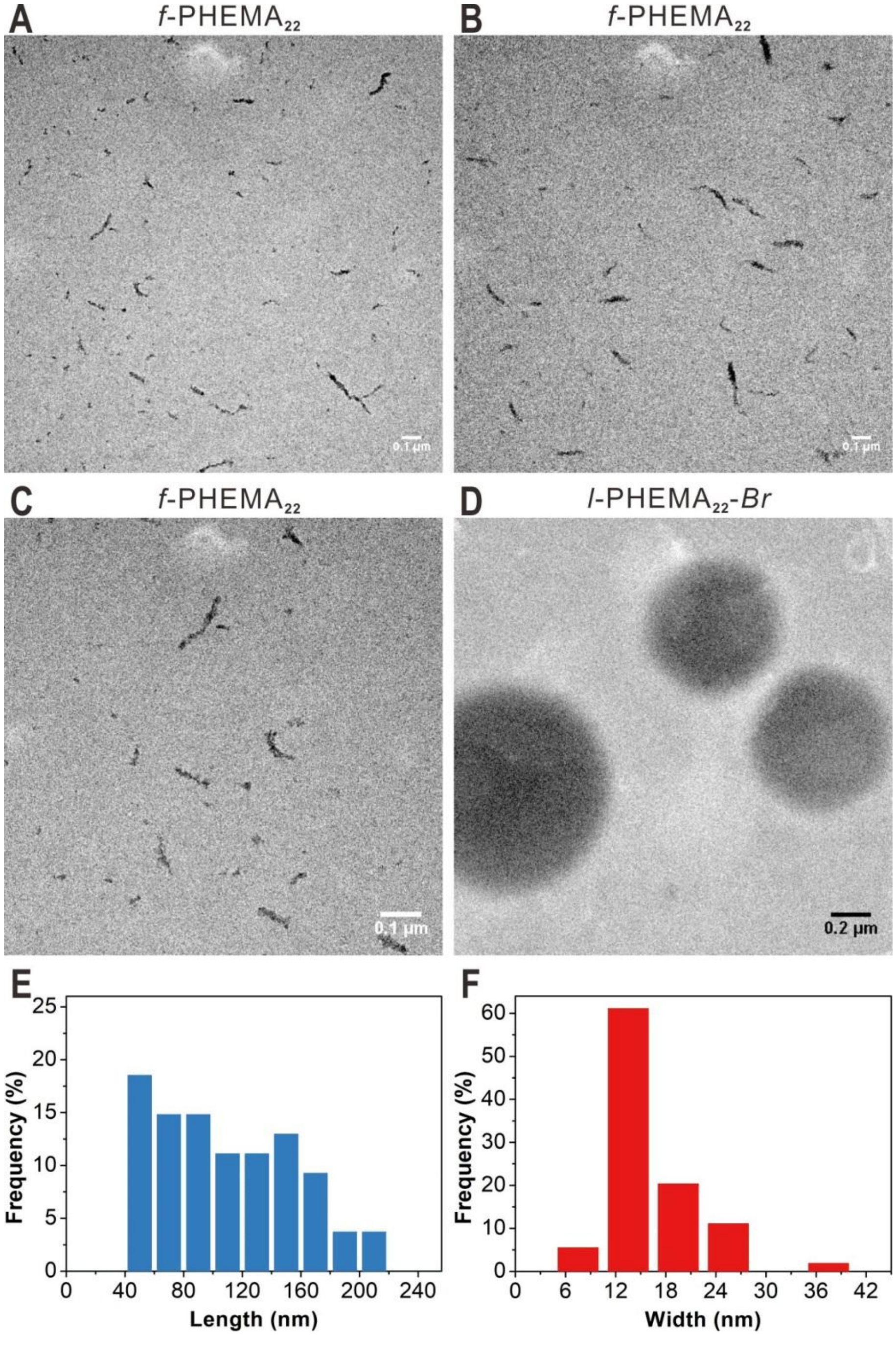

Supplementary Fig. 29. TEM images for comparing the self-assembly morphologies of (A-C) folded polymer *f*-PHEMA$_{22}$ and (D) linear polymer *l*-PHEMA$_{22}$-*Br*. Note that the solution of *l*-PHEMA$_{22}$-*Br* turned turbid after dialysis for one hour (Supplementary Fig. 26) and some gel-like precipitation was observed three days later. Therefore, the TEM sample of *l*-PHEMA$_{22}$-*Br* was prepared by dropping the upper solution on TEM grids. (E and F) Statistical analysis for the length (E) and width (F) distributions of the self-assembled nanostructures from *f*-PHEMA$_{22}$ (more than 50 nanoobjects in TEM images were measured and analyzed).

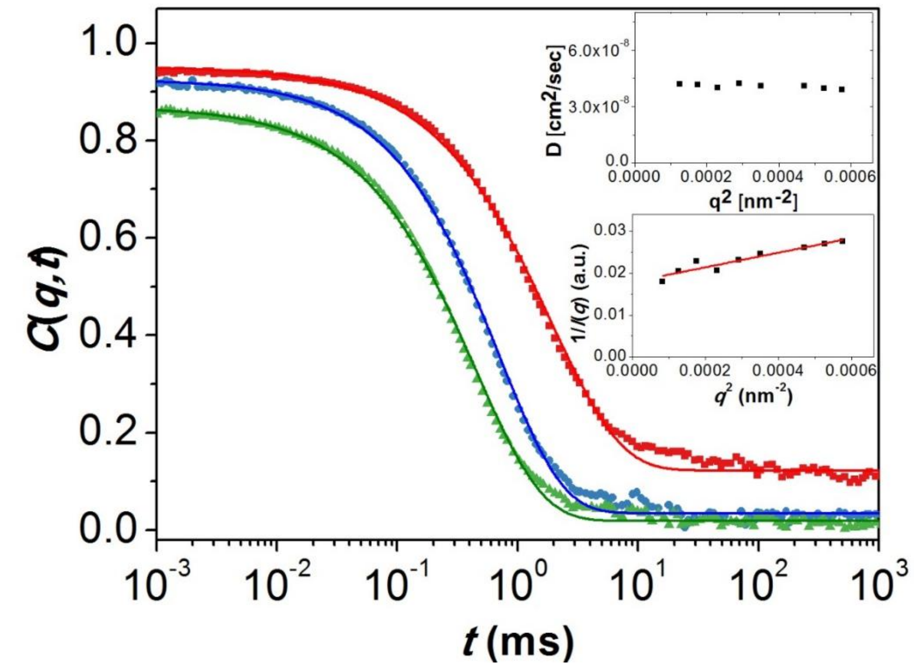

Supplementary Fig. 30. Relaxation functions $C(q,t)$ for the translation motion of *f*-PHEMA$_{11}$ in water at 1 mg/mL and 293 K at different scattering angles [50° (red squares), 90° (blue circles), and 130° (green triangles)] corresponding to scattering wave vector q = 0.011 nm^{-1}, q = 0.0187 nm^{-1}, q = 0.024 nm^{-1}, respectively. Inset: $D(q = 0) = 4.34 \times 10^{-8}$ cm^2/sec; $R_h = 54 \pm 2$ nm.

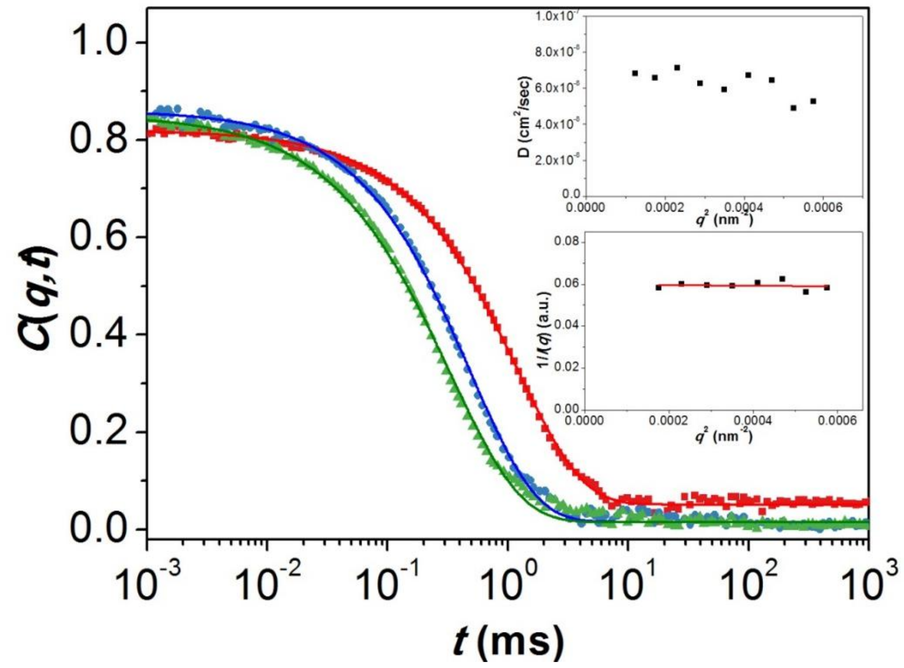

Supplementary Fig. 31. Relaxation functions $C(q,t)$ for the translation motion of *f*-PHEMA$_{15}$ in water at 1 mg/mL and 293 K at different scattering angles [50° (red squares), 90° (blue circles), and 130° (green triangles)] corresponding to scattering wave vector q = 0.011 nm^{-1}, q = 0.0187 nm^{-1}, q = 0.024 nm^{-1}, respectively. Inset: $D(q = 0) = 6.75\times10^{-8}$ cm^2/sec; $R_h = 36 \pm 2$ nm.

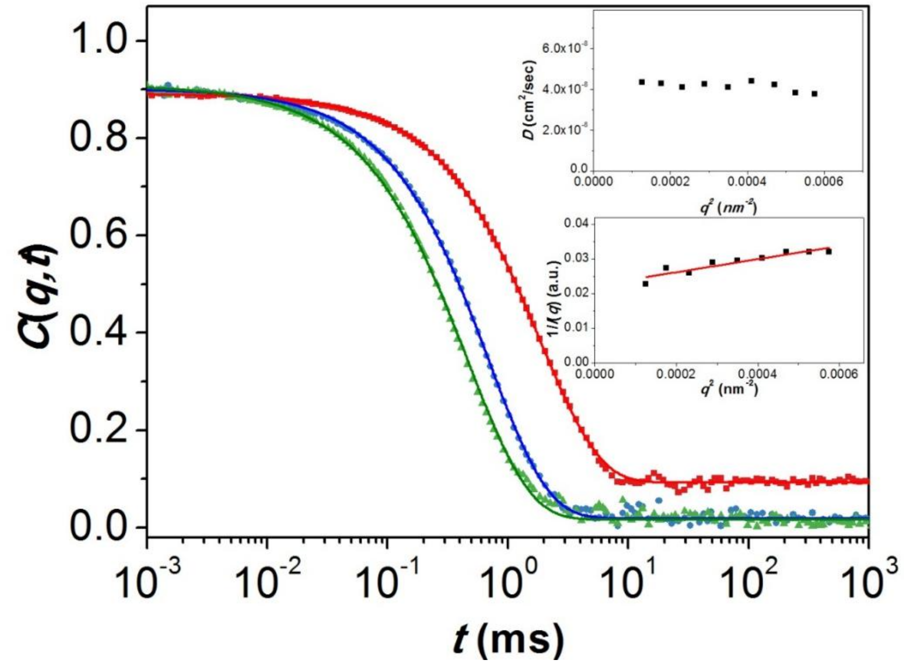

Supplementary Fig. 32. Relaxation functions $C(q,t)$ for the translation motion of *f*-PHEMA$_{22}$ in water at 1 mg/mL and 293 K at different scattering angles [50° (red squares), 90° (blue circles), and 130° (green triangles)] corresponding to scattering wave vector q = 0.011 nm^{-1}, q = 0.0187 nm^{-1}, q = 0.024 nm^{-1}, respectively. Inset: $D(q = 0) = 4.21\times10^{-8}$ cm^2/sec; R_h = 58 ± 3 nm.

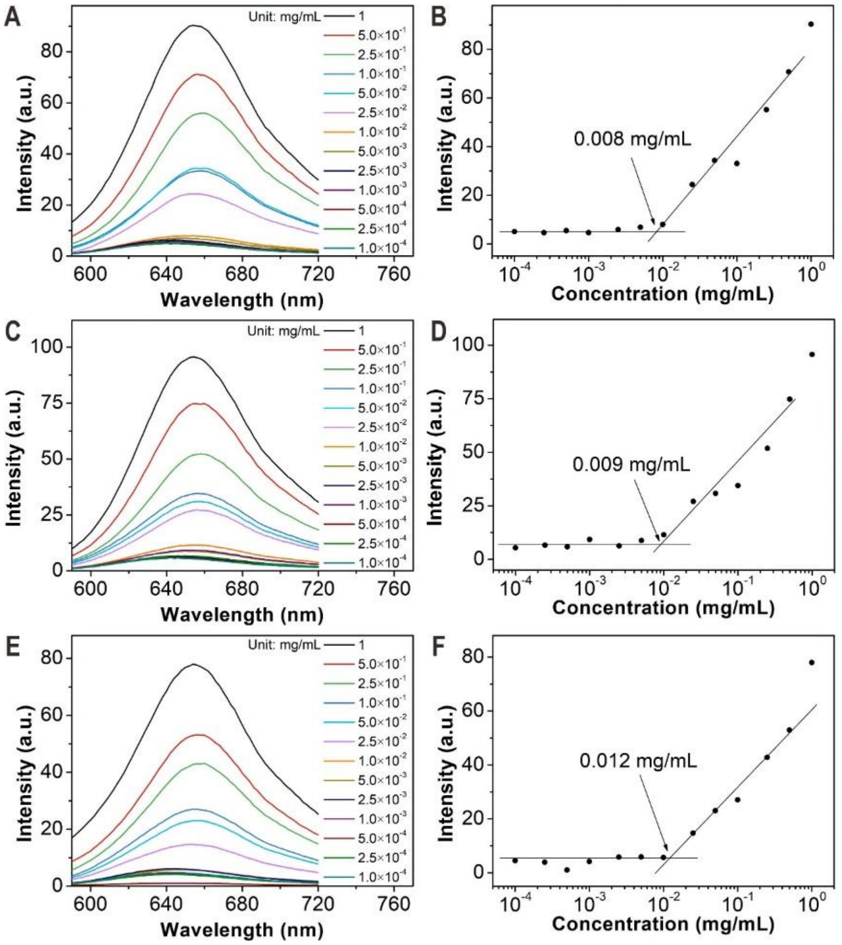

Supplementary Fig. 33. (A) Fluorescence emission spectra of *f*-PHEMA$_{11}$ assemblies of varying concentrations after loading with Nile Red. Nile Red in aqueous solution shows low fluorescence emission intensity after excitation with 550 nm light. However, the intensity increases significantly if the probe is encapsulated into a hydrophobic environment. As shown in this figure, the fluorescence emission intensity is very weak when the assembly concentration is low such as 0.001 mg mL^{-1}, indicating that Nile Red molecules were dispersed in an aqueous environment. If the concentration is high, for example 0.5 mg mL^{-1}, the characteristic emission peak of Nile Red can be clearly observed, showing the encapsulation of the fluorescent probe in a hydrophobic environment. (B) Determination of the CAC of *f*-PHEMA$_{11}$ by plotting the emission intensity at 654 nm over the concentration of the assemblies. The dots can be divided into two groups connected by two straight lines based on the slope. The CAC was therefore determined from the intersection of the two lines as 0.008 mg mL^{-1}, indicating that *f*-PHEMA$_{11}$ starts to assemble and form some hydrophobic internal microenvironments at this concentration. (C) Fluorescence emission spectra of *f*-PHEMA$_{15}$ assemblies of varying concentrations after loading with Nile Red. (D) Determination of the CAC of *f*-PHEMA$_{15}$. (E) Fluorescence emission spectra of *f*-PHEMA$_{22}$ assemblies of varying concentrations after loading with Nile Red. (F) Determination of the CAC of *f*-PHEMA$_{22}$.

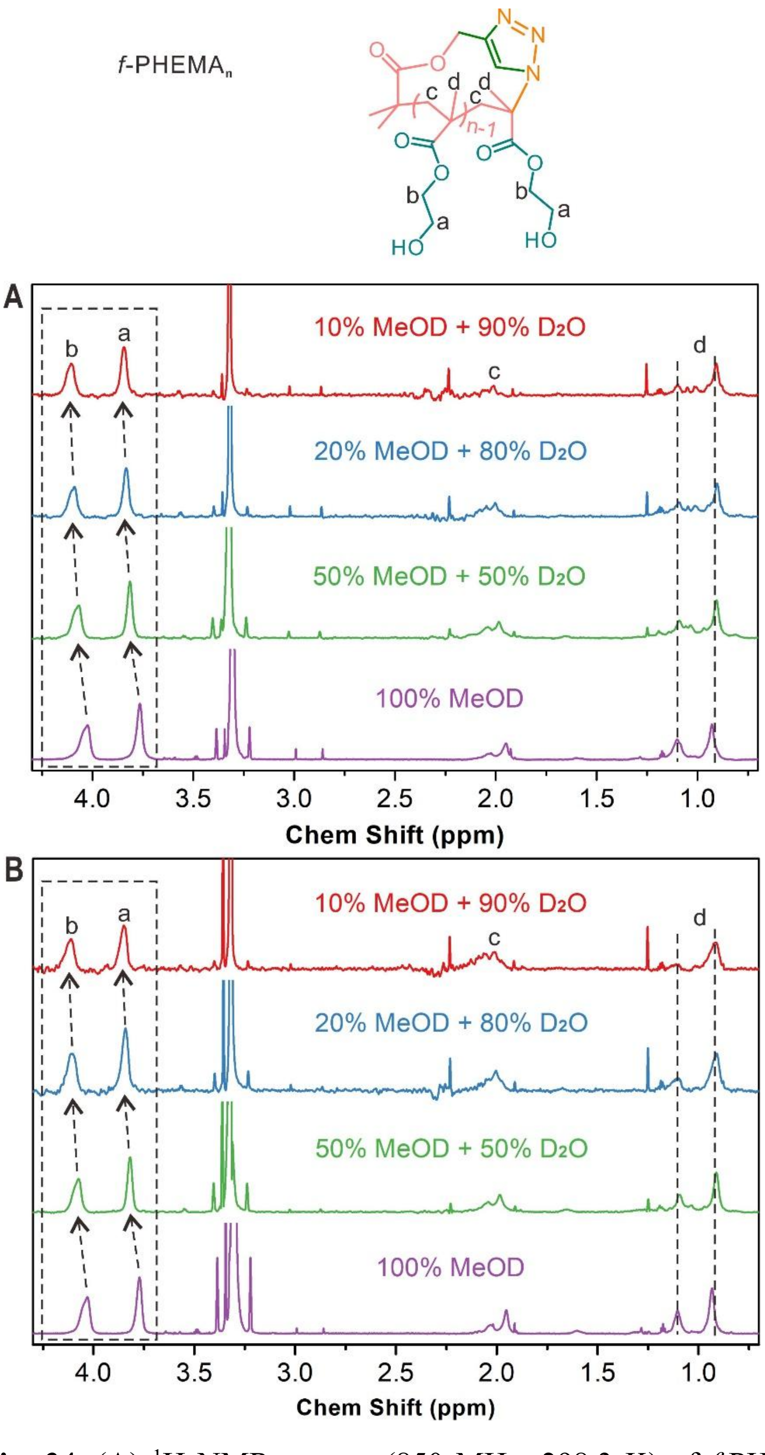

Supplementary Fig. 34. (A) ^{1}H NMR spectra (850 MHz, 298.3 K) of *f*-$PHEMA_{11}$ in mixture solvents of MeOD and D_2O with gradually tuned volume ratios. (B) ^{1}H NMR spectra (850 MHz, 298.3 K) of *f*-$PHEMA_{22}$ in mixture solvents of MeOD and D_2O with gradually tuned volume ratios. Tetramethylsilane was used as an internal standard for calibrating the chemical shifts.

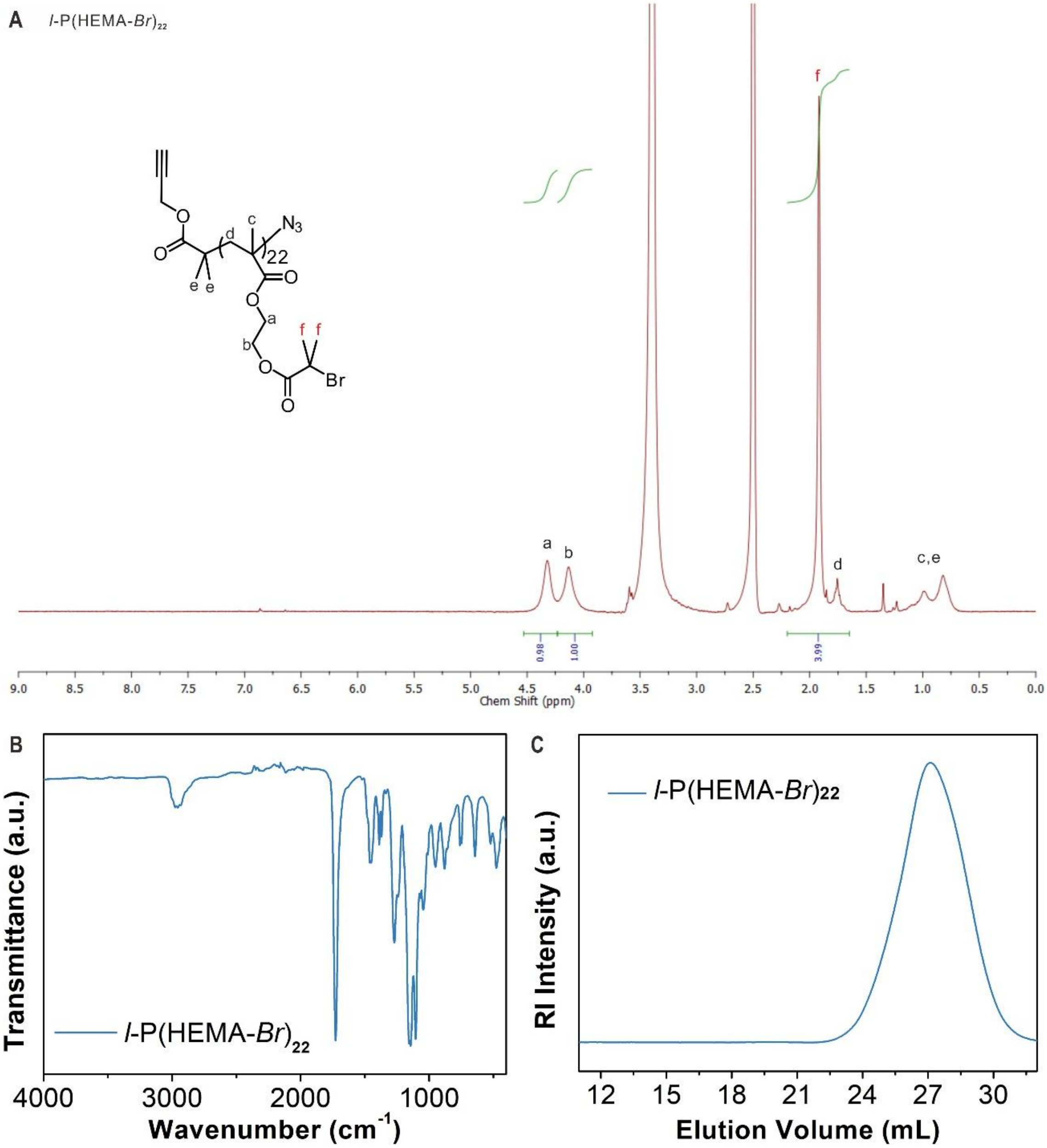

Supplementary Fig. 35. Characterizations of linear macroinitiator *l*-P(HEMA-Br)$_{22}$. (A) ^{1}H NMR spectrum (300 MHz, 298.3 K) in DMSO-d$_6$. The integral ratio of signals **a** plus **b** and signals **f** plus **d** is approximately 2:4, which confirms the full conversion of hydroxyls to initiation groups. (B) FTIR spectrum. (C) GPC curve measured using DMF as the eluent with PMMA standards for calibration.

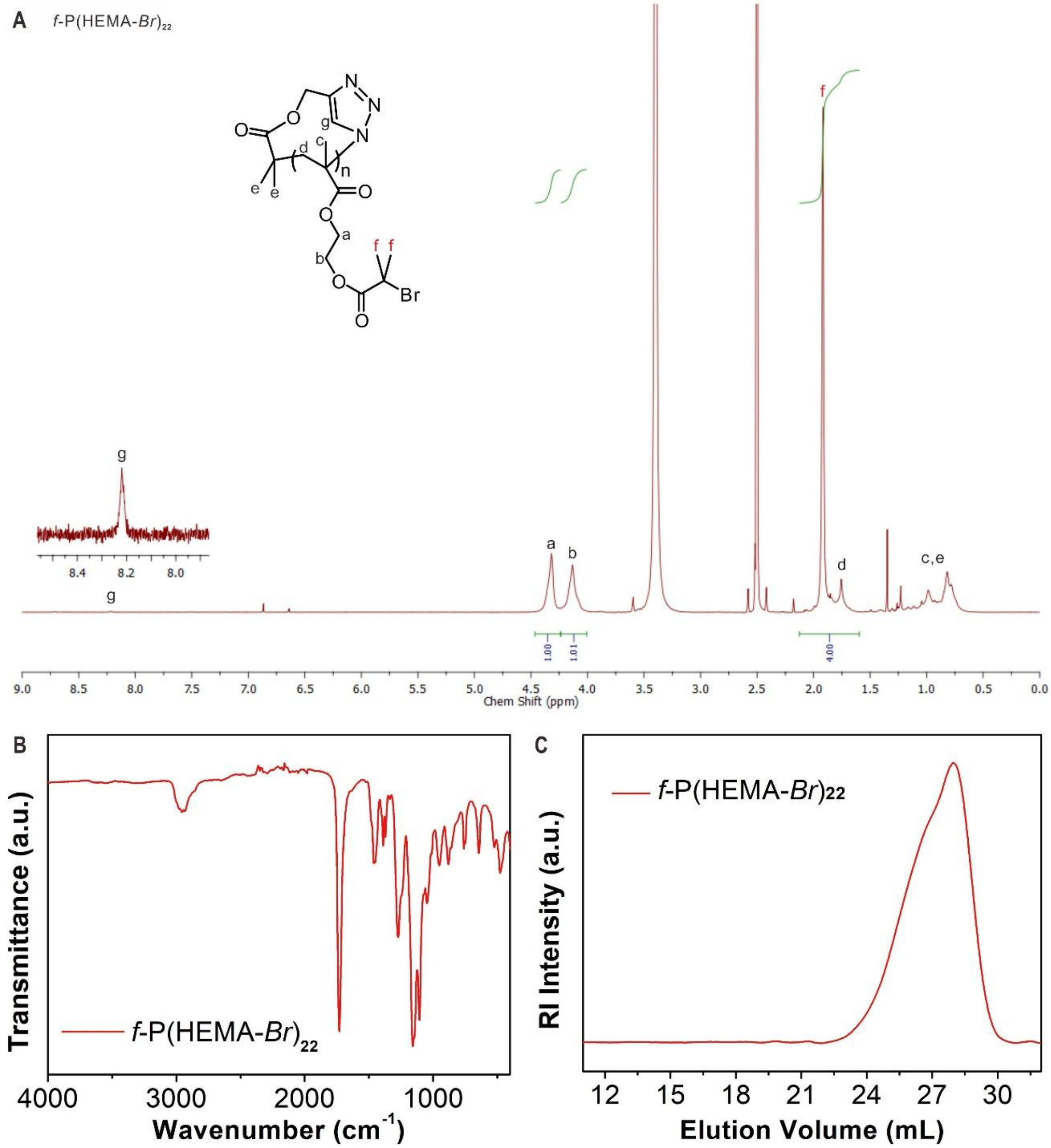

Supplementary Fig. 36. Characterizations of cyclic macroinitiator *f*-P(HEMA-Br)$_{22}$. (A) ^{1}H NMR spectrum (300 MHz, 298.3 K) in DMSO-d$_6$. The integral ratio of signals **a** plus **b** and signals **f** plus **d** is approximately 2:4, which confirms the full conversion of hydroxyls to initiation groups. (B) FTIR spectrum. (C) GPC curve measured using DMF as the eluent with PMMA standards for calibration.

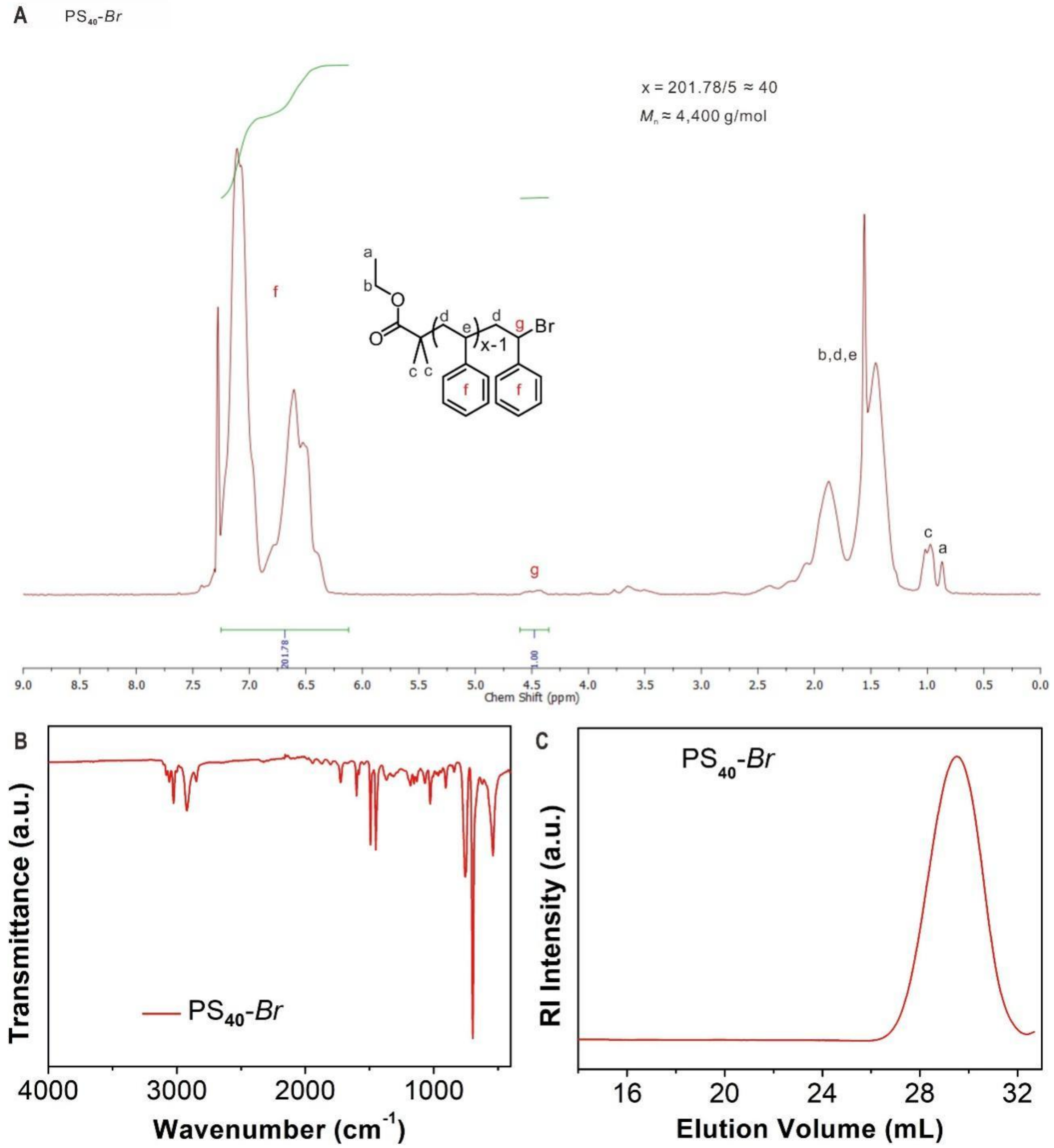

Supplementary Fig. 37. Characterizations of PS_{40}-*Br*. (A) ^{1}H NMR spectrum (300 MHz, 298.3 K) in $CDCl_3$-d. The integral ratio of signal **g** and signal **f** was used to calculated the repeating number (x) and the molecular weight ($M_{n,NMR}$). (B) FTIR spectrum. (C) GPC curve measured using DMF as the eluent with PS standards for calibration.

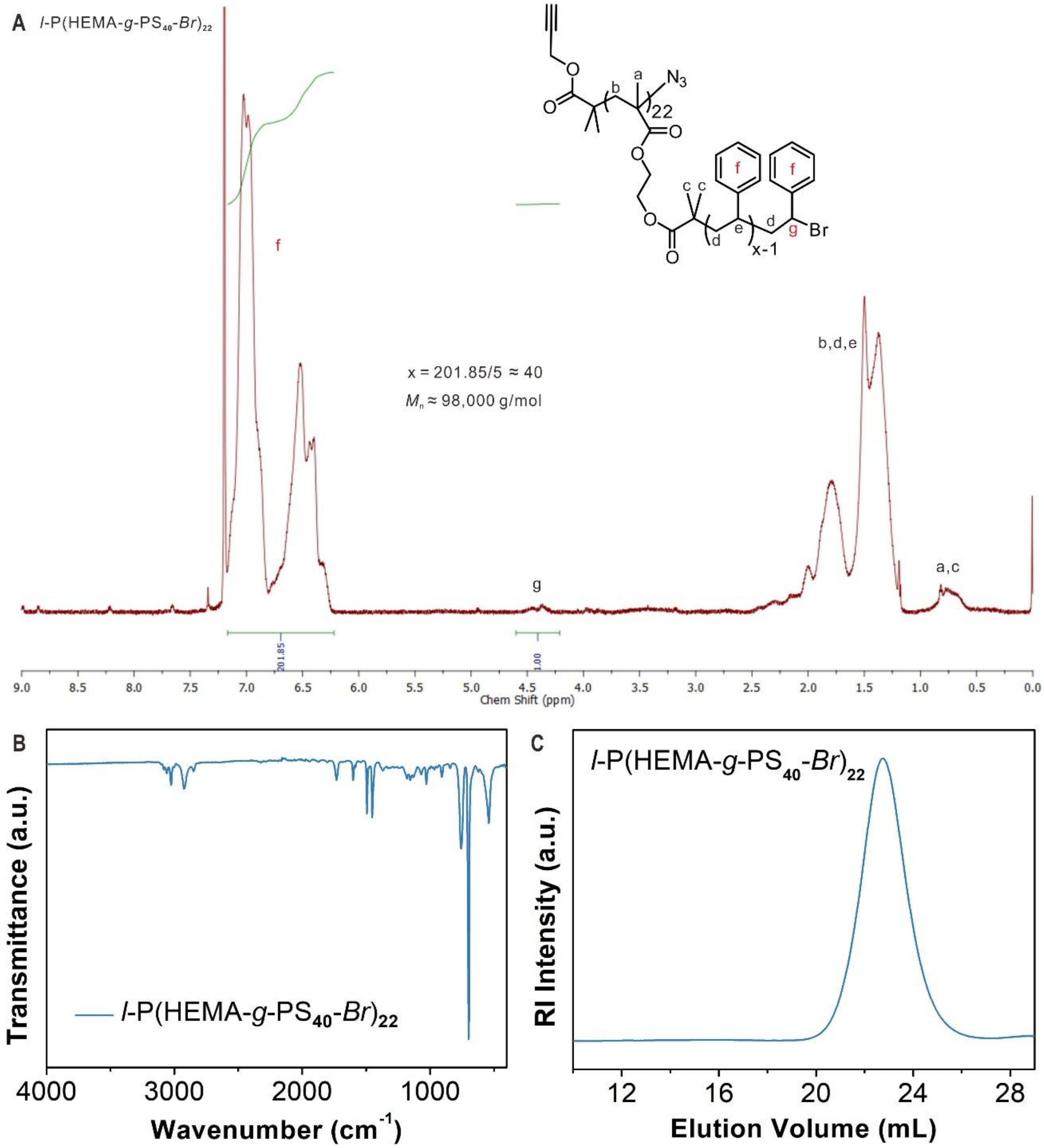

Supplementary Fig. 38. Characterizations of *l*-P(HEMA-*g*-PS_{40}-*Br*)$_{22}$. (A) ^{1}H NMR spectrum (700 MHz, 298.3 K) in $CDCl_3$-d. The integral ratio of signal **g** and signal **f** was used to calculated the repeating number (x) and the molecular weight ($M_{n,NMR}$). (B) FTIR spectrum. (C) GPC curve measured using DMF as the eluent with PS standards for calibration.

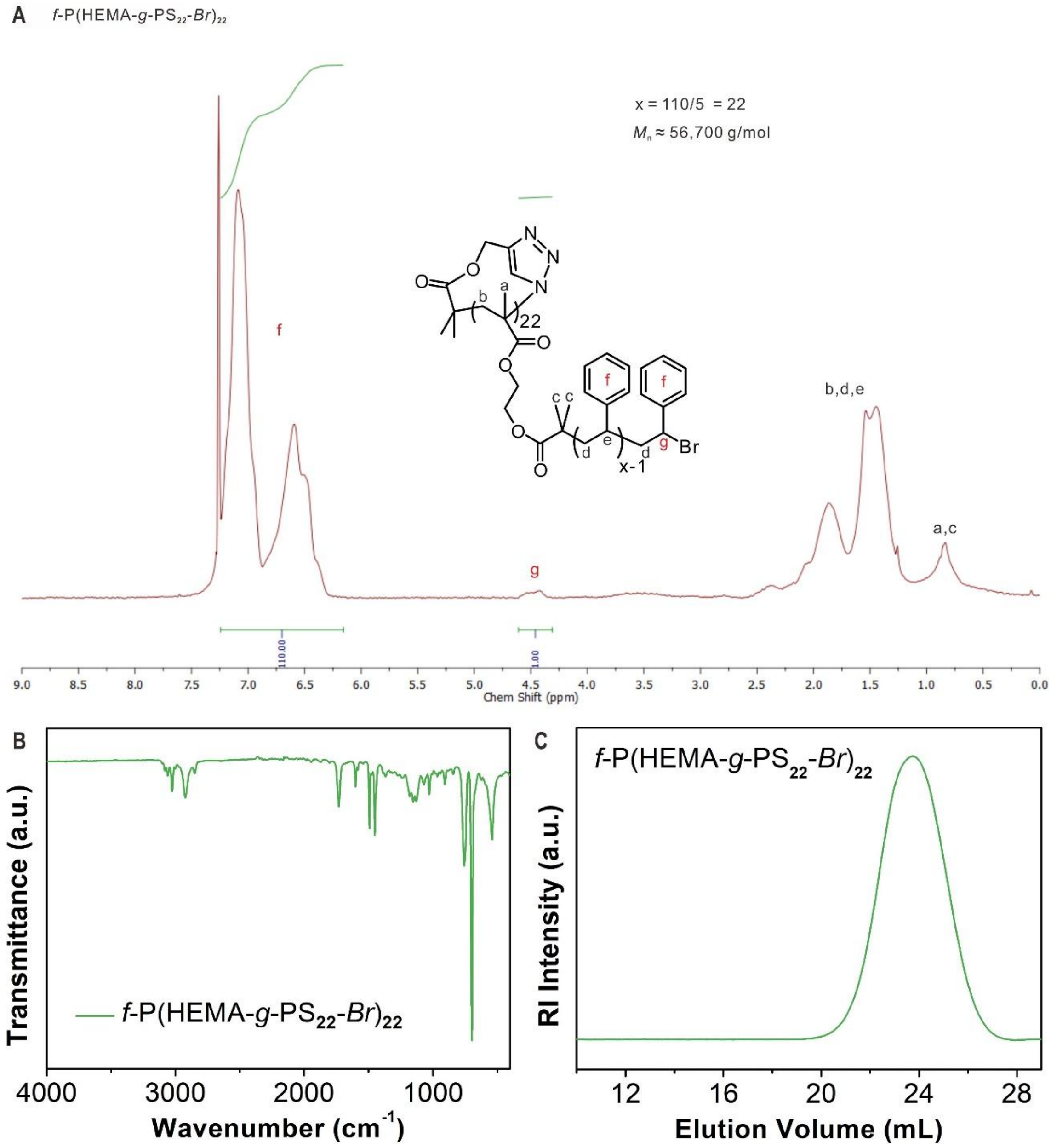

Supplementary Fig. 39. Characterizations of *f*-P(HEMA-*g*-PS22-*Br*)22. (A) ^{1}H NMR spectrum (300 MHz, 298.3 K) in $CDCl_3$-d. The integral ratio of signal **g** and signal **f** was used to calculated the repeating number (x) and the molecular weight ($M_{n,NMR}$). (B) FTIR spectrum. (C) GPC curve measured using DMF as the eluent with PS standards for calibration.

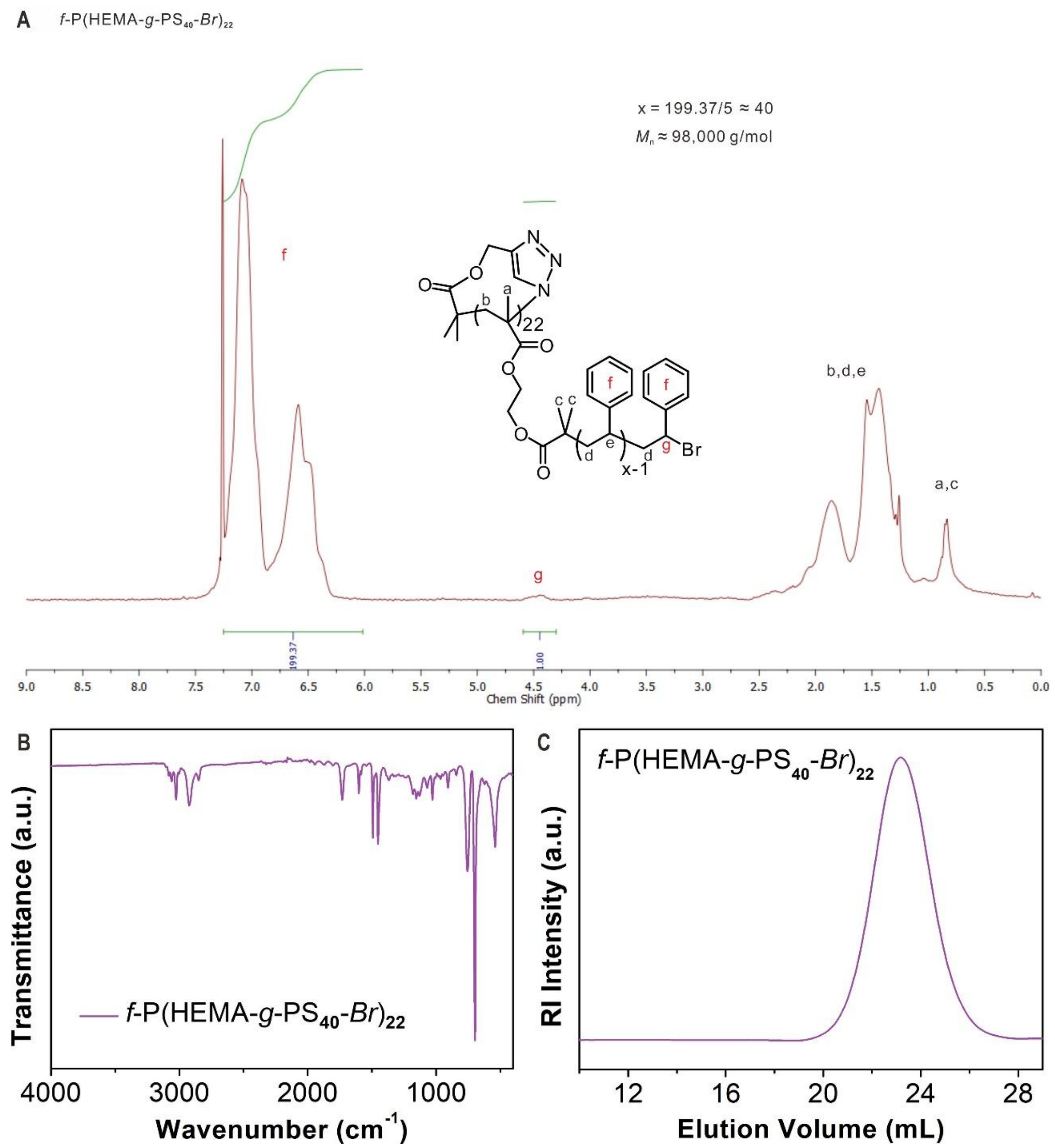

Supplementary Fig. 40. Characterizations of *f*-P(HEMA-*g*-PS_{40}-*Br*)$_{22}$. (A) ^{1}H NMR spectrum (300 MHz, 298.3 K) in $CDCl_3$-d. The integral ratio of signal **g** and signal **f** was used to calculated the repeating number (x) and the molecular weight ($M_{n,NMR}$). (B) FTIR spectrum. (C) GPC curve measured using DMF as the eluent with PS standards for calibration.

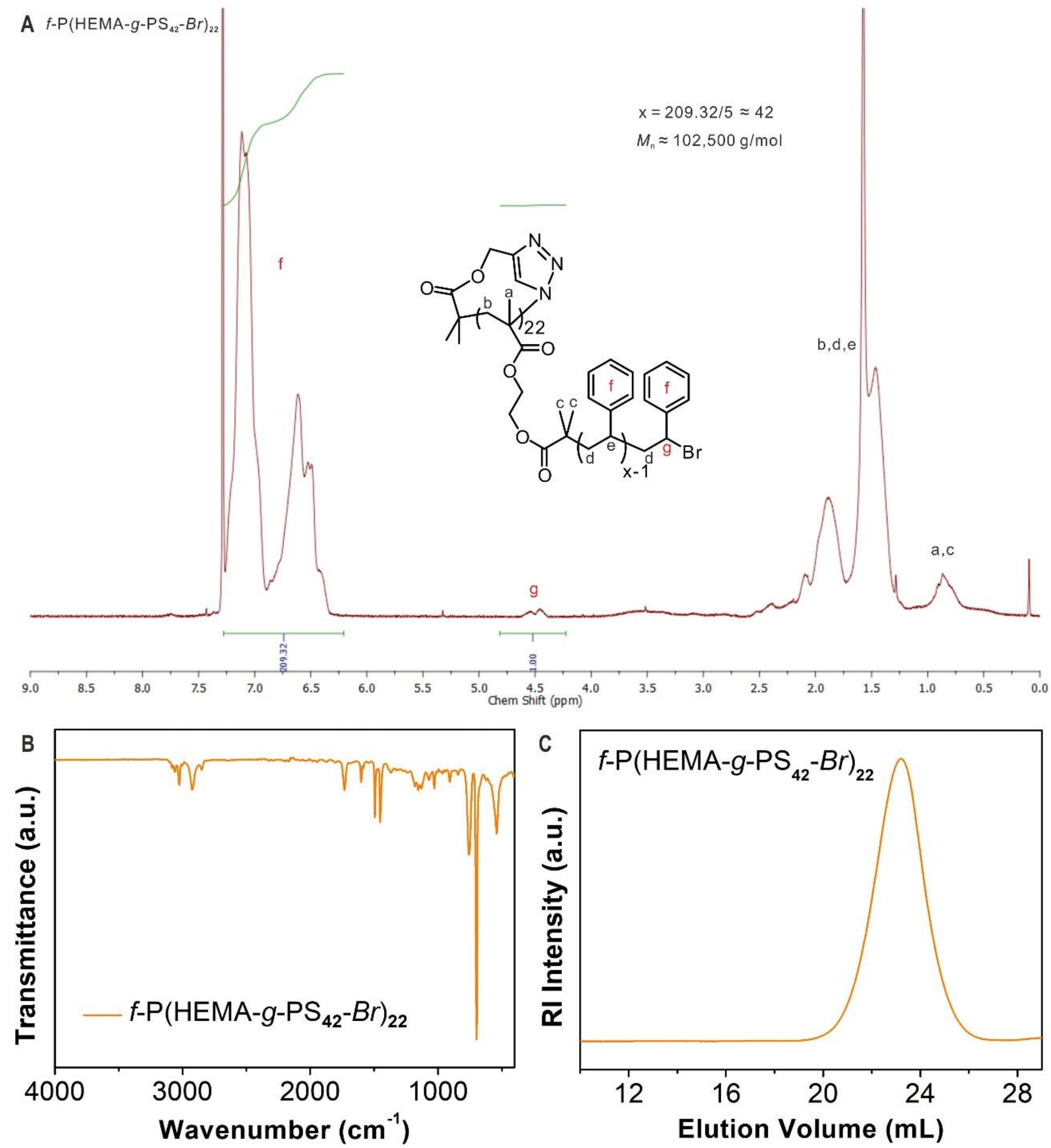

Supplementary Fig. 41. Characterizations of *f*-P(HEMA-*g*-PS$_{42}$-*Br*)$_{22}$. (A) ^{1}H NMR spectrum (700 MHz, 298.3 K) in CDCl$_3$-d. The integral ratio of signal **g** and signal **f** was used to calculated the repeating number (x) and the molecular weight ($M_{n,NMR}$). (B) FTIR spectrum. (C) GPC curve measured using DMF as the eluent with PS standards for calibration.

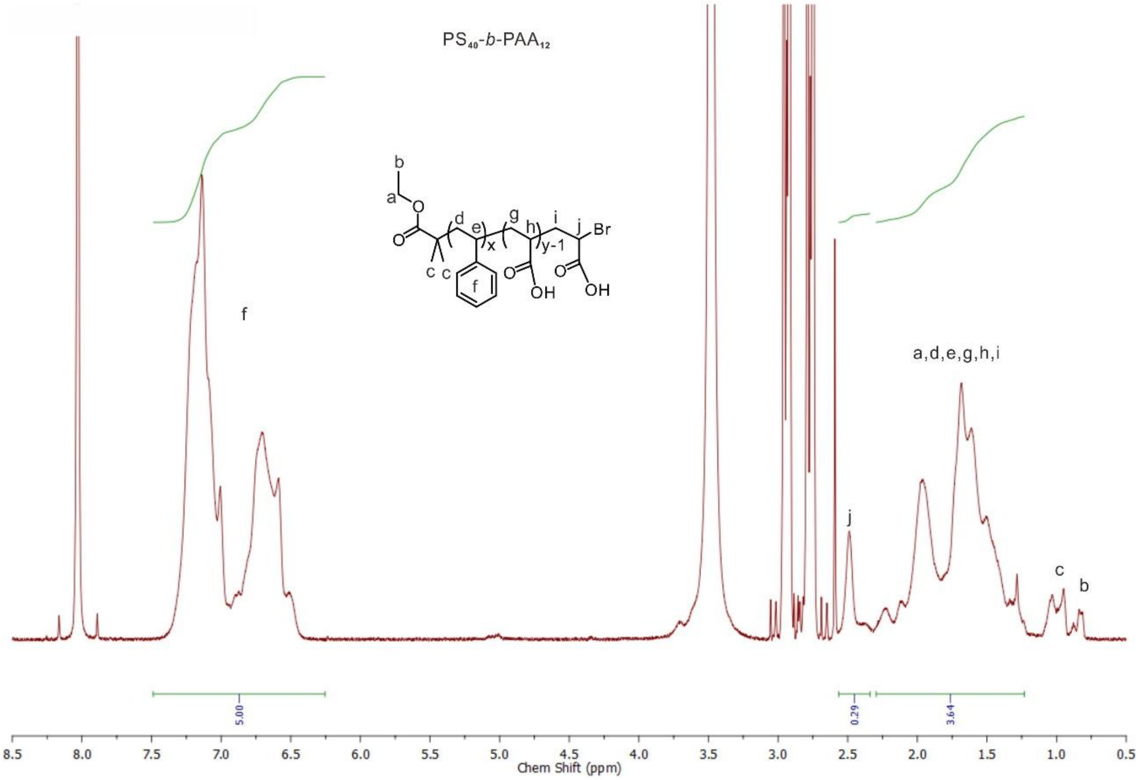

Supplementary Fig. 42. ^{1}H NMR spectrum (700 MHz, 298.3 K) of PS_{40}-*b*-PAA_{12} (**BC**) in DMF-d_7. The repeating number of the PAA block (*y*) was calculated by comparing the integral ratio of signal **f** with signals **a**, **d**, **e**, **g**, **h**, **i**, and **j**.

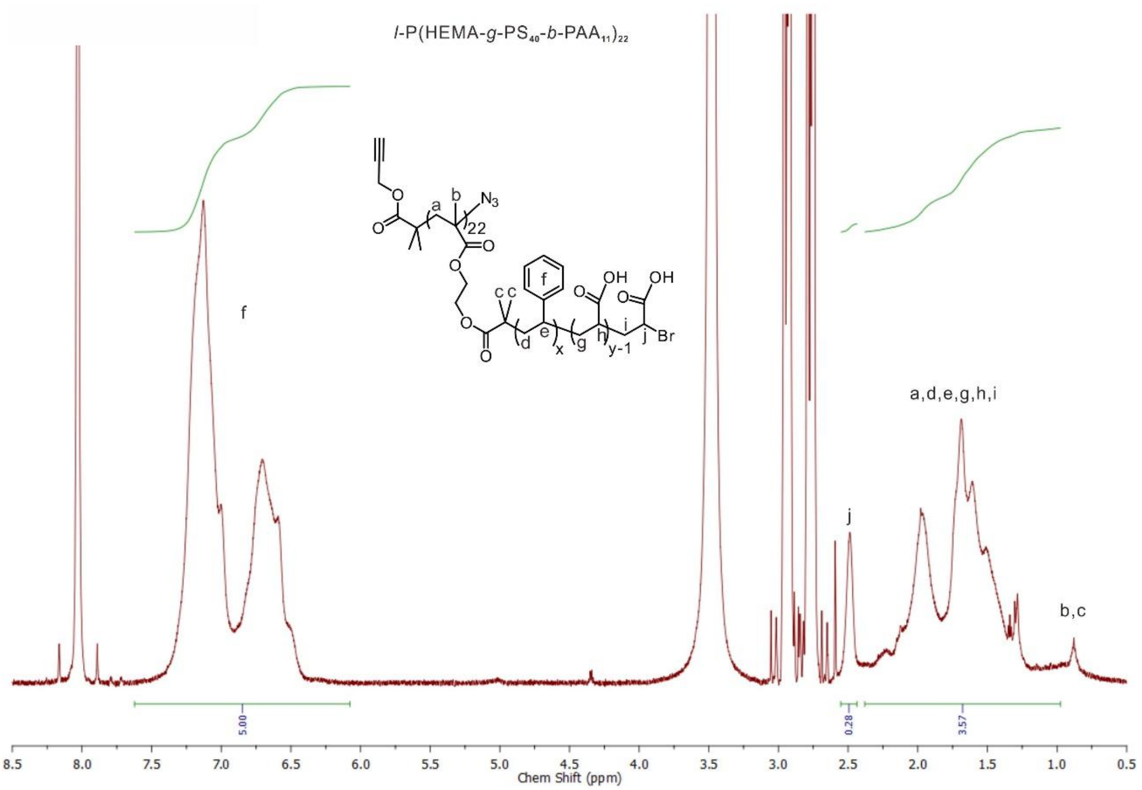

Supplementary Fig. 43. ^{1}H NMR spectrum (700 MHz, 298.3 K) of *l*-P(HEMA-*g*-PS_{40}-*b*-PAA_{11})$_{22}$ (**LB**) in DMF-d_7. The repeating number of the PAA block (*y*) was calculated by comparing the integral ratio of signal **f** with signals **a**, **d**, **e**, **g**, **h**, **i**, and **j**.

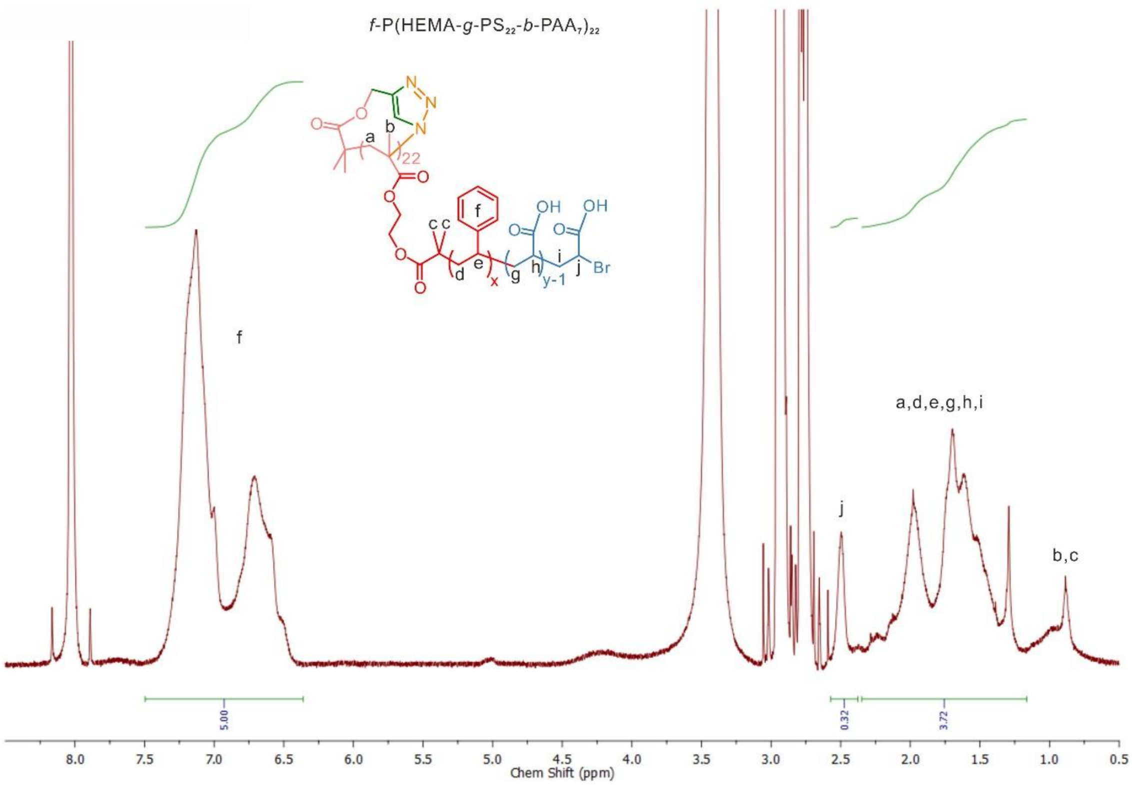

Supplementary Fig. 44. ^{1}H NMR spectrum (700 MHz, 298.3 K) of *f*-P(HEMA-*g*-PS_{22}-*b*-$PAA_7)_{22}$ (**CB-1**) in DMF-d_7. The repeating number of the PAA block (*y*) was calculated by comparing the integral ratio of signal **f** with signals **a**, **d**, **e**, **g**, **h**, **i**, and **j**.

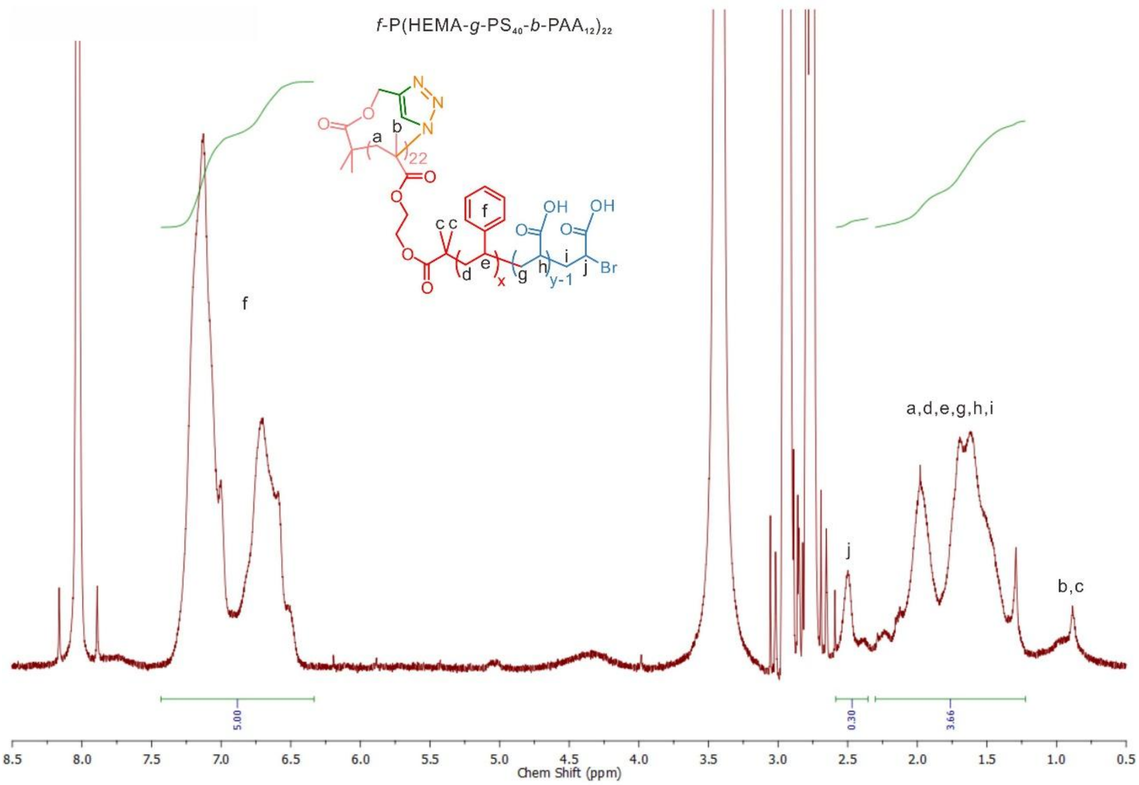

Supplementary Fig. 45. ^{1}H NMR spectrum (700 MHz, 298.3 K) of *f*-P(HEMA-*g*-PS_{40}-*b*-$PAA_{12})_{22}$ (**CB-2**) in DMF-d_7. The repeating number of the PAA block (*y*) was calculated by comparing the integral ratio of signal **f** with signals **a**, **d**, **e**, **g**, **h**, **i**, and **j**.

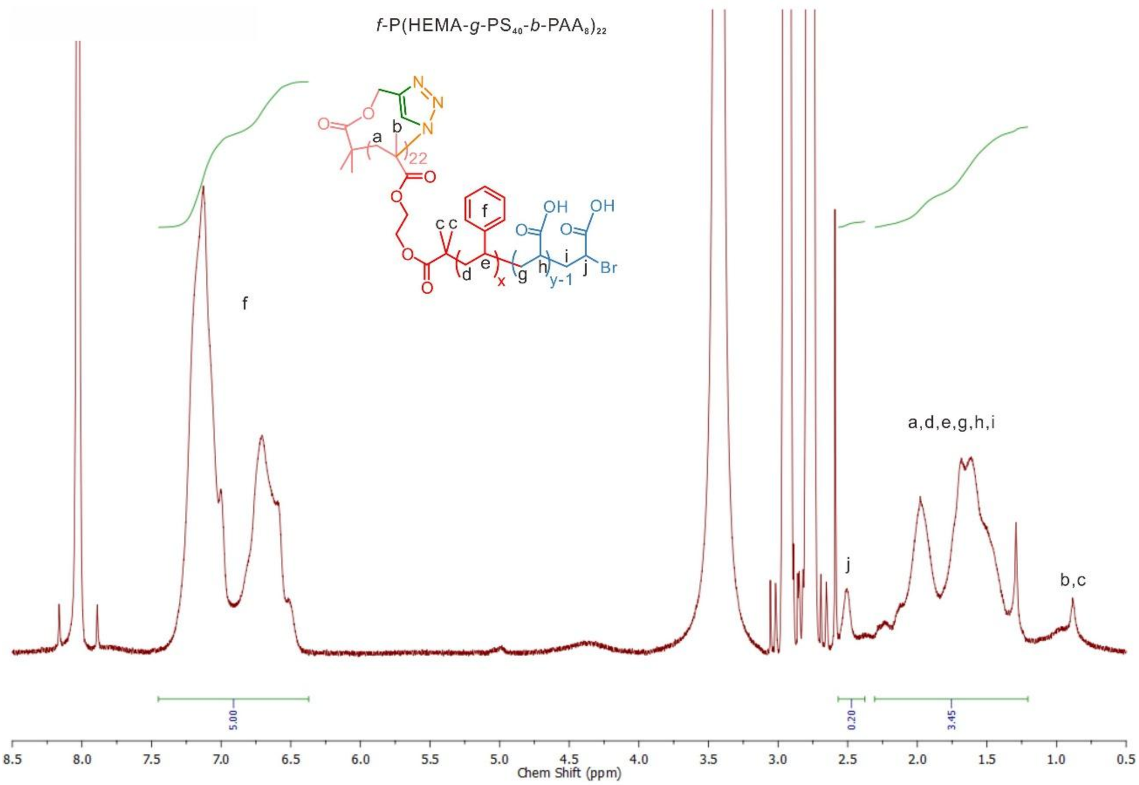

Supplementary Fig. 46. ^{1}H NMR spectrum (700 MHz, 298.3 K) of *f*-P(HEMA-*g*-PS_{40}-*b*-PAA_8)$_{22}$ (**CB-3**) in DMF-d_7. The repeating number of the PAA block (*y*) was calculated by comparing the integral ratio of signal **f** with signals **a**, **d**, **e**, **g**, **h**, **i**, and **j**.

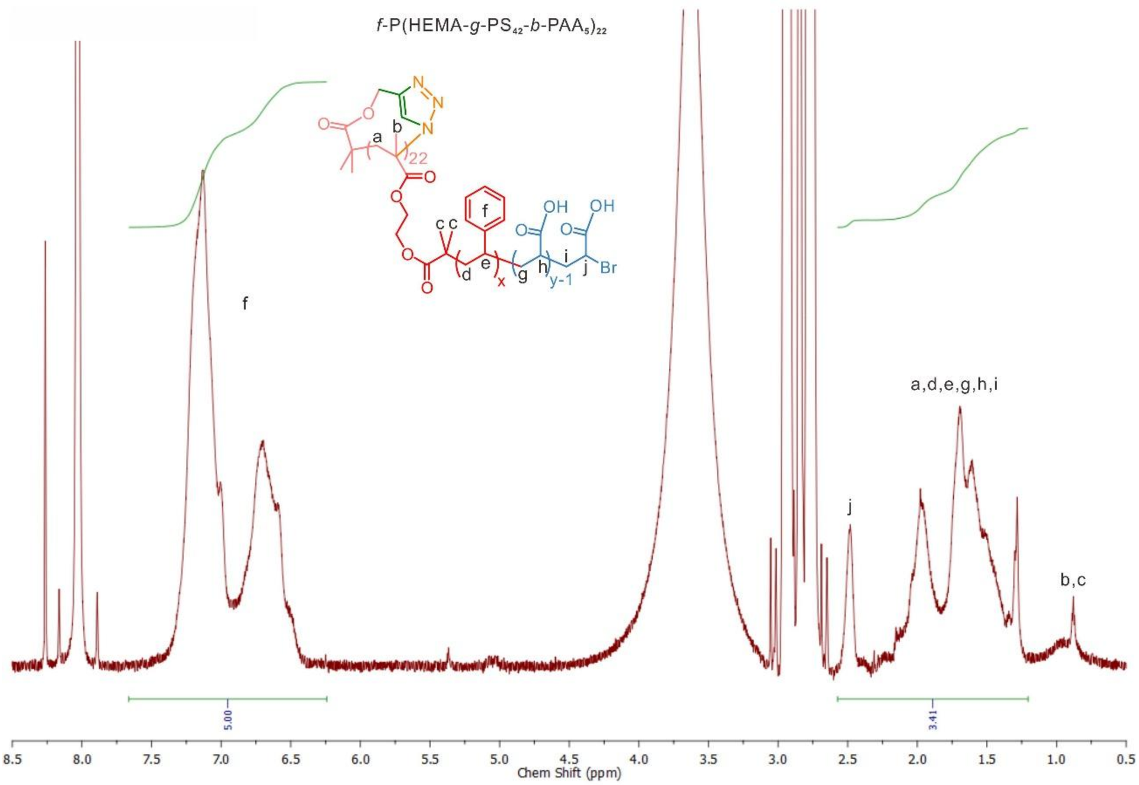

Supplementary Fig. 47. ^{1}H NMR spectrum (700 MHz, 298.3 K) of *f*-P(HEMA-*g*-PS_{42}-*b*-PAA_5)$_{22}$ (**CB-4**) in DMF-d_7. The repeating number of the PAA block (y) was calculated by comparing the integral ratio of signal **f** with signals **a**, **d**, **e**, **g**, **h**, **i**, and **j**.

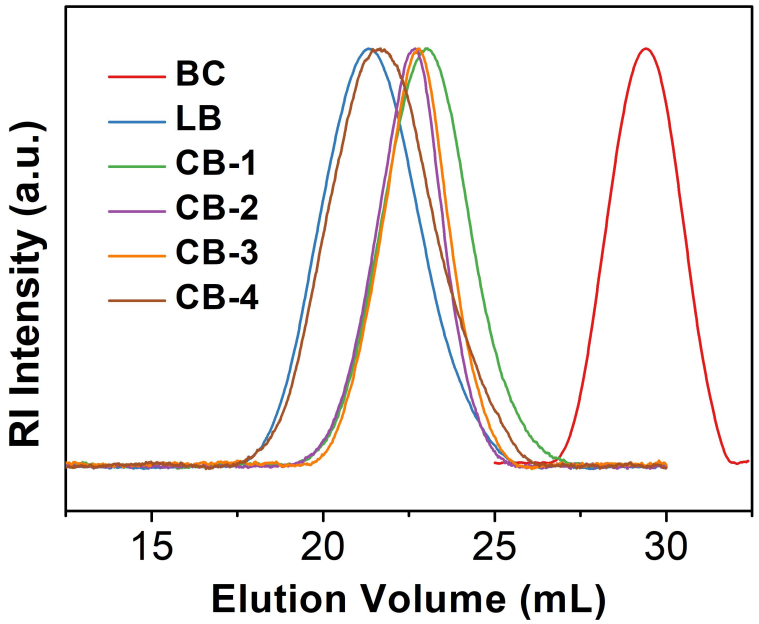

Supplementary Fig. 48. GPC elution curves of block copolymer PS_{40}-*b*-PAA_{12} (**BC**), linear brush polymer *l*-P(HEMA-*g*-PS_{40}-*b*-PAA_{11})$_{22}$ (**LB**), and four cyclic brush polymers: *f*-P(HEMA-*g*-PS_{22}-*b*-PAA_7)$_{22}$ (**CB-1**), *f*-P(HEMA-*g*-PS_{40}-*b*-PAA_{12})$_{22}$ (**CB-2**), *f*-P(HEMA-*g*-PS_{40}-*b*-PAA_8)$_{22}$ (**CB-3**), and *f*-P(HEMA-*g*-PS_{42}-*b*-PAA_5)$_{22}$ (**CB-4**). DMF was used as the eluent and PS standards were employed for calibration.

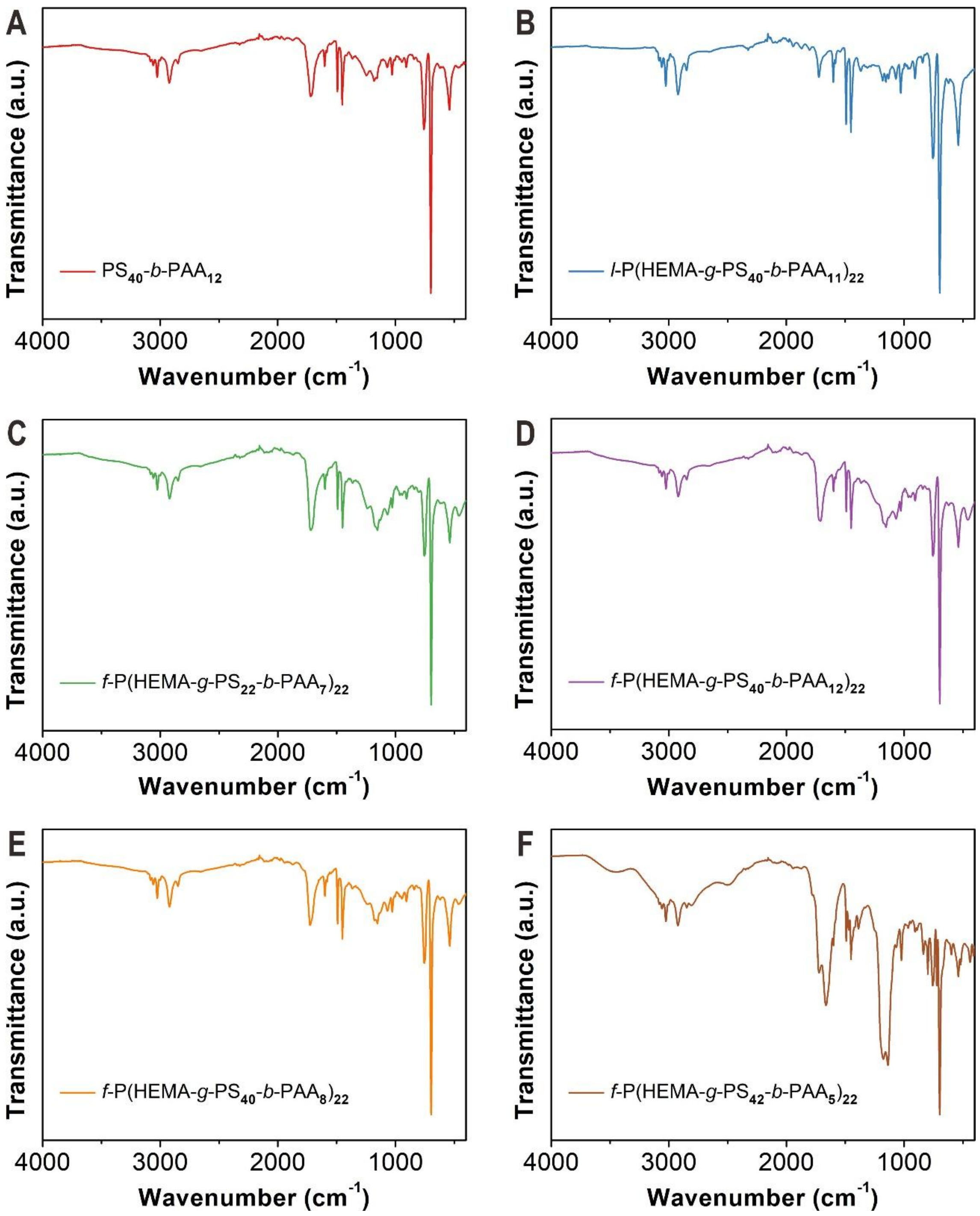

Supplementary Fig. 49. FTIR spectra of (A) PS_{40}-*b*-PAA_{12} (**BC**); (B) *l*-P(HEMA-*g*-PS_{40}-*b*-PAA_{11})$_{22}$ (**LB**); (C) *f*-P(HEMA-*g*-PS_{22}-*b*-PAA_{7})$_{22}$ (**CB-1**); (D) *f*-P(HEMA-*g*-PS_{40}-*b*-PAA_{12})$_{22}$ (**CB-2**); (E) *f*-P(HEMA-*g*-PS_{40}-*b*-PAA_{8})$_{22}$ (**CB-3**); and (F) *f*-P(HEMA-*g*-PS_{42}-*b*-PAA_{5})$_{22}$ (**CB-4**).

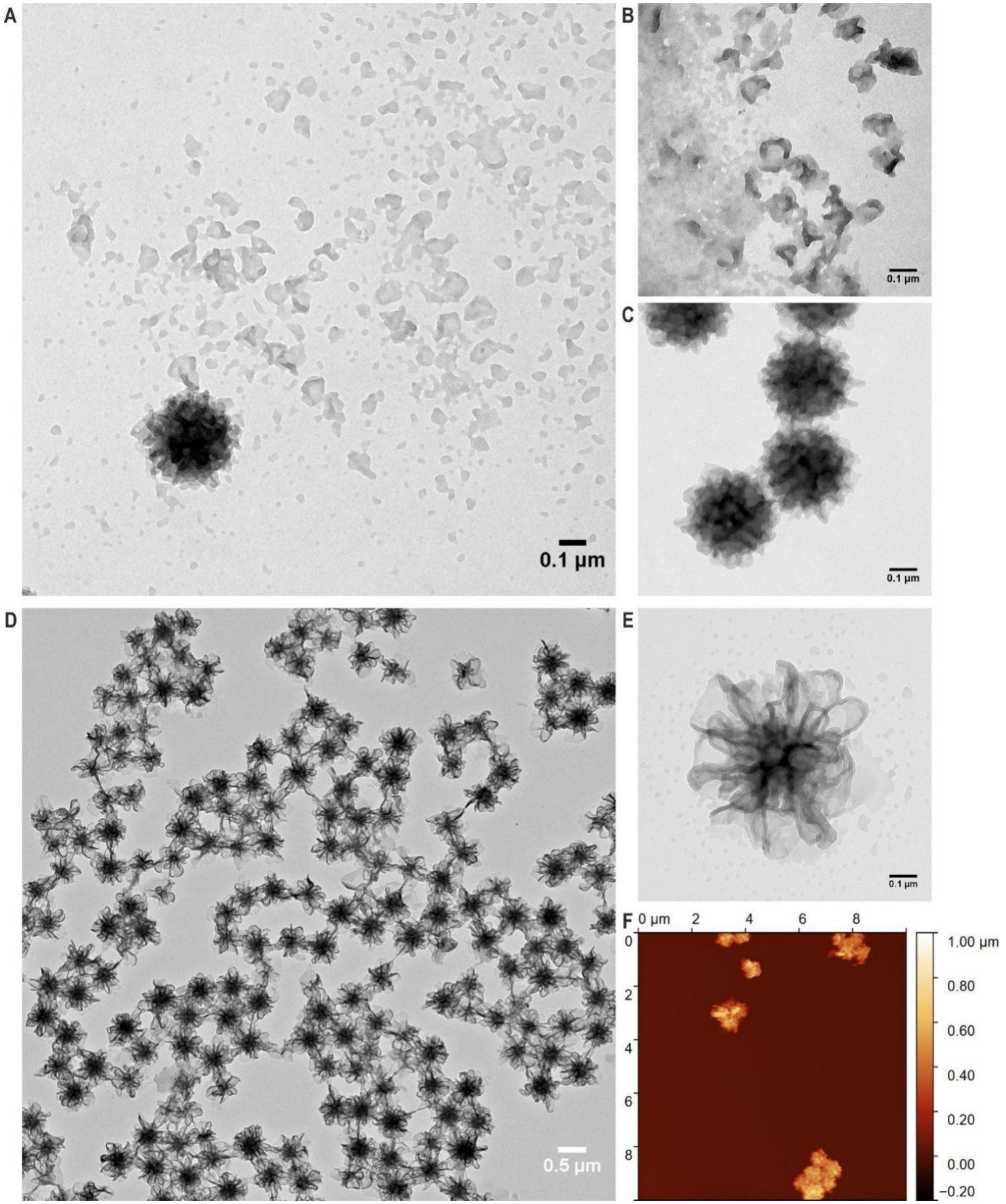

Supplementary Fig. 50. TEM and AFM images showing the self-assembly of PS_{40}-*b*-PAA_{12} (**BC**) in H_2O at 0.1 mg mL^{-1} (A-C) and 0.4 mg mL^{-1} (D-F). Due to its high weight fraction of hydrophobic PS, the block copolymer mainly self-assembled into lamellae (flat or curved bilayers) and vesicles at 0.1 mg mL^{-1}. When the concentration increased to 0.4 mg mL^{-1}, flower-like large compound vesicles formed by aggregation of simple vesicles were obtained. This observation fits with the results reported by other groups in the literature[13].

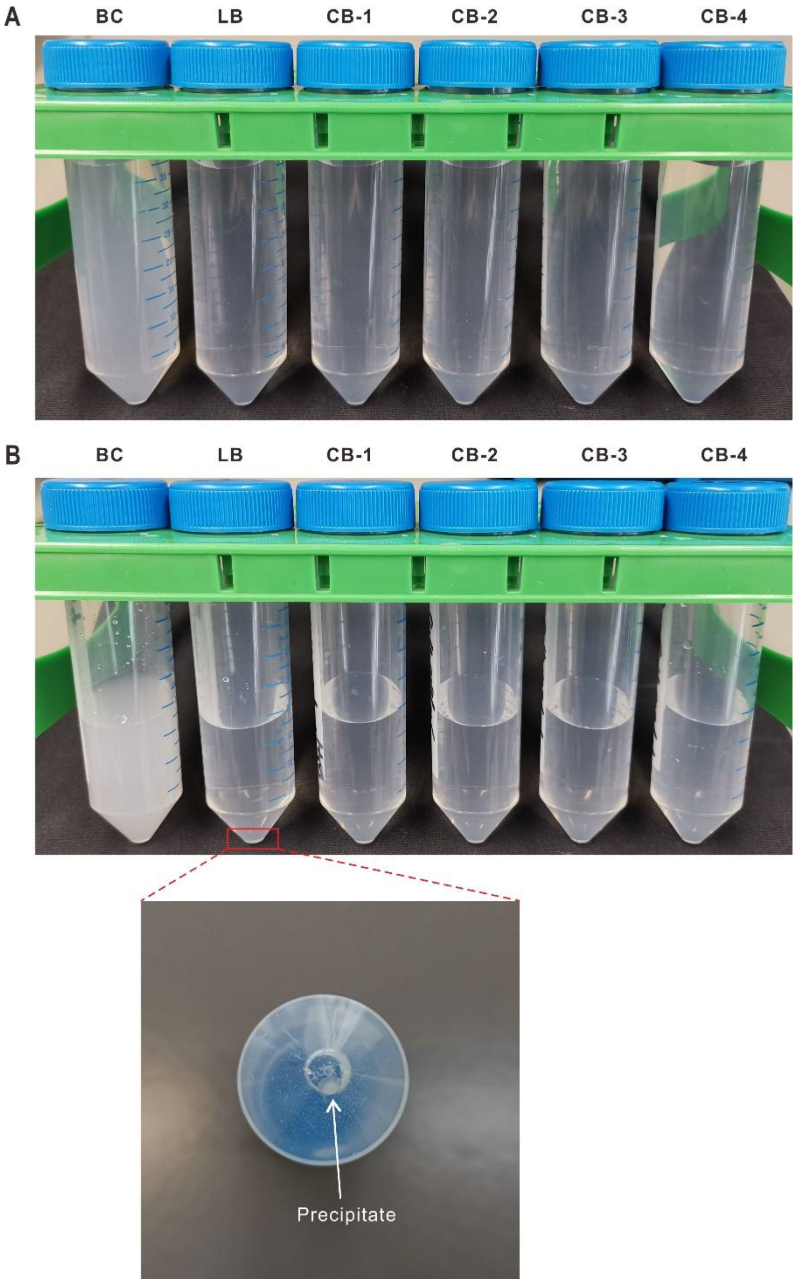

Supplementary Fig. 51. Self-assembly solutions of block copolymer **BC**, linear brush polymer **LB**, and four cyclic brush polymers **CB-1** to **CB-4** at different concentrations: (A) 0.1 mg mL^{-1}, (B) 0.4 mg mL^{-1}.

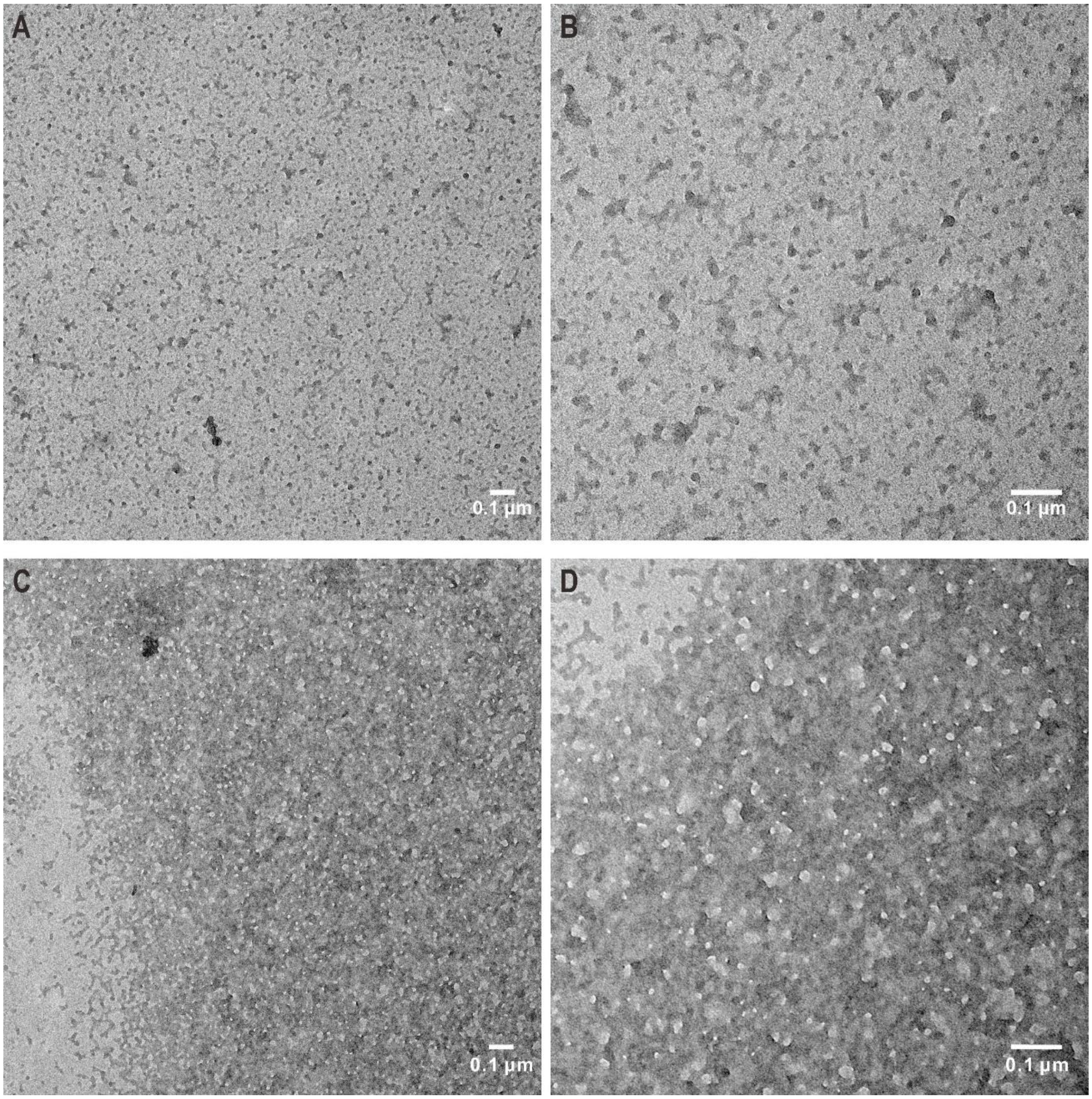

Supplementary Fig. 52. TEM images showing the formation of irregular aggregates from *l*-P(HEMA-*g*-PS_{40}-*b*-PAA_{11})$_{22}$ (**LB**) in H_2O at 0.1 mg mL^{-1} (A and B) and 0.4 mg mL^{-1} (C and D).

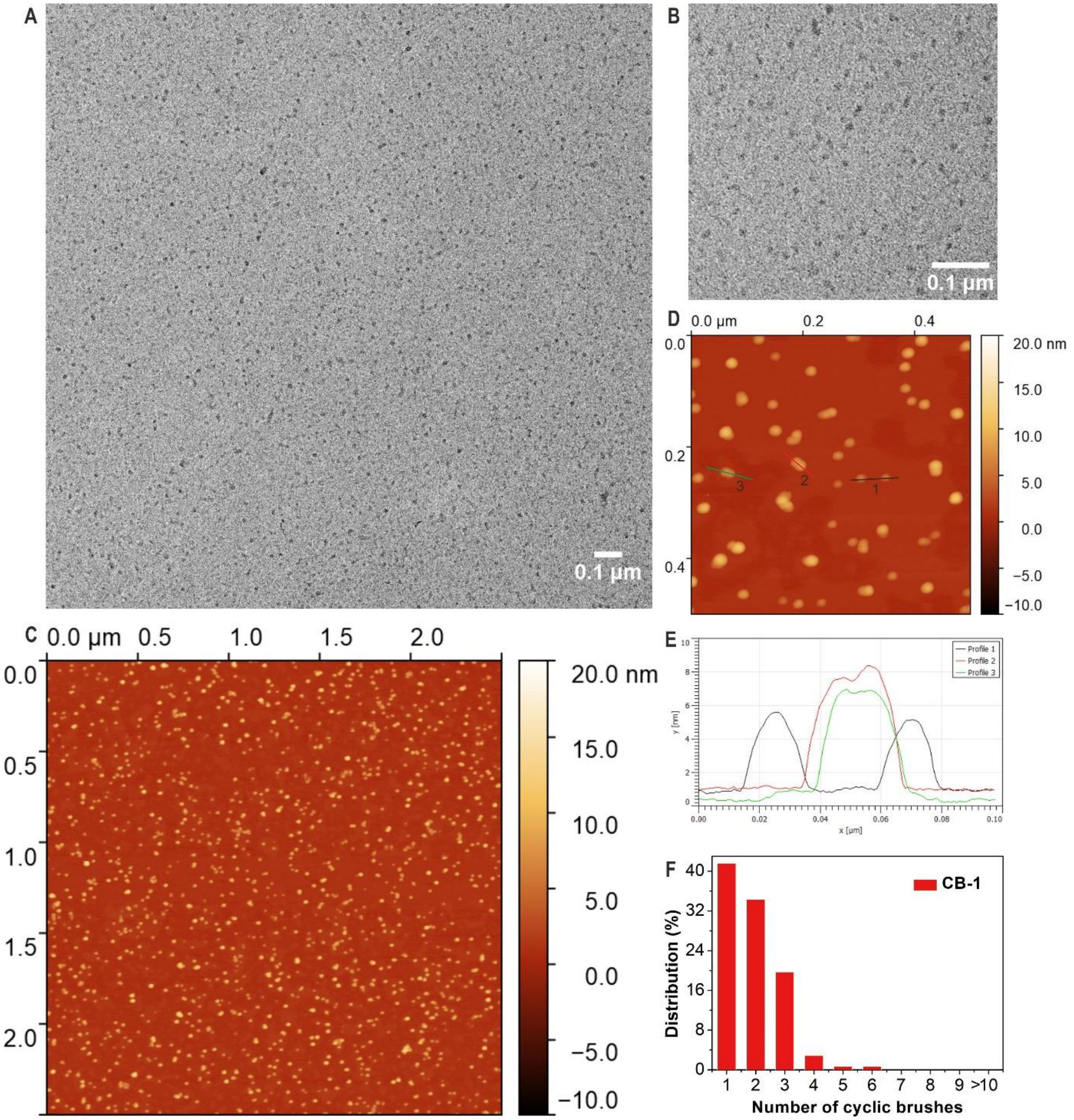

Supplementary Fig. 53. (A and B) TEM and (C and D) AFM images showing the self-assembly of *f*-P(HEMA-*g*-PS_{22}-*b*-PAA_7)$_{22}$ (**CB-1**) in H_2O at 0.1 mg mL^{-1}. (E) The height profiles of the three lines in (D). Profile 1 indicates two single cyclic brushes. Profile 2 and profile 3 are two dimers. (F) Statistical analysis for the number of cyclic brushes in assemblies based on more than 100 nanoobjects in AFM images.

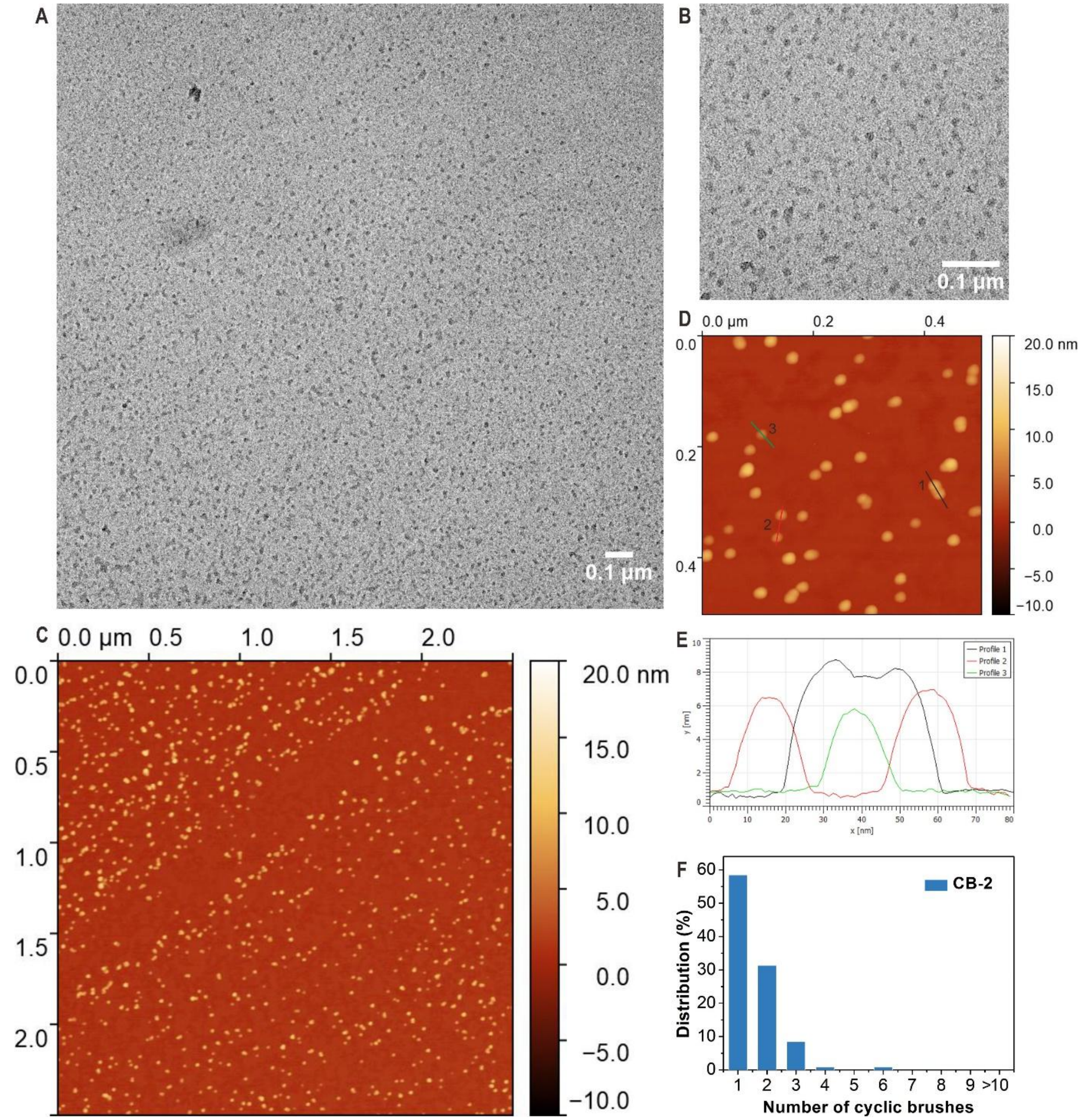

Supplementary Fig. 54. (A and B) TEM and (C and D) AFM images showing the self-assembly of *f*-P(HEMA-*g*-PS_{40}-*b*-PAA_{12})$_{22}$ (**CB-2**) in H_2O at 0.1 mg mL^{-1}. (E) The height profiles of the three lines in (D). Profile 3 indicates a single cyclic brush polymer and profile 1 indicates a trimer. (F) Statistical analysis for the number of cyclic brushes in assemblies based on more than 100 nanoobjects in AFM images.

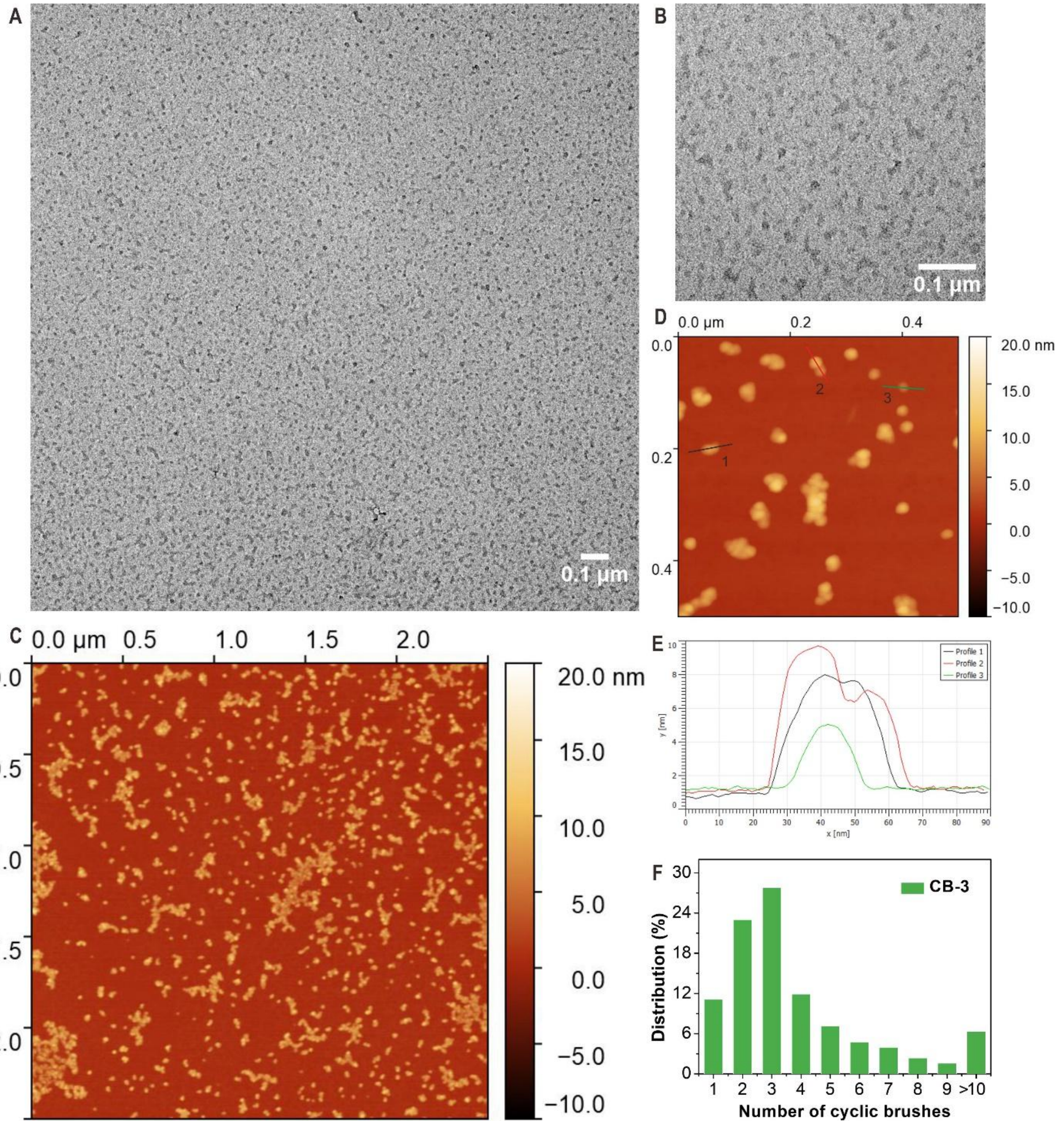

Supplementary Fig. 55. (A and B) TEM and (C and D) AFM images showing the self-assembly of *f*-P(HEMA-*g*-PS_{40}-*b*-PAA_8)$_{22}$ (**CB-3**) in H_2O at 0.1 mg mL^{-1}. (E) The height profiles of the three lines in (D). Profile 3 indicates a single cyclic brush polymer and profile 2 indicates a dimer. (F) Statistical analysis for the number of cyclic brushes in assemblies based on more than 100 nanoobjects in AFM images.

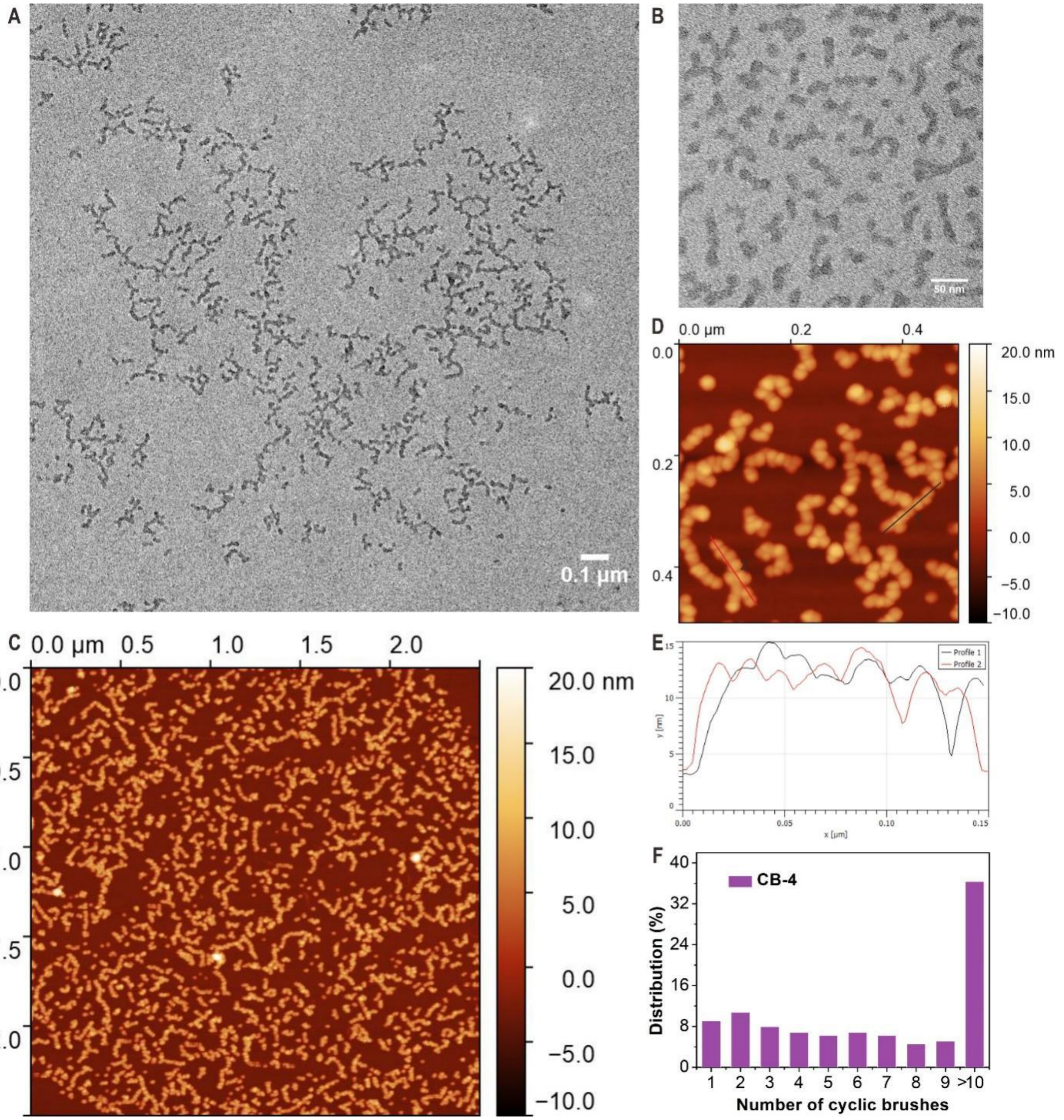

Supplementary Fig. 56. (A and B) TEM and (C and D) AFM images showing the wormlike structures self-assembled from *f*-P(HEMA-*g*-PS_{42}-*b*-PAA_5)$_{22}$ (**CB-4**) in H_2O at 0.1 mg mL^{-1}. (E) The height profiles of the two lines in (D). (F) Statistical analysis for the number of cyclic brushes in assemblies based on more than 100 nanoobjects in AFM images. The results show that more than 36% of nanoobjects are long wormlike structures assembled by > 10 cyclic brushes.

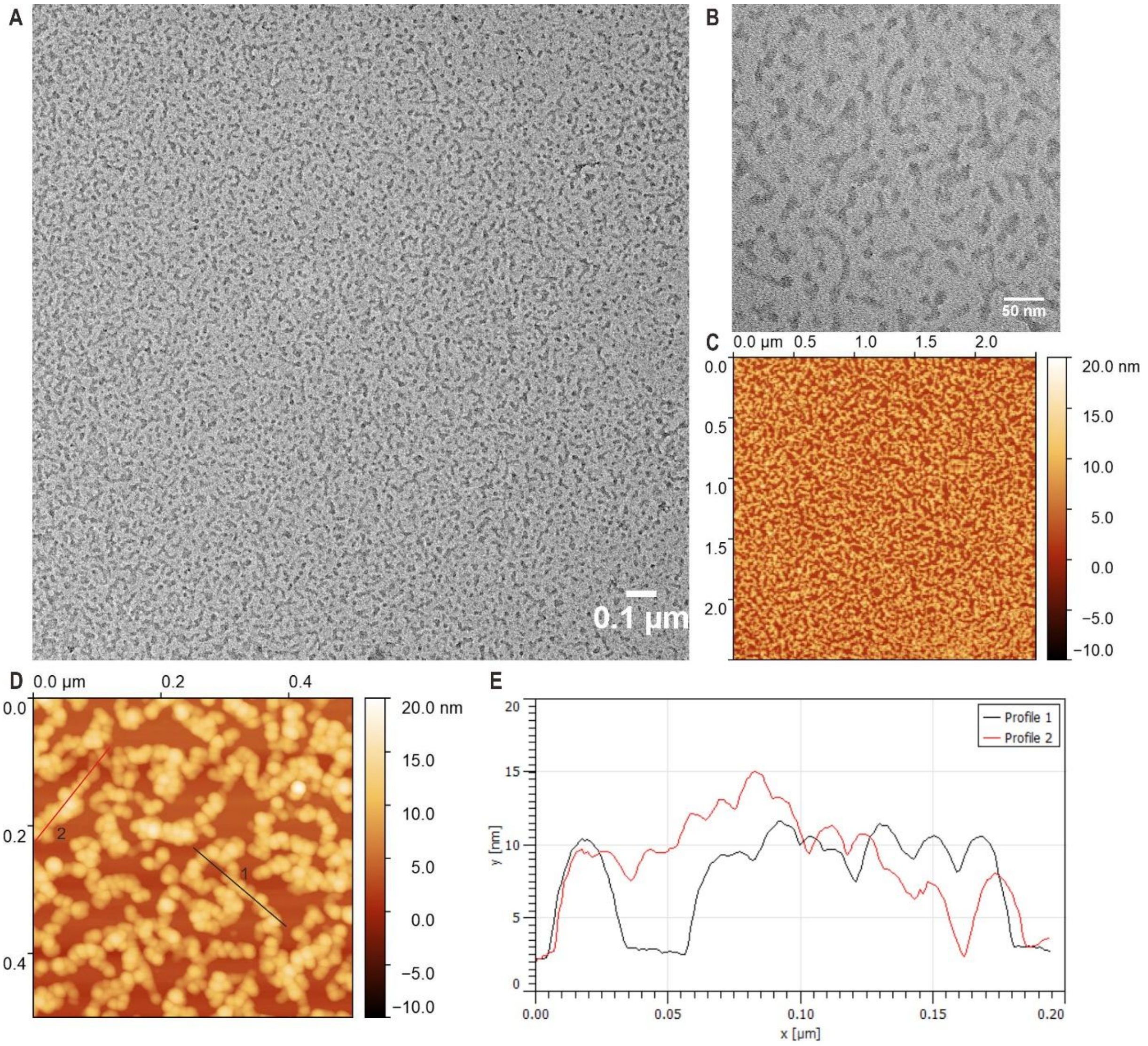

Supplementary Fig. 57. (A and B) TEM and (C and D) AFM images showing the self-assembly of *f*-P(HEMA-*g*-PS$_{22}$-*b*-PAA$_{7}$)$_{22}$ (**CB-1**) in H_2O at 0.4 mg mL^{-1}. (E) The height profiles of the two lines in (D).

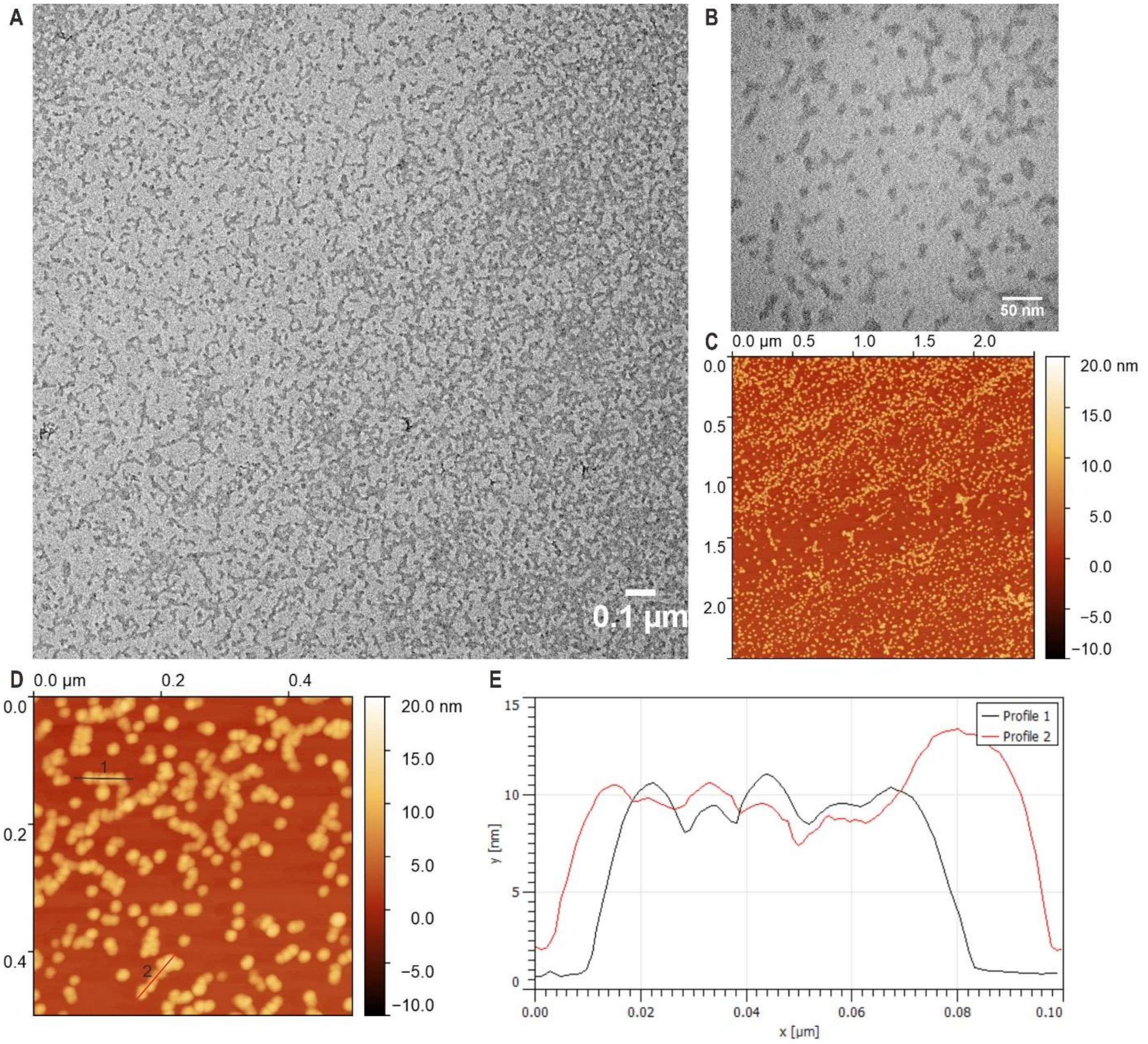

Supplementary Fig. 58. (A and B) TEM and (C and D) AFM images showing the self-assembly of *f*-P(HEMA-*g*-PS$_{40}$-*b*-PAA$_{12}$)$_{22}$ (**CB-2**) in H_2O at 0.4 mg mL^{-1}. (E) The height profiles of the two lines in (D).

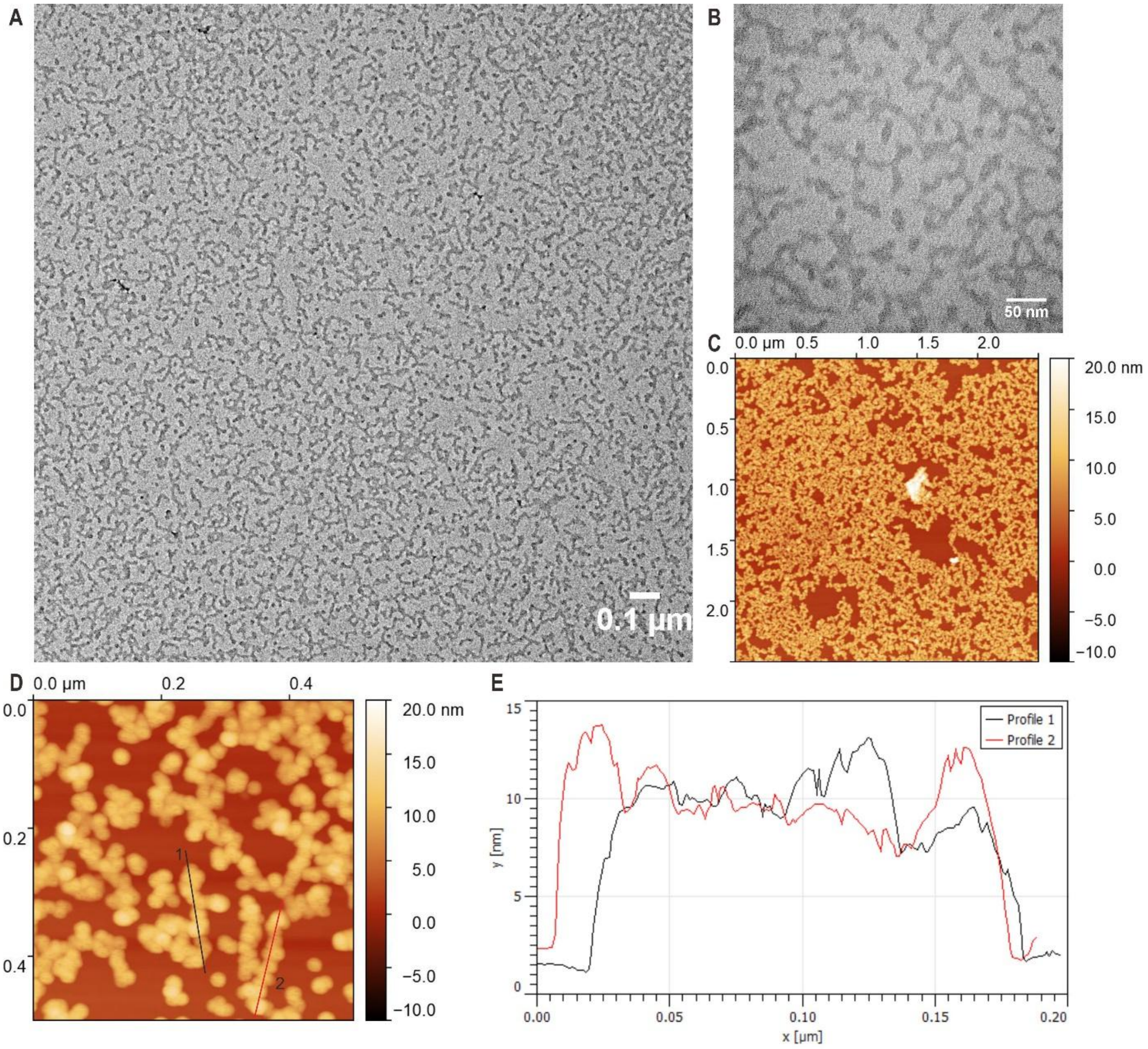

Supplementary Fig. 59. (A and B) TEM and (C and D) AFM images showing the self-assembly of *f*-P(HEMA-*g*-PS_{40}-*b*-PAA_8)$_{22}$ (**CB-3**) in H_2O at 0.4 mg mL^{-1}. (E) The height profiles of the two lines in (D).

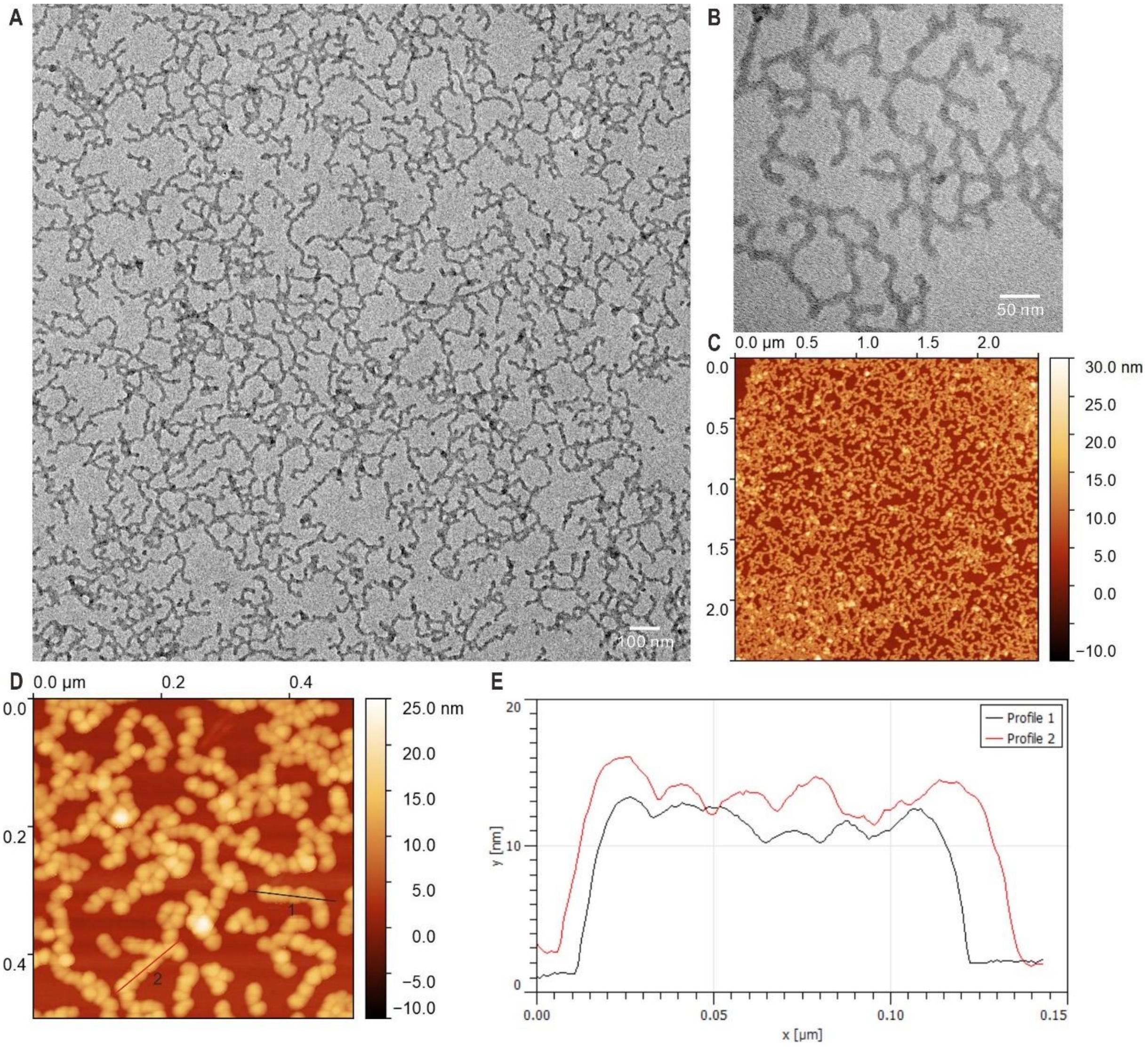

Supplementary Fig. 60. (A and B) TEM and (C and D) AFM images showing the networks self-assembled from *f*-P(HEMA-*g*-PS_{42}-*b*-PAA_5)$_{22}$ (**CB-4**) in H_2O at 0.4 mg mL^{-1}. (E) The height profiles of the two lines in (D).

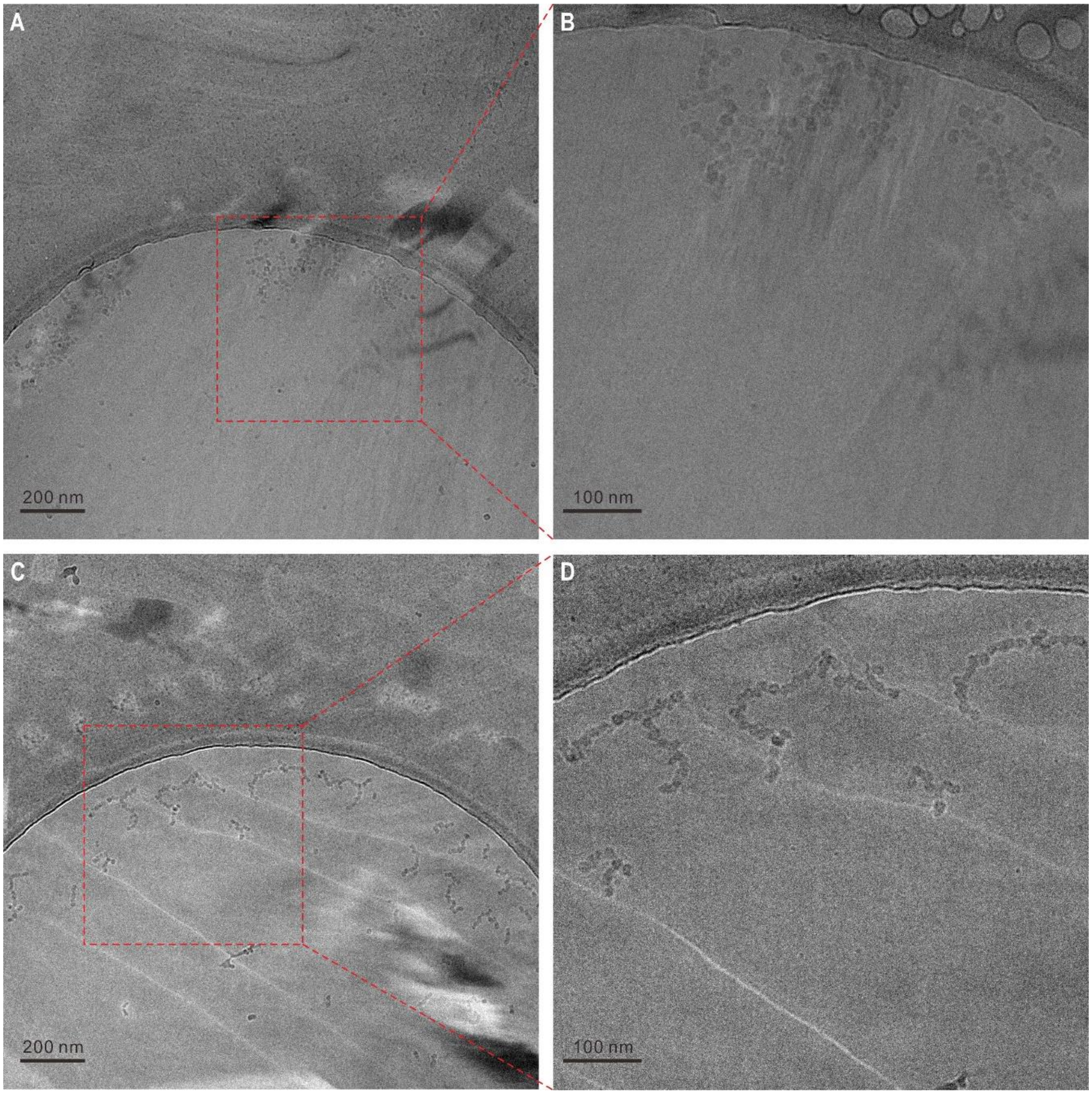

Supplementary Fig. 61. Cryo-TEM images showing the self-assembly of *f*-P(HEMA-*g*-PS_{42}-*b*-PAA_5)$_{22}$ (**CB-4**) in water at different concentrations: (A and B) 0.1 mg mL^{-1}; (C and D) 0.4 mg mL^{-1}. These images are consistent with normal TEM and AFM results and they clearly confirm that branched wormlike assemblies were already formed in solution.

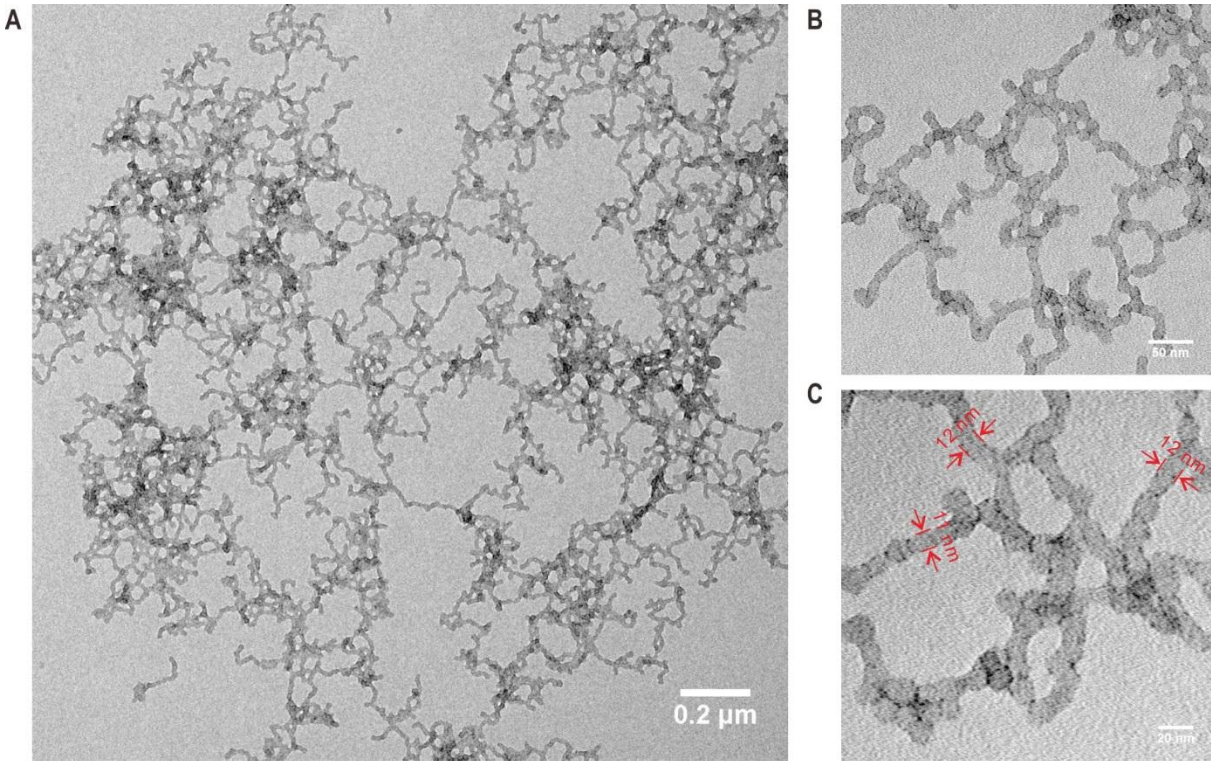

Supplementary Fig. 62. TEM images showing the morphology of stained samples from *f*-P(HEMA-*g*-PS_{42}-*b*-PAA_5)$_{22}$ (**CB-4**, 0.4 mg mL^{-1} in H_2O). The sample was stained with 2% uranyl acetate solution for 45 seconds. After removing the staining solution with a filter paper, the sample was shaken in Milli-Q water for three times (6 seconds for each time). These images show that the washing step couldn't destroy the network-like morphology, confirming the good stability of the branched wormlike assemblies.

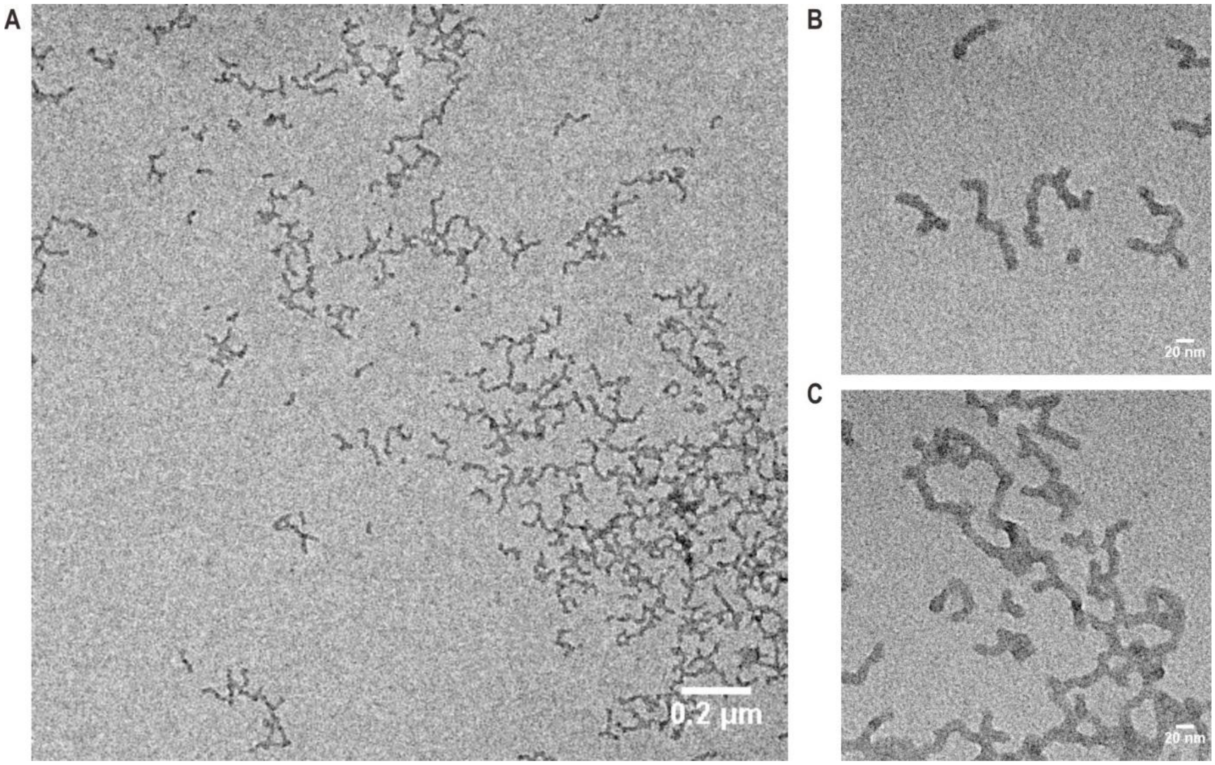

Supplementary Fig. 63. TEM images of the self-assembled solution based on *f*-P(HEMA-*g*-PS_{42}-*b*-$PAA_5)_{22}$ (**CB-4**, 0.4 mg mL^{-1} in H_2O) after sonication for one hour. Although we can see that these assemblies become shorter which is quite understandable, they remained the branched wormlike shape. The shortening of these structures after sonication also confirms from a different perspective that the branched wormlike assemblies in Fig. 3g were not generated during the sample preparation process. Otherwise, the length of the structures after sonication should not change.

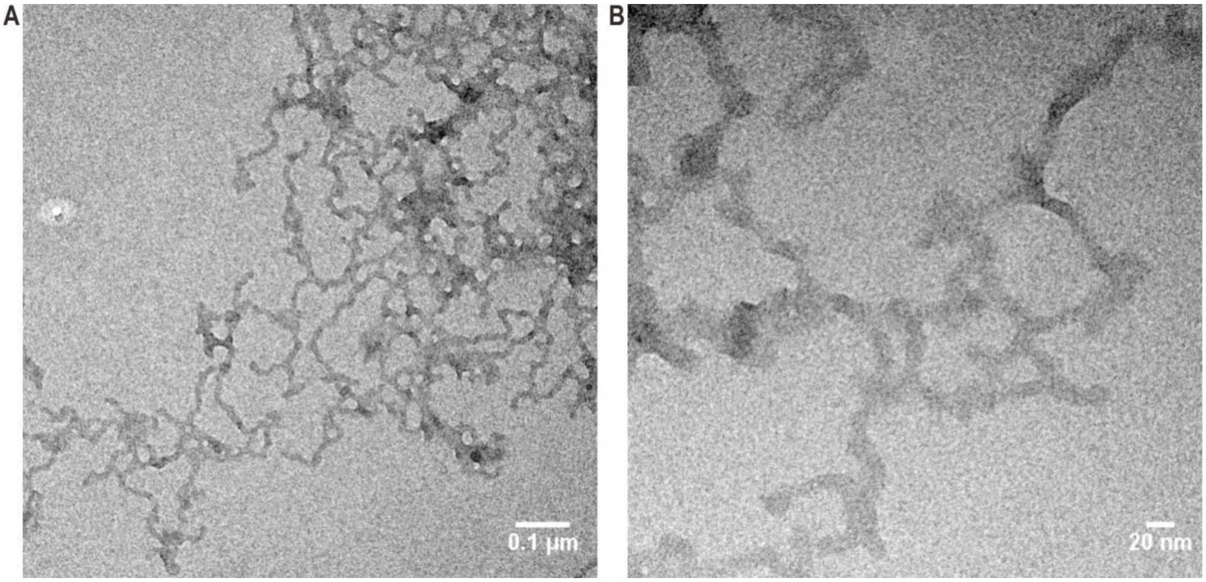

Supplementary Fig. 64. TEM images of the self-assembled solution based on *f*-P(HEMA-*g*-PS_{42}-*b*-$PAA_5)_{22}$ (**CB-4**, 0.4 mg mL^{-1} in H_2O) after storage at room temperature for two months.

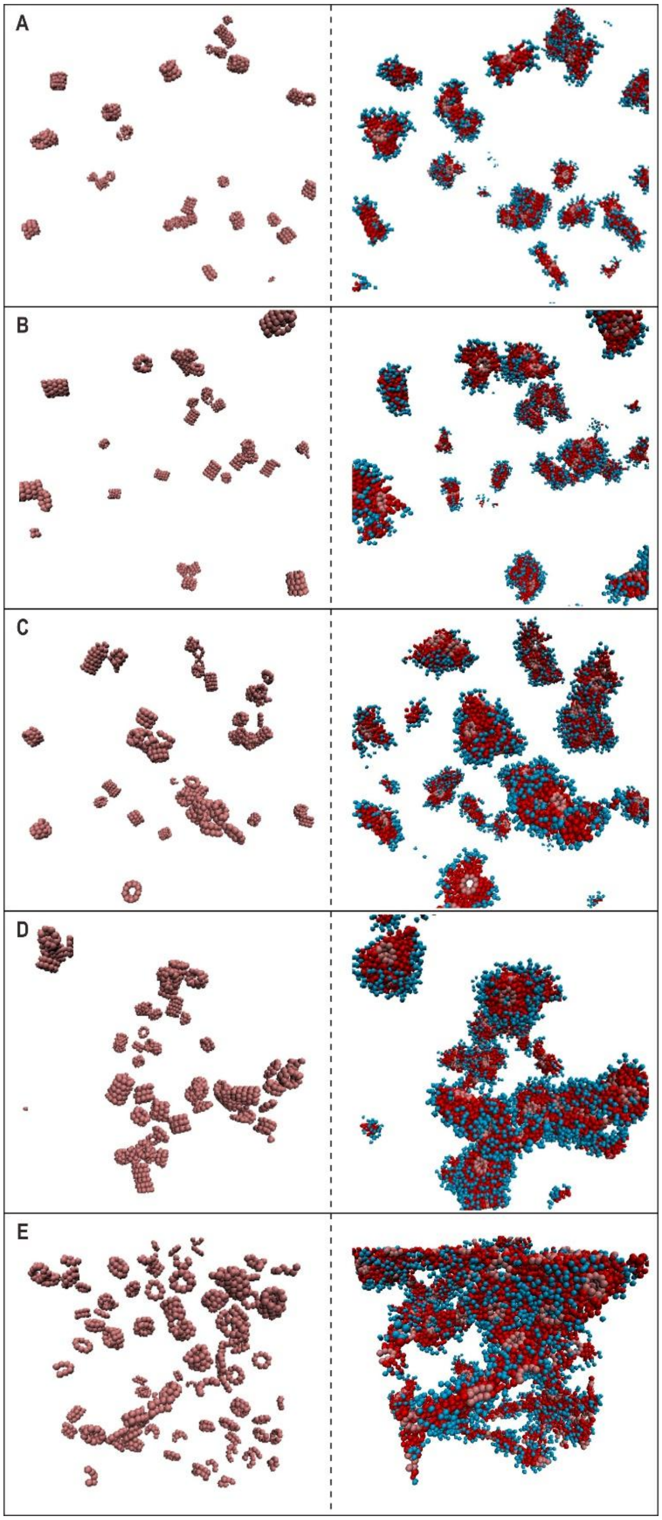

Supplementary Fig. 65. Molecular simulation snapshots showing the self-assembly of cyclic brush polymers at increased concentrations. The left snapshots show only the hydrophobic cyclic backbones and right the full molecules. The data is shown for the five different concentrations, ρ = 0.0002σ^{-3} (A), 0.00035σ^{-3} (B), 0.0005σ^{-3} (C), 0.0009σ^{-3} (D), and 0.0017σ^{-3} (E).

Supplementary Tables

Supplementary Table 1. Synthesis and characterization of linear and cyclic macroinitiators.

Entry	$M_{n,NMR}$ [a)] (g mol^{-1})	$M_{n,GPC}$ [b)] (g mol^{-1})	M_w [b)] (g mol^{-1})	M_p [b)] (g mol^{-1})	V_p [b)] (mL)	$Đ$ [b)] (M_w/M_n)
l-P(HEMA-*Br*)$_{22}$	6300	8700	15200	13200	27.11	1.75
f-P(HEMA-*Br*)$_{22}$	6300	8800	13000	7400	27.95	1.47

[a)] Determined by ^{1}H NMR. [b)] Determined by GPC using DMF as eluent and PMMA as standards.

Supplementary Table 2. Synthesis and characterization of PS as well as linear and cyclic brush polymers with PS side chains.

Ratio [a)] I/C/L/M	Entry	$M_{n,NMR}$ [b)] (g mol^{-1})	$M_{n,GPC}$ [c)] (g mol^{-1})	M_w [c)] (g mol^{-1})	M_p [c)] (g mol^{-1})	V_p [c)] (mL)	$Đ$ [c)] (M_w/M_n)
1:1:2:400	PS_{40}-*Br*	4400	3700	5000	4300	29.51	1.34
1:1:2:600	*l*-P(HEMA-*g*-PS_{40}-*Br*)$_{22}$	98000	80300	99200	92900	22.74	1.24
1:1:2:600	*f*-P(HEMA-*g*-PS_{22}-*Br*)$_{22}$	56700	45600	63200	52700	23.77	1.39
1:1:2:600	*f*-P(HEMA-*g*-PS_{40}-*Br*)$_{22}$	98000	59000	79600	71000	23.17	1.35
1:1:2:600	*f*-P(HEMA-*g*-PS_{42}-*Br*)$_{22}$	102500	63300	80800	67700	23.21	1.28

[a)] Molar ratio of initiator/catalyst/ligand/monomer. [b)] Determined by ^{1}H NMR. [c)] Determined by GPC using DMF as eluent and PS as standards.

Supplementary Table 3. Synthesis and characterization of block copolymer as well as linear and cyclic brush polymers with PS-b-PAA side chains.

Ratio [a] I/C/L/M	Entry	$M_{n,NMR}$ [b] (g/mol)	f_{PAA} (wt%)	$M_{n,GPC}$ [c] (g/mol)	M_w [c] (g/mol)	M_p [c] (g/mol)	V_p [c] (mL)	$Đ$ [c] (M_w/M_n)
1:2:4:400	PS_{40}-*b*-PAA_{12} (**BC**)	5300	16.3	3800	5400	4500	29.41	1.42
1:2:4:400	*l*-P(HEMA-*g*-PS_{40}-*b*-PAA_{11})$_{22}$ (**LB**)	115400	15.1	151900	225500	180300	21.33	1.48
1:2:4:400	*f*-P(HEMA-*g*-PS_{22}-*b*-PAA_{7})$_{22}$ (**CB-1**)	67800	16.4	76200	92800	81200	23.04	1.22
1:2:4:400	*f*-P(HEMA-*g*-PS_{40}-*b*-PAA_{12})$_{22}$ (**CB-2**)	117000	16.3	96400	113300	93600	22.73	1.18
1:2:4:400	*f*-P(HEMA-*g*-PS_{40}-*b*-PAA_{8})$_{22}$ (**CB-3**)	110700	11.5	91000	106200	90800	22.79	1.17
1:2:8:800	*f*-P(HEMA-*g*-PS_{42}-*b*-PAA_{5})$_{22}$ (**CB-4**)	110400	7.2	129300	191600	169700	21.45	1.48

[a] Molar ratio of initiator/catalyst/ligand/monomer. [b] Determined by ^{1}H NMR. [c] Determined by GPC using DMF as eluent and PS as standards.

Supplementary References

1. Cheng, Y.L. et al. Nano-Sized Sunflower Polycations As Effective Gene Transfer Vehicles. *Small* **12**, 2750-2758 (2016).
2. Tanner, J.E. Use of Stimulated Echo in Nmr-Diffusion Studies. *J. Chem. Phys.* **52**, 2523-2526 (1970).
3. Wu, D.H., Chen, A.D. & Johnson, C.S. An Improved Diffusion-Ordered Spectroscopy Experiment Incorporating Bipolar-Gradient Pulses. *J. Magn. Reson., Ser A* **115**, 260-264 (1995).
4. Morris, K.F. & Johnson, C.S. Diffusion-Ordered 2-Dimensional Nuclear-Magnetic-Resonance Spectroscopy. *J. Am. Chem. Soc.* **114**, 3139-3141 (1992).
5. Pronk, S. et al. GROMACS 4.5: a high-throughput and highly parallel open source molecular simulation toolkit. *Bioinformatics* **29**, 845-854 (2013).
6. Bussi, G., Donadio, D. & Parrinello, M. Canonical sampling through velocity rescaling. *J. Chem. Phys.* **126**, 014101 (2007).
7. Parrinello, M. & Rahman, A. Crystal-Structure and Pair Potentials - a Molecular-Dynamics Study. *Phys. Rev. Lett.* **45**, 1196-1199 (1980).
8. Essmann, U. et al. A Smooth Particle Mesh Ewald Method. *J. Chem. Phys.* **103**, 8577-8593 (1995).
9. Horta, B.A.C. et al. A GROMOS-Compatible Force Field for Small Organic Molecules in the Condensed Phase: The 2016H66 Parameter Set. *J. Chem. Theory Comput.* **12**, 3825-3850 (2016).
10. Wu, Y.J., Tepper, H.L. & Voth, G.A. Flexible simple point-charge water model with improved liquid-state properties. *J. Chem. Phys.* **124**, 024503 (2006).
11. Kremer, K. & Grest, G.S. Dynamics of Entangled Linear Polymer Melts - a Molecular-Dynamics Simulation. *J. Chem. Phys.* **92**, 5057-5086 (1990).
12. Plimpton, S. Fast Parallel Algorithms for Short-Range Molecular-Dynamics. *J. Comput. Phys.* **117**, 1-19 (1995).
13. Mai, Y.Y. & Eisenberg, A. Self-assembly of block copolymers. *Chem. Soc. Rev.* **41**, 5969-5985 (2012).
