# Supplementary material for: Polymer cyclization for the emergence of hierarchical nanostructures
Source: Nat Commun. 2021 Jun 25;12:3959. doi: 10.1038/s41467-021-24222-5 (PMC8233313; doi:10.1038/s41467-021-24222-5)
Supplement: Supplementary file 3 — Description of Additional Supplementary Files. [file 41467_2021_24222_MOESM3_ESM.pdf]

Description of Additional Supplementary Files

File Name: Supplementary Movie 1
Description: Molecular simulation animation showing the self-assembly of cyclic brush polymers at ρ = $0.0002\sigma^{-3}$. This animation shows only the hydrophobic cyclic backbones.

File Name: Supplementary Movie 2
Description: Molecular simulation animation showing the self-assembly of cyclic brush polymers at ρ = $0.0002\sigma^{-3}$. This animation shows the full molecules.

File Name: Supplementary Movie 3
Description: Molecular simulation animation showing the self-assembly of cyclic brush polymers at ρ = $0.00035\sigma^{-3}$. This animation shows only the hydrophobic cyclic backbones.

File Name: Supplementary Movie 4
Description: Molecular simulation animation showing the self-assembly of cyclic brush polymers at ρ = $0.00035\sigma^{-3}$. This animation shows the full molecules.

File Name: Supplementary Movie 5
Description: Molecular simulation animation showing the self-assembly of cyclic brush polymers at ρ = $0.0005\sigma^{-3}$. This animation shows only the hydrophobic cyclic backbones.

File Name: Supplementary Movie 6
Description: Molecular simulation animation showing the self-assembly of cyclic brush polymers at ρ = $0.0005\sigma^{-3}$. This animation shows the full molecules.

File Name: Supplementary Movie 7
Description: Molecular simulation animation showing the self-assembly of cyclic brush polymers at ρ = $0.0009\sigma^{-3}$. This animation shows only the hydrophobic cyclic backbones.

File Name: Supplementary Movie 8
Description: Molecular simulation animation showing the self-assembly of cyclic brush polymers at ρ = $0.0009\sigma^{-3}$. This animation shows the full molecules.

File Name: Supplementary Movie 9
Description: Molecular simulation animation showing the self-assembly of cyclic brush polymers at ρ = $0.0017\sigma^{-3}$. This animation shows only the hydrophobic cyclic backbones.

File Name: Supplementary Movie 10
Description: Molecular simulation animation showing the self-assembly of cyclic brush polymers at ρ = $0.0017\sigma^{-3}$. This animation shows the full molecules.
